# Ubiquitination plays a key role in an insecticide resistance fitness cost

**DOI:** 10.1073/pnas.2536664123

**Published:** 2026-07-30

**Authors:** Jinyu Hu, Buli Fu, Qi Su, Xindi Guo, Chengjia Zhang, Qimei Tan, Rong Zhang, Hantang Lu, Peipan Gong, Shaonan Liu, Xuguo Zhou, Ralf Nauen, Youjun Zhang, Chris Bass, Xin Yang

**Affiliations:** ^a^ State Key Laboratory of Vegetable Biobreeding, Department of Plant Protection, Institute of Vegetables and Flowers, Chinese Academy of Agricultural Sciences, Beijing 100081, China; ^b^ The Ministry of Agriculture and Rural Affairs Key Laboratory of Integrated Pest Management of Tropical Crops, Environment and Plant Protection Institute, Chinese Academy of Tropical Agricultural Sciences, Haikou 571101, China; ^c^ Ministry of Agriculture and Rural Affairs Key Laboratory of Sustainable Crop Production in the Middle Reaches of the Yangtze River (Co-construction by Ministry and Province), Hubei Engineering Technology Center for Forewarning and Management of Agricultural and Forestry Pests, College of Agriculture, Yangtze University, Jingzhou 434025, Hubei, China; ^d^ Institute of Food and Nutrition Development, Ministry of Agriculture and Rural Affairs, Beijing 100081, China; ^e^ Department of Entomology, School of Integrative Biology, College of Liberal Arts and Sciences, University of Illinois Urbana-Champaign, Urbana, IL 61801-3795; ^f^Crop Science Division, Bayer AG, Monheim 40789, Germany; ^g^ Department of Biosciences, College of Life and Environmental Sciences, University of Exeter, Penryn TR10 9FE, Cornwall, United Kingdom

**Keywords:** ubiquitination, E1-1/E2-3/TRIM37 cascade, E3 ubiquitin ligase, insecticide resistance, reproductive cost

## Abstract

Ubiquitination is an essential posttranslational modification in eukaryotes, which plays a fundamental role in numerous important biological functions. However, the role of ubiquitination in the evolution of adaptive traits in insects remains poorly understood. Here, we reveal the role of ubiquitination in insecticide resistance and its associated fitness cost in the whitefly pest, *Bemisia tabaci*. We identify the RING-type E3 ubiquitin ligase TRIM37 in *B. tabaci* and show its promotion of ubiquitination in vivo and in vitro. We further demonstrate that this ligase binds to other ubiquitin enzymes as part of the E1-1/E2-3/TRIM37 ubiquitination cascade, negatively regulating the transcription factor CREB by marking it for degradation. CREB, in turn, positively regulates the insecticide resistance gene *CYP6CM1* while negatively regulating the key oogenesis gene *Vasa*. Downregulation of TRIM37 in neonicotinoid-resistant *B. tabaci* strains enhances insecticide detoxification via *CYP6CM1* upregulation, but concurrently incurs a reproductive fitness cost due to downstream *Vasa* suppression. Our findings advance understanding of the insect ubiquitination system and its role in adaptive processes, while offering insights for sustainable pest control strategies.

## Significance

Ubiquitination is a key posttranslational modification in eukaryotes that plays a critical role in diverse cellular processes by regulating protein function. Here, we show that the E1-1/E2-3/TRIM37 ubiquitination cascade in the global whitefly pest, *Bemisia tabaci*, regulates a transcription factor (CREB), leading to insecticide resistance and its associated fitness cost. These results illustrate the posttranslational regulation of the xenobiotic response in insects and provide potential new targets for control strategies against a notorious crop pest.

Posttranslational modifications represent important mechanisms by which organisms adapt to environmental perturbations and frequently modulate signaling networks to maintain cellular homeostasis (1–3). Ubiquitination is a pervasive posttranslational modification that plays a fundamental role in hormone responses, development, immunity, and stress resistance (4–7). The ubiquitin–proteasome system (UPS) represents a dynamically controlled, highly intricate, and well conserved pathway in cells (8–11). The system comprises various enzymes and accompanying elements, such as ubiquitin (Ub), Ub-activating enzyme (E1), Ub-conjugating enzyme (E2), Ub ligase (E3), substrate proteins, and proteasomes (12). Employing these enzymes and their components, the UPS ubiquitinates specific protein substrates and subsequently degrades these modified substrates via a nonlysosomal pathway. This process entails two key steps: initially, Ub, a 76-amino acid protein, is conveyed to the E2 carrier protein. With the assistance of E3, the E2 carrier then attaches a single Ub molecule or a poly-Ub chain to the targeted substrate destined for degradation. Second, the substrate marked with Ub molecules is identified and broken down by the 26S proteasome, during which Ub moieties are liberated for subsequent reuse (13). A handful of E1 enzymes cooperate with 30 different E2 enzymes and about 600 distinct E3 enzymes to catalyze the ubiquitination of hundreds of substrate proteins (13), which modulate a wide range of cellular activities, including signal transduction, transcriptional regulation, DNA damage response, cell cycle control, programmed cell death, proteostasis, and intracellular protein transport. Nevertheless, the functional roles of the E1-E2-E3 cascade in insects remains poorly understood.

Within the UPS, E3 ubiquitin ligases, key enzymes in the ubiquitination process, play a pivotal role in selecting the appropriate substrate proteins with high specificity (14). E3s can be categorized into four subfamilies according to their structural characteristics and modes of action: HECT (Homologous to E6-associated protein C Terminus), RING (Really Interesting New Gene), U-Box ligase, and CRL (Cullin-RING Ligase) (15). Among them, RING-finger proteins form a large E3 family with a conserved, cysteine-rich domain (40 to 60 residues) arranged in a specific pattern. This domain contains eight spatially conserved Cys and His residues that bind two zinc atoms. Unlike other DNA-binding zinc-finger domains, the RING-finger domain functions as an E2 binding site, catalyzing the addition of Ub chains to process substrates through an E2–E3–substrate complex (16–18). Previous studies have reported the pivotal role of E3 ligase in regulating stress resistance across diverse species. For instance, E3 ubiquitin ligase FBXO44 regulates pregnane X receptor (PXR) protein level by ubiquitination and degradation in mammalian systems (19). In rice, E3 ligase linked to microtubules targets serine hydroxymethyltransferase for ubiquitination, facilitating its breakdown and triggering a wide-ranging resistance response to viral, fungal, and bacterial pathogens (20). Additionally, E3 ubiquitin ligases RIP1 and APIP6 adjust the Ca^2+^ sensor ROD1, curbing ROS scavenging through the suppression of CatB catalase activity, which results in resistance to various pathogens (21, 22). Moreover, the RING-type E3 ligase EIRP1 degrades VpWRKY11 in the Chinese wild grapevine, thereby positively regulating resistance to powdery mildew (23).

The tripartite motif-containing (TRIM) protein family, a subfamily of the RING E3 ubiquitin ligase family, comprises more than 80 proteins in humans (24). Several studies indicate that TRIM family proteins are crucial in transcriptional regulation, cellular proliferation, metastasis, apoptosis, and tumorigenesis (25–27). For example, TRIM22 promotes diabetic nephropathy by inducing mitochondrial dysfunction via ubiquitination (28). TRIM26 controls the transcriptional factor SOX2 that is involved in tumor growth, treatment resistance, and recurrence (29). TRIM13 suppresses the innate antiviral response by directing antiviral signaling protein toward autophagy-mediated degradation in avian species (30). TRIM25 binds specifically to viral RNA, which is critical for its antiviral activity (31). The UPS is crucial in insects for a wide range of functions, including development, immunity, endoreplication, and cell cycle (32–34). However, the role of components of this system in rapid adaptive traits such as insecticide resistance remains largely unexplored.

*Bemisia tabaci* (Hemiptera: Aleyrodidae) is a notorious pest worldwide that damages a variety of agricultural crops both through direct phloem feeding and by serving as a primary vector for numerous plant viruses (35). Due to the intensive use of insecticides against *B. tabaci*, it has developed resistance to numerous insecticides, including neonicotinoids (36, 37). It has been confirmed that the resistance to neonicotinoids in *B. tabaci* is due to the overexpression of P450s, most notably *CYP6CM1,* resulting in enhanced metabolic detoxification (38, 39). In this context, our recent findings have revealed that *CYP6CM1* is under the *trans*-regulation of the transcription factor cAMP-response element binding protein (CREB) via the mitogen-activated protein kinase (MAPK) signaling pathway (40). Metabolic resistance conferred by P450s is widely assumed to be associated with fitness costs in the absence of insecticide due to the diversion of resources away from developmental processes to metabolic detoxification (41, 42). Related to this, it has been demonstrated that the GPCR-MAPK signaling pathway mediates resistance fitness costs in *B. tabaci* (43, 44). However, the role of ubiquitination in such resistance fitness costs in *B. tabaci* remains uninvestigated.

In the current study, we identify the E1-1/E2-3/TRIM37 enzymatic cascade in *B. tabaci* and investigate its role in insecticide resistance and its associated fitness costs. Our findings reveal a novel resistance mechanism mediated by ubiquitination and elucidate its relationship with a significant fitness cost associated with resistance, providing new insights into the regulation of key resistance genes and the mechanisms mediating fitness trade-offs associated with adaptive traits in insects.

## Results

### Broad-spectrum Resistance to Neonicotinoids in *B. tabaci* MED Is Accompanied by a Reproductive Cost.

**Fig. 1.**
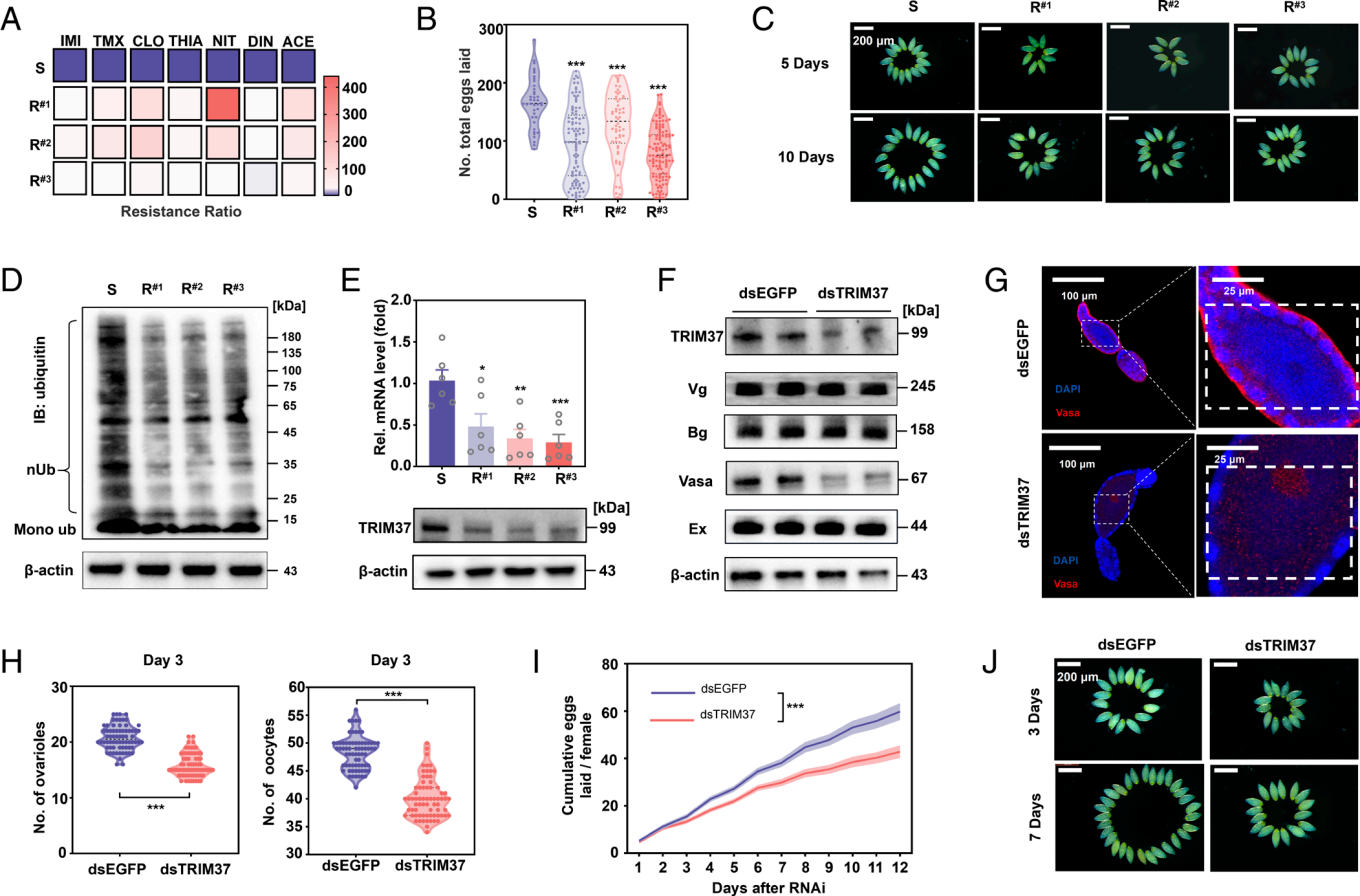
The E3 ubiquitin ligase TRIM37 positively regulates Vasa that confers a reproductive cost associated with neonicotinoid resistance in *B. tabaci* MED. (*A*) Determination of resistance ratio (RR) values in comparisons of neonicotinoid sensitive (S) and resistant whitefly strains (R^#1^, R^#2^, and R^#3^) to seven commonly used neonicotinoids. RRs shown are relative to the reference S strain. Insecticides tested include imidacloprid (IMI), thiamethoxam (TMX), clothianidin (CLO), thiacloprid (THIA), nitenpyram (NIT), dinotefuran (DIN), acetamiprid (ACE). (*B*) Comparison of total egg production in the neonicotinoid sensitive (S) and resistant strains (R^#1^, R^#2^, and R^#3^). (*C*) Representative images showing ovary morphology and ovarian development of the four experimental strains (S, R^#1^, R^#2^, and R^#3^) on day 5 and 10 after eclosion. (Scale bars, 200 μm.) Images were acquired at 100× magnification. (*D*) Western blot analysis was used to detect ubiquitination level of total protein of the four experimental strains. (*E*) Quantification of the mRNA expression and protein level of TRIM37 among the four experimental strains by qPCR and western blot respectively. (*F*) Western blot analysis of the protein expression levels of Vg, Bg, Vasa, and Ex after RNAi knockdown of *TRIM37* in the S strain of *B. tabaci*. (*G*) Immunofluorescence staining assays of Vasa protein in oocytes of female *B. tabaci* ovaries after RNAi knockdown of *TRIM37* in the S strain. Cell nuclei were stained with DAPI (blue). Target protein Vasa was stained with its primary rabbit antibody followed by the secondary antibody Alexa Fluor goat anti-rabbit lgG (red). (Scale bars,100 μm for original images and 25 μm for zoomed-in images.) (*H*) The average number of ovarioles and oocytes per female sampled on day 3 after RNAi knockdown of TRIM37. (*I*) The effect of RNAi knockdown of TRIM37 on female fecundity in the S strain. (*J*) Representative images displaying ovary morphology of female adults sampled on day 3 and 7 after RNAi knockdown of *TRIM37* in the S strain. (Scale bars, 200 μm.) Images were acquired at 100× magnification. All qPCR data are presented as the mean ± SEM of at least three independent experiments, and the resistant strains, R^#1^, R^#2^, and R^#3^, were compared with the S strain. All bioassay data (mean ± SEM) represent at least three biological replicates with approximately 60 adults in each replication. Asterisks indicate significant differences identified by Student’s *t* test (*P < 0.05, **P < 0.01, ***P < 0.001), and absence of asterisks indicates no statistical significance (*P* > 0.05). The susceptible strain S was used in RNAi. Whiteflies fed on dsEGFP served as a negative control in RNAi experiments. *β*-actin was used as a loading control in western blot analysis. All data (mean ± SEM) of ovariole number and oocyte number represent four biological replicates with at least 15 female individuals in each replicate.

Four field populations of *B. tabaci* MED were collected, and their sensitivity to seven neonicotinoids was examined using discriminating dose insecticide bioassays. Three populations (R^#1^, R^#2^, and R^#3^) showed higher survival rates than the susceptible population S (*SI Appendix*, Fig. S1*A*). To more accurately quantify the resistance level in these strains, insecticide bioassays were used to determine the median lethal concentration (LC_50_) and associated resistance ratios (RR_50_) for the seven neonicotinoids. The LC_50_ (*SI Appendix*, Fig. S1*B*) and RR_50_ (Fig. 1*A*) values obtained of the three resistant strains were higher than the susceptible strain S, with the calculated RR_50_ values varying from 1.88- to 439.99-fold. These results confirm that *B. tabaci* MED has developed broad-spectrum resistance to neonicotinoids (*SI Appendix*, Fig. S1*C*).

To identify whether neonicotinoid resistance in *B. tabaci* resistant strains is associated with a fitness cost in the absence of insecticide exposure, we compared life-history traits (*SI Appendix*, Fig. S2) in the four experimental strains. Using the “two sex-age stage” life table approach developed by Chi (45), the resistant strains showed a prolonged developmental time (*SI Appendix*, Fig. S2 *A* and *B* and Table S4), decreased survivorship of immature stages (*SI Appendix*, Fig. S3 *A* and *B*), shortened adult longevity (*SI Appendix*, Fig. S2 *G* and *H*), and life expectancy (*SI Appendix*, Fig. S3*C*), compared with the S strain (*SI Appendix*, Table S5). Furthermore, the preadult time (R^#1^: increased by 21%, *P* = 2.61 × 10^−52^; R^#2^: increased by 13%, *P* = 2.54 × 10^−7^; R^#3^: increased by 18%, *P* = 9 .14 × 10^−47^, *SI Appendix*, Fig. S2*C* and Table S4) and the total oviposition period (TPOP) (R^#1^: increased by 18%, *P* = 2.92 × 10^−23^; R^#2^: increased by 10%, *P* = 2.03 × 10^−4^; R^#3^: increased by 16%, *P* = 2.84 × 10^−34^, *SI Appendix*, Fig. S2*E*) were increased in resistant strains compared with the S strain. The oviposition period (OP) (R^#1^: decreased by 52%, *P* = 2.75 × 10^−17^; R^#2^: decreased by 31%, *P* = 9.50 × 10^−7^; R^#3^: decreased by 55%, *P* = 1.11 × 10^−24^, *SI Appendix*, Fig. S2*F*), longevity (Female: R^#1^: decreased by 51%, *P* = 1.81 × 10^−20^; R^#2^: decreased by 33%, *P* = 2.52 × 10^−8^; R^#3^: decreased by 54%, *P* = 3. 84 × 10^−28^; Male: R^#1^: decreased by 49%, *P* = 4.14 × 10^−12^; R^#2^: decreased by 31%, *P* = 2.78 × 10^−5^; R^#3^: decreased by 39%, *P* = 4.14 × 10^−10^, *SI Appendix*, Fig. S2 *G* and *H*), fecundity (R^#1^: decreased by 41%, *P* = 2.28 × 10^−9^; R^#2^: decreased by 24%, *P* = 2.68 × 10^−54^; R^#3^: decreased by 52%, *P* = 3.99 × 10^−21^, Fig. 1*B* and *SI Appendix*, Fig. S2*I*) and reproductive value (*SI Appendix*, Fig. S3*D*) significantly decreased in the resistant strains relative to the S strain. Further analysis of life-table parameters showed that resistant strains had a lower intrinsic rate of increase (*r*), net reproduction rate (*R*_*0*_), and finite rate (*λ*) than those of the S strain (*SI Appendix*, Table S6). The relative fitness (*Rf*) of R^#1^, R^#2^, R^#3^ was calculated as 0.86, 0.78, 0.88 using the value of intrinsic rate of increase (*r*), and as 0.63, 0.50, 0.64 using the net reproduction rate (*R_0_*) when compared with the S strain (*SI Appendix*, Table S6), revealing a strong reproductive fitness cost associated with resistance. To investigate if the reproductive cost is conferred by female fecundity, we compared ovarian development in the four experimental strains. The number of oocytes in the resistant strains were reduced at each female developmental stage (5 and 10 d) compared with the S strain (Fig. 1*C*). Overall, these data find that a reproductive fitness cost associated with neonicotinoid resistance in *B. tabaci* is mediated, at least in part, by the reduction in oocyte number.

### *TRIM37* Positively Regulates *Vasa* that Confers the Reproductive Cost Associated with Neonicotinoid Resistance in *B. tabaci* MED.

Previous studies have shown that ubiquitination is involved in many physiological and biochemical processes, including resistance to xenobiotics (46, 47). We therefore examined the ubiquitination level of the total protein extracted from the four experimental *B. tabaci* strains by western blot analysis using ubiquitin mouse mAb. The ubiquitination level of the S strain was higher than the resistant strains (Fig. 1*D*). Given this finding, we examined the expression of genes encoding ubiquitin ligases enzymes that are highly flexible and responsible for the recognition of substrates to catalyze the ubiquitination process (10), using RNAseq data of neonicotinoid resistant and susceptible *B. tabaci* strains generated previously (48). This analysis identified an E3 ligase gene *TRIM37* that was differentially expressed between the susceptible and resistant strains. Phylogenetic and protein domain analysis of *TRIM37* provided support for the functional role in ubiquitination (*SI Appendix*, Fig. S4 *B* and *C*). Based on this finding, qRT-PCR was used to investigate the expression of *TRIM37* in the four experimental *B. tabaci* strains. This demonstrated that *TRIM37* was consistently expressed at a lower level in the R^#1^, R^#2^, and R^#3^ strains than in the S strain (R^#1^: decreased by 54%, *P* = 0.019; R^#2^: decreased by 67%, *P* = 1.90 × 10^−3^; R^#3^: decreased by 72%, *P* = 8.50 × 10^−4^, Fig. 1*E*). This finding was corroborated by western blot analysis, which revealed that the protein level of TRIM37 was lower in the resistant strains compared with the susceptible strain, consistent with observed gene expression levels (Fig. 1*E*).

To gain insights into the functional role of TRIM37, we used RNAi to knock down the expression of *TRIM37* (decreased by 61%, *P* = 2.02 × 10^−9^; *SI Appendix*, Fig. S5*A*) in the S strain and examined its effect on reproductive fitness. Interestingly, we observed a decrease in the number of ovarioles, oocytes on day 3 (ovarioles: decreased by 23%, *P* = 9.29 × 10^−27^; oocytes: decreased by 17%, *P* = 1.02 × 10^−30^, Fig. 1*H*) and day 7 (ovarioles: decreased by 23%, *P* = 3.37 × 10^−28^; oocytes: decreased by 21%, *P* = 2.18 × 10^−36^, *SI Appendix*, Fig. S5*C*), and female fecundity (decreased by 29%, *P* = 9.84 × 10^−5^, Fig. 1*I* and *SI Appendix*, Fig. S5*B*) compared with the dsEGFP control, leading to a decline in ovary growth (Fig. 1*J*). These observations provide strong evidence of the role of this gene in regulating female fecundity.

Oogenesis involves the coordinated action of numerous genes (49, 50). Among them, *Vg*, *Bg*, *Vasa*, and *Ex* have been shown to be key genes in this process in *B. tabaci* (44). Thus, we used RNAi to knockdown *TRIM37* in the S strain and examined its effect on the protein level of these four key oogenesis genes. After knockdown of *TRIM37*, the protein level of Vasa decreased, whereas no obvious changes were observed in the protein levels of the other three genes (Fig. 1*F*), compared with the control dsEGFP. This result was also corroborated by immunofluorescence (IF) staining (Fig. 1*G* and *SI Appendix*, Fig. S6).

### The Key Oogenesis Gene *Vasa* Underlies the Reproductive Cost.

To further verify the role of *Vasa* in the experimental strains, qRT-PCR was used to assess its expression in the four experimental strains. These results revealed that *Vasa* is expressed at lower levels in the resistant strains than in the S strain (R^#1^: decreased by 55%, *P* = 1.97 × 10^−6^; R^#2^: decreased by 60%, *P* = 7.51 × 10^−6^; R^#3^: decreased by 65%, *P* = 9.83 × 10^−6^, *SI Appendix*, Fig. S7*A*). To confirm that this translated to altered expression of the encoded enzyme, we used western blot analysis to reveal that the Vasa protein was reduced in the resistant strains compared with the S strain (*SI Appendix*, Fig. S7*A*). IF staining assay also showed a lower protein signal for Vasa in the oocytes in resistant strains compared with the S strain (*SI Appendix*, Fig. S7*B*). These findings corroborate the link between the downregulation of *Vasa* at specific stages of oogenesis and the observed reproductive cost.

To further validate the role of *Vasa* in this reproductive cost, RNAi knockdown of *Vasa* in the S strain was performed and its effect on ovarian development and female fecundity was examined. Knock down of *Vasa* (decreased by 63%, *P* = 4.02 × 10^−5^, *SI Appendix*, Fig. S7*C*) reduced its protein level by western blot analysis (*SI Appendix*, Fig. S7*C*) and decreased its signal in oocytes in IF staining analysis (*SI Appendix*, Fig. S7*D*) compared with the dsEGFP control. Silencing of *Vasa* leads to a reduction in female total egg production (decreased by 29%, *P* = 9.03 × 10^−5^, *SI Appendix*, Fig. S7 *E* and *F*), ovariole number (day 3: decreased by 15%, *P* = 3.07 × 10^−13^; day 7: decreased by 17%, *P* = 1.41 × 10^−13^, *SI Appendix*, Fig. S7*G*), and oocyte number (day 3: decreased by 13%, *P* = 2.45 × 10^−18^; day 7: decreased by 16%, *P* = 1.40 × 10^−19^, *SI Appendix*, Fig. S7*H*), relative to the dsEGFP control, which was associated with an abnormal ovarian development phenotype (*SI Appendix*, Fig. S7*I*).

### The E1-1/E2-3/TRIM37 Ubiquitination Cascade Regulates *Vasa*.

**Fig. 2.**
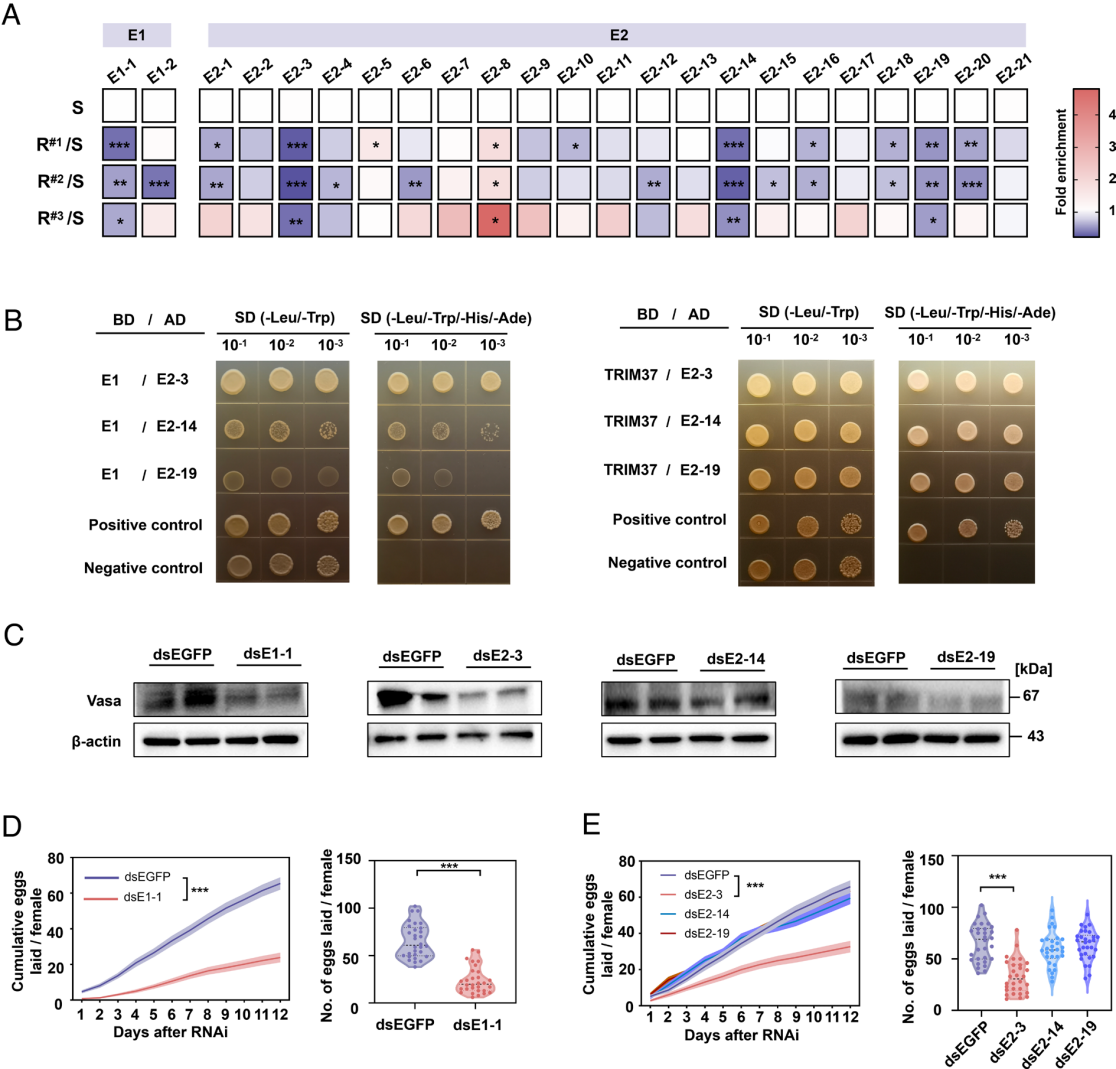
The E1-1/E2-3/TRIM37 cascade positively regulates *Vasa*. (*A*) Quantification of the expression of genes involved in the ubiquitin-activating enzyme E1s and ubiquitin-conjugating E2s among the experimental strains. (*B*) Y2H assays showing the interaction between E1 and E2s, E2s and TRIM37 in vitro. BD, bait vector (DNA-binding domain fusion). AD, prey vector (activation domain fusion). SD, yeast drop-out culture medium. pGBKT7-53 and pGADT7-T were used as a positive control. pGBKT7-lam and pGADT7-T were used as a negative control. (*C*) Western blot analysis of the expression of Vasa after RNAi knockdown of three E2s in the S strain. (*D* and *E*) The effect after RNAi knockdown of *E1-1* and three *E2s* on female fecundity. All qPCR data are presented as the mean ± SEM of at least three independent experiments, and the resistance strains, R^#1^, R^#2^, and R^#3^, were compared with the S strain. All bioassay data (mean ± SEM) represent at least three biological replicates with approximately 60 adults in each replication. Asterisks indicate significant differences as determined by Student’s *t* test (*P < 0.05, **P < 0.01, ***P < 0.001), and absence of asterisks indicates no statistical significance (*P* > 0.05). The susceptible strain S was used in RNAi. Whiteflies fed on dsEGFP served as a negative control in RNAi experiments. *β*-actin was used as a loading control in western blot analysis. All data (mean ± SEM) on ovariole number and oocyte number represent four biological replicates with at least 15 female individuals in each replicate.

Since TRIM37 acts as an E3 ligase that positively regulates Vasa (Fig. 1*F*), and given that E1 and E2 enzymes are essential upstream components of the ubiquitination cascade, we next examined the expression of all annotated E1 and E2 genes in the whitefly genome to identify those that may function together with TRIM37. Based on *B. tabaci* genomic data (https://www.whiteflygenomics.org/cgi-bin/bta/index.cgi), we selected two E1 and 21 E2 genes (*SI Appendix,* Table S8) and examined their expressions in the experimental strains by qPCR analysis. E1-1 and three of the 21 E2s were found to be down-regulated in the resistant strain compared with the S strain (Fig. 2*A*). To gain insights into the binding mechanism of E1-1/E2/TRIM37, we performed docking studies of these selected proteins and modeled the binding complex of E1-1 with E2s and E2s with TRIM37. The results suggested that E1-1 interacts with three E2s, and the three E2s interact with *TRIM37* (*SI Appendix*, Fig. S8 *A*–*F* and Table S13). To further confirm the interaction between E1 and E2, E2, and E3 in vitro, yeast two-hybrid assays were performed revealing that E1-1 and E3 ligase TRIM37 could interact with the three E2s directly (Fig. 2*B*). After knockdown of the four ubiquitin genes, respectively, the protein levels of Vasa were all decreased, compared with the control dsEGFP (Fig. 2C). To further validate the relationship between the cascade and the observed fitness cost associate with insecticide resistance, RNAi knockdown of E1-1 and three E2s in the S strain were performed and their effects on ovarian development and female fecundity was examined. Silencing of E1-1 (decreased by 63%, *P* = 2.87 × 10^−14^, Fig. 2*D*) and E2-3 (decreased by 50%, *P* = 6.20 × 10^−10^) also decreased female reproduction (Fig. 2*E*). To understand why only E2-3, but not E2-14 or E2-19, affected fecundity, we examined their expression levels in ovarian tissue. As shown in *SI Appendix*, Fig. S9, E2-3 was expressed at significantly higher levels in the ovary compared to E2-14 and E2-19 (*P* < 0.05). This tissue-specific expression explains the functional predominance of E2-3 in regulating reproductive phenotypes. These results provide strong evidence that the E1-1/E2-3/TRIM37 cascade regulates *Vasa*, thereby mediating the observed reproductive cost.

### TRIM37 Does Not Directly Interact with Vasa.

**Fig. 3.**
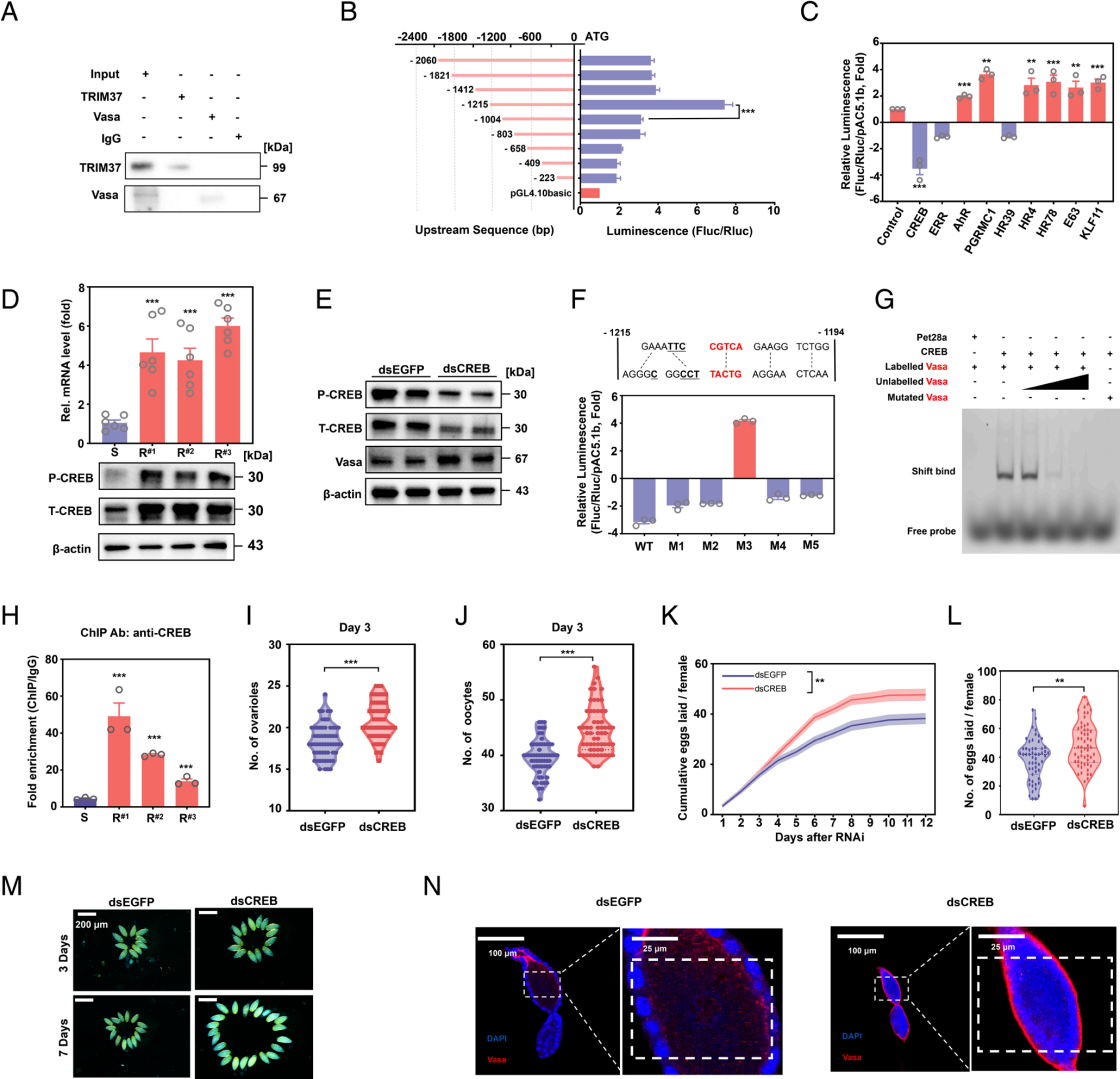
The transcription factor CREB negatively regulates Vasa expression. (*A*) IP analysis of the direct interaction of TRIM37 with Vasa protein in the S strain. Extracted total protein from whiteflies was incubated with an antibody for TRIM37 or Vasa followed by incubation with protein A/G-magnetic beads. Then the TRIM37 and Vasa proteins were detected by their corresponding antibodies in western blots. Input and IgG were used as a positive and negative control. (*B*) Transcriptional activity of putative promoter sequences upstream of *Vasa* in dual luciferase reporter gene assays. S2 cells were cotransfected with pGL4.10 plasmids carrying the indicated promoter regions and a reference reporter plasmid pGL4.73 (n = 3, mean ± SE). (*C*) Effects of 9 transcription factors (TFs) on the activity of the *Vasa* promoters in dual luciferase reporter gene assays. pGL4.10-Va ^−1,215 to +1^ and pGL4.73 were cotransfected into S2 cells with constructs containing each TFs in the pAc5.1b vector, and the empty pAc5.1b was used as a control (n = 3, mean ± SE). (*D*) Determination of *CREB* gene expression and CREB protein level (T-CREB: the total of protein CREB, P-CREB: the phosphorylated form of CREB) in the experimental strains (S, R^#1^, R^#2^, and R^#3^) by qRT-PCR and western blots analysis. (*E*) Effects of RNAi knockdown of *CREB* on the protein level of Vasa in R^#1^. (*F*) The impact of CREB on wild-type or mutated promoters of *Vasa* covering putative CREB binding sites. A series of mutant constructs containing 5 base pair mutations in each of the identified binding sequences were cotransfected into S2 cells with pGL4.73 and pAC5.1b-CREB. Empty pAC5.1b was used as an internal control. (*G*) Electrophoretic mobility shift assay (EMSA) of the binding of purified CREB protein to the putative promoter sequences of *Vasa* in vitro. Details for all oligonucleotide probes in the EMSA assays are summarized in *SI Appendix*, Table S11. (*H*) ChIP-qPCR analysis of the binding of CREB protein to the putative promoter sequences of *Vasa* in the resistance strains, R^#1^, R^#2^, and R^#3^, compared with the S strain. Note that the core promoter region covering the putative binding site was established following IP of the chromatin fragment with anti-CREB. (*I* and *J*) Average number of ovarioles (*I*) and oocytes (*J*) per female whitefly sampled on day 3 after RNAi knockdown of *CREB*. (*K* and *L*) The effect after RNAi knockdown of *CREB* on female fecundity. (*M*) Representative images displaying ovary morphology of female whitefly adults sampled on day 3 and 7 after RNAi knockdown of *CREB*. (Scale bars, 200 μm.) Images were acquired at 100× magnification. (*N*) Immunofluorescence staining assays of Vasa protein locating in oocytes of female ovaries after RNAi knockdown of CREB. Cell nuclei were stained with DAPI (blue). Target protein Vasa was stained with their primary rabbit antibodies followed by the secondary antibody Alexa Fluor goat anti-rabbit lgG (red). (Scale bars, 100 μm for original images and 25 μm for zoom-in images.) All qPCR data are presented as the mean ± SEM of at least three independent experiments, and the resistance strains, R^#1^, R^#2^, and R^#3^, were compared with the S strain. All bioassay data (mean ± SEM) represent at least three biological replicates with approximately 60 adults in each replication. Asterisks indicate significantly differences as determined by Student’s *t* test (*P < 0.05, **P < 0.01, ***P < 0.001), and absence of asterisks indicates no statistical significance (*P* > 0.05). The resistant strain R^#1^ was used in RNAi experiments. Whiteflies fed on dsEGFP served as a negative control in RNAi experiments. All data (mean ± SEM) on ovariole number and oocyte number represent four biological replicates with at least 15 female individuals in each replication.

To elucidate if TRIM37 ubiquitinates Vasa directly in vivo, immunoprecipitation (IP) was performed to investigate the interaction of TRIM37 with Vasa in the S strain of *B. tabaci*. Western blot analysis showed that TRIM37 was not detected after whitefly total protein was incubated with antibodies for Vasa in IP assays, and vice versa (Fig. 3*A*), suggesting that the two proteins do not interact directly.

### *Vasa* Promoter Analysis.

To investigate the transcriptional regulation of *Vasa*, a series of constructs comprising nine different regions upstream of the gene (−2,060 to +1, −1,821 to +1, −1,412 to +1, −1,215 to +1, −1,004 to +1, −803 to +1, −658 to +1, −409 to +1, −223 to +1) were created, and the ability of each region to drive expression of a reporter gene was assessed using dual luciferase reporter assays. The construct containing the region −1,215 to +1 (pGL4.10-Vasa^−1,215 to +1^) showed the most pronounced expression of the reporter, suggesting that the promoter is located in this region, with particularly important elements located between −1,215 and −1,004 bp (Fig. 3*B*).

### The Transcription Factor CREB Regulates *Vasa* Expression.

Based on an extensive literature search, we selected nine transcription factors with potential roles in insect development, reproduction, and xenobiotic stress response from the recently sequenced genome of *B. tabaci* (*SI Appendix,* Table S8). To explore if any of these regulate *Vasa* expression, the nine transcription factors were individually cloned into the pAC5.1b expression vector and cotransfected into S2 cells with pGL4.10-Vasa^−1,215 to +1^. In dual luciferase reporter assays, only S2 cells overexpressing the CREB protein demonstrated a decline in the transcriptional activity of *Vasa* (*P* = 5.48 × 10^−4^, Fig. 3*C*), suggesting that CREB negatively regulates *Vasa*.

The deduced amino acid sequence of CREB protein in *B. tabaci* contains important conserved domains, which is common to the bZIP family of transcription factors, including the basic leucine zipper DNA-binding, dimerization domain (bZIP) and a central kinase-inducible domain (KID) that interacts with protein kinases (40). To elucidate the importance of the two domains of CREB in inhibiting the promoter of *Vasa*, we created a CREB-wild type (WT) construct and two mutant CREB constructs, CREB-DN1, which lacks the KID domain, and CREB-DN2, which lacks the bZIP domain. These constructs were then cotransfected into S2 cells with the pGL4.10-Vasa −1,215 to +1 construct. Compared with the WT control, coexpression with the two mutant constructs was associated with an increase in the transcriptional activity of the promoters of *Vasa* (*SI Appendix,* Fig. S10*A*), revealing the importance of the two domains of CREB in the transcriptional regulation of *Vasa*.

To investigate the role of CREB in vivo, qPCR and western blot analyses were conducted to quantify CREB expression in the experimental strains. The mRNA levels (R^#1^: *P* = 4.01 × 10^−4^; R^#2^: *P* = 4.73 × 10^−4^; R^#3^: *P* = 5.86 × 10^−4^, Fig. 3*D*), total protein levels, and the phosphorylated form of CREB (Fig. 3*D*) were found to be elevated in the resistant strains compared with the S strains. To validate the functional role of CREB in regulating *Vasa* in vivo, RNAi was used to knockdown CREB in the R^#1^ strain (decreased by 73%, *P* = 3.03 × 10^−5^, *SI Appendix,* Fig. S10*B*), then its effect on gene expression and protein levels of *Vasa* were examined. Knockdown of CREB significantly enhanced expression of *Vasa* (*P* = 2.59 × 10^−4^) compared with the control dsEGFP (*SI Appendix,* Fig. S10*C*). This result was also corroborated by western blot (Fig. 3*E*) and IF staining analysis (Fig. 3*N* and *SI Appendix,* Fig. S11), which showed an increased protein level of Vasa after knockdown of CREB. Collectively, these results confirmed that CREB is a transcriptional repressor of *Vasa*.

To identify putative CREB binding sites in the core promoter of its target gene, the candidate binding sequence (CCTCGTCA) was identified in the *Vasa* promoter through the JASPAR database (*SI Appendix*, Fig. S10*D* and Table S9). Given that CREB stimulates target gene expression at the half-site-like sequence CGTCA (40), a series of reporter gene constructs were created, containing the *Vasa* promoter with the binding sequences (WT) and another set in which these sites were mutated (M1, M2, M3, M4, M5). Among them, M3 (CGTCA) showed the highest transcriptional activity compared with the WT control, suggesting that this mutated region (CGTCA) contains a CREB binding site (Fig. 3*F*). EMSA were then used to identify the protein-nucleic acid interactions of purified CREB protein to a DNA probe of the identified sequence in vitro. The binding of CREB protein to the DNA probe was confirmed as a shifted band, but the band disappeared when an unlabeled competitor DNA probe or a mutated probe was used (Fig. 3*G*). ChIP-qPCR assays were then conducted to demonstrate direct binding of CREB to the core promoter region of *Vasa* in vivo. The result showed that the relative expression of CREB-DNA fragments was higher in the resistant strains than the S strain (R^#1^: *P* = 3.41 × 10^−3^; R^#2^: *P* = 3.14 × 10^−6^; R^#3^: *P* = 1.92 × 10^−3^, Fig. 3*H*), revealing a greater level of binding of CREB to the promoter sequences in the resistant strains. Taken together, these findings provide strong evidence that CREB reduces *Vasa* expression as a result of its binding to a site in the upstream promoter of this gene.

To investigate the potential role of CREB in the reproductive cost associated with neonicotinoid resistance, RNAi was used to knockdown the expression of *CREB*. After silencing of CREB, the number of ovarioles and oocytes on day 3 (ovarioles: increased by 13%, *P* = 4.57 × 10^−9^; oocytes: increased by 13%, *P* = 1.00 × 10^−10^, Fig. 3 *I* and *J*), day 7 (ovarioles: increased by 11%, *P* = 2.43 × 10^−7^; oocytes: increased by 12%, *P* = 5.55 × 10^−8^, *SI Appendix,* Fig. S10*E*) and fecundity (increased by 25%, *P* = 1.28 × 10^−3^, Fig. 3 *K* and *L*) were increased relative to the dsEGFP control, leading to enhanced growth of whitefly ovaries (Fig. 3*M*). Collectively, these findings imply that CREB-mediated regulation of *Vasa* leads to a reproductive fitness cost. Having established that CREB represses *Vasa*, we next asked how CREB itself is regulated. Given that TRIM37 is downregulated in resistant strains and its knockdown affects Vasa, we hypothesized that TRIM37 might directly target CREB for ubiquitination and degradation.

### TRIM37 Mediates CREB Ubiquitination and Degradation.

**Fig. 4.**
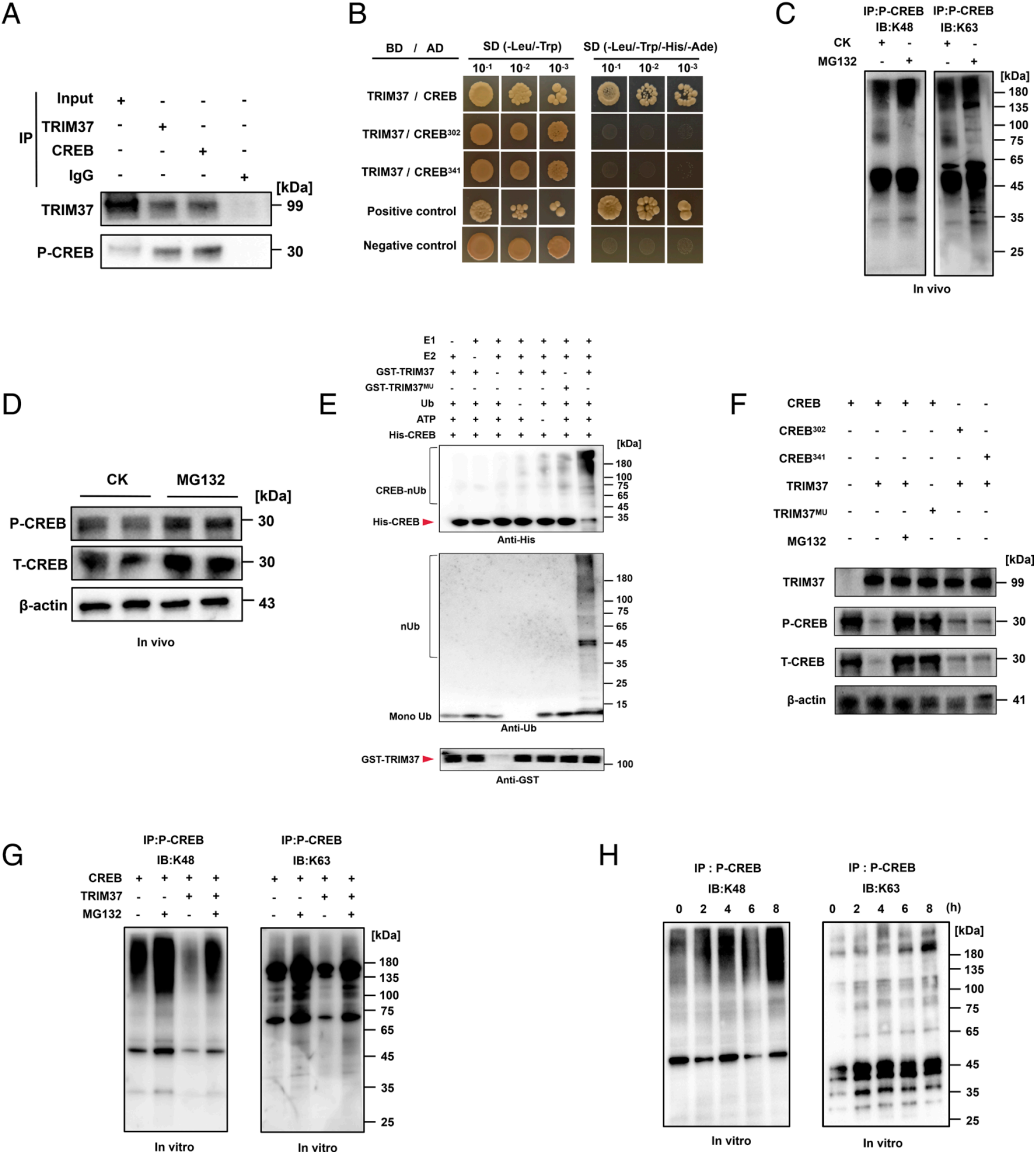
TRIM37 ubiquitinates the transcription factor CREB. (*A*) IP analysis of the interaction of TRIM37 with CREB protein in *B. tabaci* (S strain). Extracted protein was incubated with antibodies for TRIM37 and CREB. Then the TRIM37 and CREB proteins were detected by their corresponding antibodies in western blots. Input and IgG were used as a positive and negative control. (*B*) Y2H assays showing the interaction between TRIM37 and CREB in vitro. CREB^302^ contains a mutation abolishing the K48 ubiquitination site, CREB^341^ contains a mutation abolishing the K63 ubiquitination site. BD, bait vector (DNA-binding domain fusion). AD, prey vector (activation domain fusion). SD, yeast drop-out culture medium. pGBKT7-53 and pGADT7-T were used as a positive control. pGBKT7-lam and pGADT7-T were used as a negative control. (*C*) The degradation of CREB via the 26S proteasome pathway in *B. tabaci* MED. Total protein was extracted from *B. tabaci* fed with the 26S proteasome inhibitor MG132 (50 μM) for 24 h, with DMSO used as a control. Then the protein was immunoprecipitated with anti-CREB and detected by anti-K48/anti-K63 in western blots. (*D*) The effect of CREB protein level in vivo after feeding *B. tabaci* with MG132 (50 μM). DMSO was used as a control. CREB protein was then detected using anti-CREB in western blots. (*E*) In vitro ubiquitination assay of CREB by TRIM37. Western blot analysis was performed using anti-His, anti-Ub, or anti-GST antibody. CREB-nUb denotes the ubiquitinated CREB band. nUb denotes the ubiquitinated chain band. Mono Ub denotes free ubiquitin. Note that GST-TRIM37 means wild-type, GST-TRIM37^MU^ means RING mutant (C25S and C28S): two cysteine residues C25S and C28S, both mutated to serine. His-tagged CREB was expressed from pET28a vector. GST-tagged TRIM37 (wild-type) and GST-tagged TRIM37^MU^ (RING mutant, C25S/C28S) were expressed from pGEX-4T-1 vector. (*F*) TRIM37 promotes the degradation of CREB in vitro. pAC5.1b-based constructs were used for expression of CREB (wild-type; K48 mutant: CREB^302^; K63 mutant: CREB^341^) and TRIM37 (wild-type or RING mutant TRIM37^MU^: C25S/C28S). Note that pAC5.1b-CREB^302^ contains a mutation that abolishes the K48 ubiquitination site, pAC5.1b-CREB^341^ contains a mutation abolishing the K63 ubiquitination site, TRIM37^MU^ refers to the two cysteine residues C25S and C28S, both mutated to serine. S2 cells were cotransfected as indicated, with or without MG132 (50 μM) treatment. Whole-cell lysates were analyzed by western blot using anti-CREB (T-CREB: the total of protein CREB, P-CREB: the phosphorylated form of CREB), anti-TRIM37, and anti-*β*-actin antibodies. Anti-*β*-actin served as loading control. (*G*) TRIM37 promotes the degradation of CREB via the 26S proteasome pathway in vitro. PAC-TRIM37 and pAC5.1b-CREB were coexpressed in S2 cells, MG132 (50 μM) was infiltrated into the S2 cells 24 h before sampling. DMSO was used as a control. (*H*) CREB ubiquitination level after exposure of S2 cells expressing pAC5.1b-TRIM37 and pAC5.1b-CREB to MG132. Total protein was extracted from the S2 cells after treatment with MG132 (50 μM) for 0 to 8 h. Then the protein was immunoprecipitated with anti-CREB and detected by anti-K48/anti-K63 in western blots.

TRIM37 is a predicted E3 ligase that contains a RING domain essential for E3 activity. Furthermore, we previously demonstrated that TRIM37 was downregulated in a neonicotinoid resistant strain of *B. tabaci* by transcriptome profiling (48). To further predict the binding interface between TRIM37 and CREB, we performed molecular docking analysis. The predicted three-dimensional structure shows that TRIM37 interacts with CREB through multiple residues, including hydrogen bonds and salt bridges (*SI Appendix*, Fig. S8*G* and Table S13). In the light of this, we used the Co-IP assay to determine whether CREB is a substrate of TRIM37. We observed that TRIM37 and CREB were detected after whitefly total protein was incubated with antibodies for either protein after the IP assay (Fig. 4*A*), suggesting a direct interaction between them in vivo. Given that TRIM37 may ubiquitinate CREB directly, we predicted two ubiquitination sites of CREB, including the amino acid position 302 containing the K48 ubiquitination site and the amino acid position 341 containing the K63 ubiquitination site. The wild type vectors TRIM37 and CREB, the mutation vectors CREB^302^ and CREB^341^ (K48 mutation site and K63 mutation, respectively), were then generated to further confirm the interaction between TRIM37 and CREB in vitro using yeast two-hybrid assays. In these assays, TRIM37 was found to interact with CREB strongly whereas the mutant form of CREB did not (Fig. 4*B*), indicating that TRIM37 may ubiquitinate CREB by its action at the K48 and K63 sites.

To determine whether TRIM37-mediated CREB ubiquitination triggers CREB degradation in vivo, CREB protein was recovered from adult *B. tabaci* after treatment with 50 μM MG132 (an inhibitor of proteasomes) by IP assay. Western blot assays were then performed to detect CREB using linkage-specific K48 ubiquitin antibody and linkage-specific K63 ubiquitin antibody. We observed an increase in CREB abundance in the presence of MG132 compared with the DMSO control (Fig. 4*C*). The total protein levels and the phosphorylated form of CREB were also higher after the MG132 treatment compared with the DMSO treatment (Fig. 4*D*), which is consistent with the results of K48/K63 western blot assays. These results indicate that CREB is polyubiquitinated by TRIM37 at the K48 and K63 sites in vivo and is degraded by the 26S proteasome.

To further determine whether CREB is a substrate of TRIM37, we performed an in vitro ubiquitination assay. We observed the specific high-molecular-weight ubiquitinated band of His-CREB when the recombinant protein was incubated with GST-TRIM37 in the presence of E1 ubiquitin-activating enzyme (E1), E2 ubiquitin-conjugating enzyme (E2), ubiquitin (Ub), and ATP. When any of these proteins were omitted from the reaction, the ubiquitinated form of His-CREB was not detectable (Fig. 4*E*). Importantly, a RING-domain inactive mutant of TRIM37 (GST-TRIM37^MU^, C25S/C28S) completely failed to ubiquitinate CREB (Fig. 4*E*), demonstrating that the E3 ligase activity of TRIM37 is strictly required.

Moreover, we coexpressed CREB with TRIM37 in S2 cells. As shown in Fig. 4*F*, wild-type TRIM37 markedly reduced CREB protein levels compared to CREB alone (lane 1 vs. lane 2). Addition of the proteasome inhibitor MG132 completely restored CREB levels (lane 3), indicating that TRIM37 targets CREB for proteasomal degradation in vitro. To assess the involvement of specific ubiquitination sites, we generated two single-point mutants: CREB^302^ (K48) and CREB^341^ (K63). When coexpressed with TRIM37, each single mutant partially attenuated the reduction of CREB protein levels compared to wild-type CREB (lanes 5 and 6 vs. lane 2). These results suggest that both K48 and K63 contribute to TRIM37-mediated ubiquitination, but neither is absolutely required. Importantly, a RING-domain inactive mutant of TRIM37 (C25S/C28S) completely restored CREB levels (lane 4), confirming that the E3 ligase activity of TRIM37 is essential for CREB degradation.

The protein level of CREB was also reduced in the presence of TRIM37 when detected using K48/K63 ubiquitin antibody, while it was increased after treatment with 50 μM MG132 (Fig. 4*G*). To further determine the effect of 26S proteasome degradation, which targets and degrades proteins marked with ubiquitin, in vitro TRIM37 and CREB were cotransfected into S2 cells. We observed a gradual increase in CREB abundance over time, in the presence of 50 μM MG132, detected by K48/K63 ubiquitin antibodies, implying that CREB was degraded by the 26S proteasome (Fig. 4*H*), which is consistent with the steady-state increases observed in whiteflies (Fig. 4 *C* and *D*) and S2 cells (Fig. 4 *F* and *G*) at single time points. Taken together, these findings reveal that TRIM37 targets CREB for ubiquitination in *B. tabaci*. To further validate that TRIM37-mediated CREB degradation relieves CREB-dependent transcriptional repression, we overexpressed TRIM37 in *Drosophila* S2 cells and examined *Vasa* mRNA levels. As shown in *SI Appendix*, Fig. S12*A*, TRIM37 overexpression significantly increased *Vasa* mRNA (*P* = 3.25 × 10^−5^), consistent with the expected derepression upon CREB loss. This result directly supports the model that TRIM37 acts upstream of CREB to positively regulate Vasa expression (*SI Appendix,* Fig. S12*B*).

### The E1-1/E2-3/TRIM37 Cascade Negatively Regulates *CYP6CM1* Expression Conferring Neonicotinoid Resistance.

**Fig. 5.**
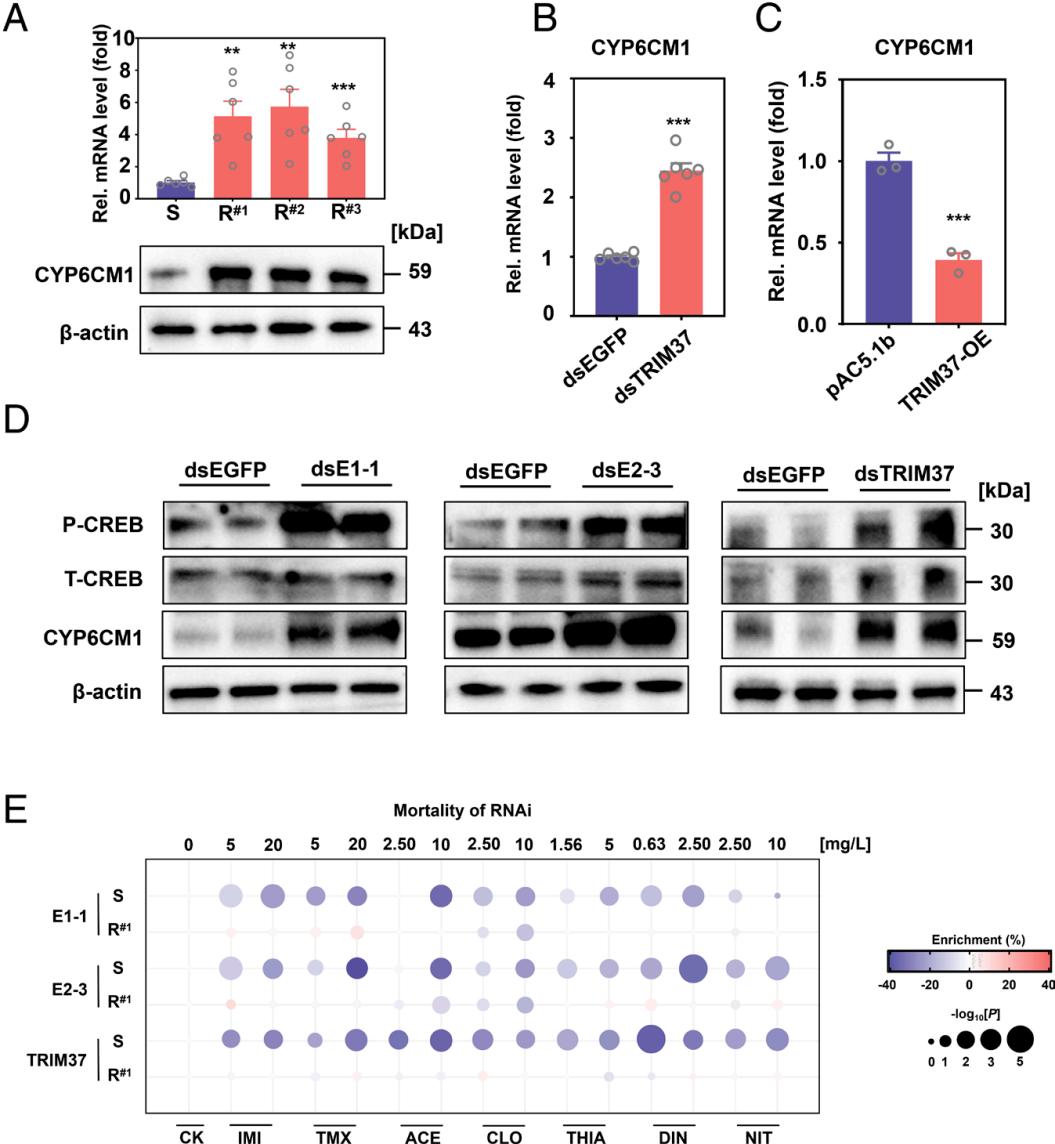
TRIM37 negatively regulates *CYP6CM1* that mediates broad-spectrum resistance to neonicotinoids in *B. tabaci* MED. (*A*) Quantification of the mRNA expression and western blot analysis of proteins level of CYP6CM1 among the experimental strains of *B. tabaci* (S, R^#1^, R^#2^, and R^#3^). (*B*) Effects after RNAi knockdown of *TRIM37* on the expression of *CYP6CM1*. (*C*) Effect of TRIM37 overexpression on *CYP6CM1* mRNA levels in S2 cells. *Drosophila* S2 cells were transfected with either empty pAC5.1b vector (control) or pAC5.1b-TRIM37 overexpression construct (TRIM37-OE). After 48 h, the relative mRNA level of *CYP6CM1* was measured by qRT-PCR. (*D*) Effect of RNAi knockdown of *E1-1/E2-3/TRIM37* on the protein level of CREB and CYP6CM1. (*E*) Sensitivity of experimental strains of *B. tabaci* to seven neonicotinoids after RNAi knockdown of *E1-1/E2-3/TRIM37*. Mortality was assessed 48 h after insecticide first exposure. All qPCR data are presented as the mean ± SEM of at least three independent experiments. All bioassay data (mean ± SEM) represent at least three biological replicates with approximately 60 adults in each replication. Asterisks indicate significant differences as determined by Student’s *t* test (*P < 0.05, **P < 0.01, ***P < 0.001), and absence of asterisks indicates no statistical significance (*P* > 0.05). The susceptible strain S was used in RNAi experiments. Whiteflies fed on dsEGFP served as a negative control in RNAi experiments. *β*-actin was used as a loading control in western blot analysis.

CREB has been previously shown to play a key role in regulating the expression of the P450 gene *CYP6CM1*, leading to neonicotinoid resistance in *B. tabaci* (40). Given this and our findings above, we explored if E1-1/E2-3/TRIM37-mediated CREB ubiquitination plays a role in neonicotinoid resistance by regulating *CYP6CM1* expression in the four experimental strains. qRT-PCR and western blot assays were therefore used to assess the mRNA expression level and the protein level of CYP6CM1 in the four strains. The results revealed a higher expression of this P450 gene/protein in the resistance strains (R^#1^: *P* = 1.31 × 10^−3^; R^#2^: *P* = 1.39 × 10^−3^; R^#3^: 4.74 × 10^−4^, Fig. 5*A*). We further conducted RNAi knockdown of *E1-1*, *E2-3,* or *TRIM37* to evaluate their roles in neonicotinoid resistance (*P* = 5.04 × 10^−7^, Fig. 5*B*). Consistently, TRIM37 overexpression in S2 cells significantly reduced *CYP6CM1* mRNA levels (Fig. 5*C*, *P* = 7.21 × 10^−4^), further confirming that TRIM37 negatively regulates CYP6CM1 via CREB. Silencing of *E1-1*, *E2-3,* or *TRIM37* led to an increase in the protein levels of CREB and CYP6CM1 (Fig. 5*D*). Subsequent insecticide bioassays of the susceptible strain (S) and resistance strain (R^#1^) revealed a decreased sensitivity of susceptible *B. tabaci* MED to seven neonicotinoids after RNAi knockdown of *E1-1*, *E2-3,* or *TRIM37* (Fig. 5*E*). Thus, we conclude that the E1-1/E2-3/TRIM37 cascade plays an important role in the resistance of *B. tabaci* MED to neonicotinoids via ubiquitination of CREB which, in turn, regulates *CYP6CM1* expression.

## Discussion

**Fig. 6.**
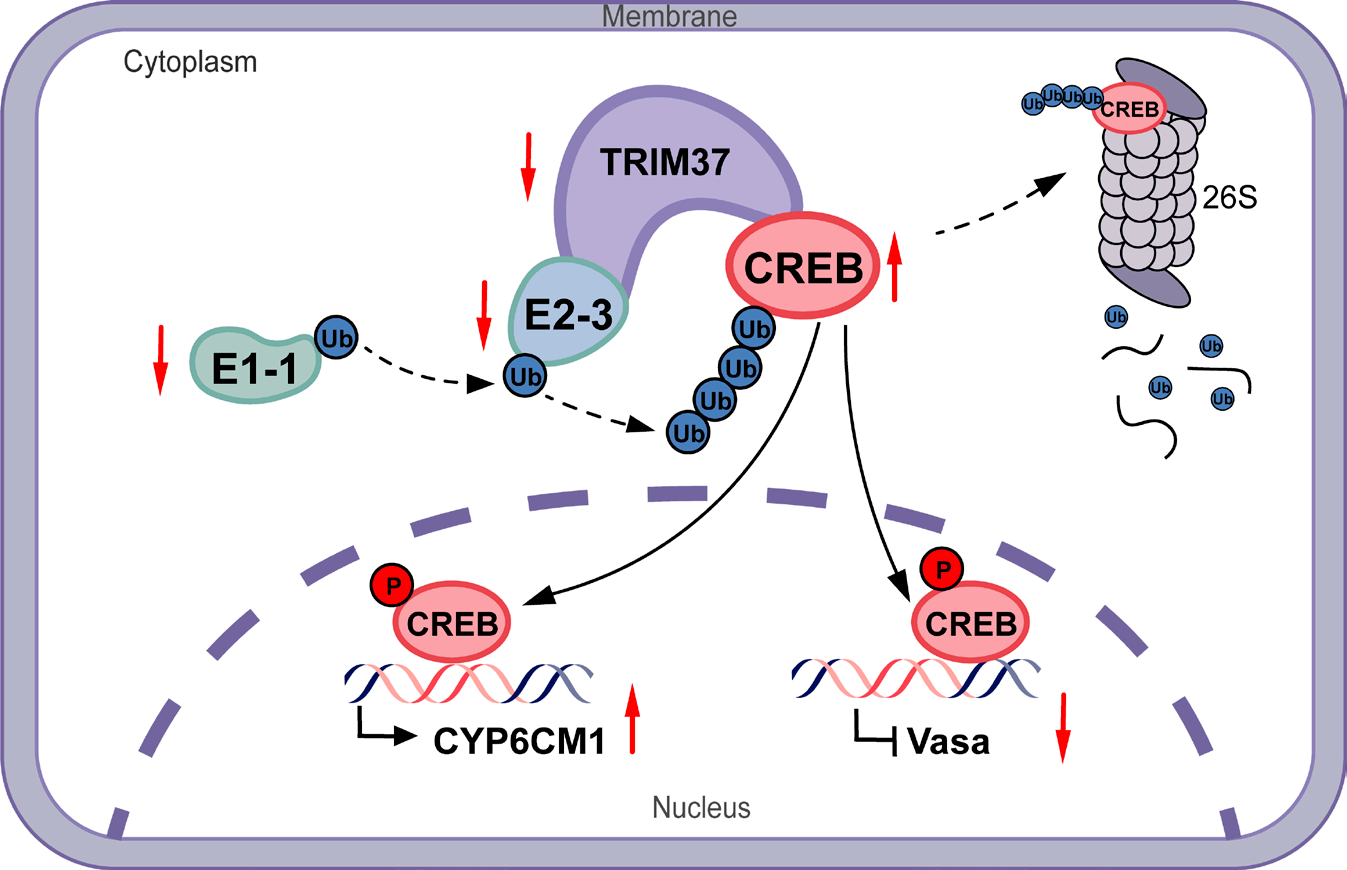
Schematic of the E1-1/E2-3/TRIM37/CREB cascade underlying trade-offs between insecticide resistance and reproductive cost in *B. tabaci* MED. The E1-1/E2-3/TRIM37 cascade ubiquitinates and suppresses the activity of the transcription factor CREB. CREB contributes to overexpression of *CYP6CM1* that confers resistance to neonicotinoids, while negatively regulating *Vasa*, leading to abnormal ovary growth and the reproductive cost associated with resistance.

Our findings reveal the role of the E1-1/E2-3/TRIM37 ubiquitination cascade in the regulation of a key transcription factor in *B. tabaci*, leading to insecticide resistance and its associated fitness cost (Fig. 6). These results advance understanding of the insect ubiquitination system and the role of posttranslational modifications in regulating key genes involved in resistance and reproduction, providing novel targets for future pest control.

In this study, we identify a ubiquitin cascade comprising ubiquitin-activating enzyme E1-1, ubiquitin-conjugating enzyme E2-3, and ubiquitin-protein ligase TRIM37, which regulates the transcription factor CREB. Most cellular ubiquitination processes involve the E1-E2-E3 cascade. Abnormal E1-E2-E3 cascade activity or ubiquitination causes or drives many human diseases, cell survival, and plant adaptive immunity (47, 51). In insects, ubiquitin cascades are known to play a role in many physiological and biochemical functions. Mutation in E1 reduces *Drosophila* lifespan and results in motor impairment (52). Knockdown of E1 and E2 triggers defective chorion biogenesis and modulation of autophagy-related genes in the follicle cells of the vector *Rhodnius prolixus* (53). In *Bactrocera dorsalis*, E1 enzyme Uba1 impairs male fertility (54). However, very little is known about the role of ubiquitination in regulating specific transcription factors of insects. In plants, E3 ubiquitin ligases can play a key role in regulating bZIP transcription factors by influencing their stability (55). For example, in Arabidopsis, the RING-type E3 ligase KEG exerts negative control over abscisic acid (ABA) signaling by directly targeting the transcription factor ABI5 for ubiquitination and subsequent degradation (56). In addition, the rice E3 ligase OsRING113 ubiquitinates APIP5 to enhance broad-spectrum disease resistance (57). Our findings demonstrate that the RING-type E3 ligase TRIM37 interacts with CREB and ubiquitinates this transcription factor, promoting its degradation through the 26S proteasome pathway. Notably, a RING-domain inactive mutant of TRIM37 (C25S/C28S) completely abolished both CREB ubiquitination and degradation, providing definitive evidence that its E3 ligase activity is indispensable for this regulation. Single lysine mutations at K48 or K63 each partially reduced degradation, which is consistent with the common observation that multiple ubiquitination sites often act redundantly. These data do not weaken the conclusion that TRIM37 directly ubiquitinates CREB; rather, they suggest that additional lysine residues may also contribute. These results suggest that E3 ligases may play an underappreciated role in the regulation of transcription factors in insects. Therefore, further investigation of the role of these enzymes, and the UPS more broadly, within key regulatory cascades is warranted.

Our research provides an example of the posttranslational modification of a key transcriptional regulator of genes involved in insecticide resistance and insect reproduction. Numerous studies have implicated genes encoding detoxification enzymes in insecticide resistance (38, 39). The upregulation of these genes in resistant insect strains is commonly associated with *cis*- or *trans*-acting variants. However, how master regulatory genes that control, in *trans*, key detoxification pathways are themselves regulated remains poorly understood.

Genes involved in critical functions, such as xenobiotic resistance and other essential physiological processes, are predominantly regulated by core transcription factors (TFs), primarily from the nuclear receptor (NR), bHLH-PAS, and bZIP superfamilies (58). Previous research has shown that the bZIP TF CREB plays a key role in regulating xenobiotic detoxification in *B. tabaci* (40). Furthermore, CREB was shown to be itself regulated by phosphorylation through the action of components by the mitogen-activated protein kinase (MAPK)-directed signaling pathway (40). Thus, combining our results with this previous work, reveals how the regulation of transcription factors is orchestrated. This involves both in the xenobiotic response and other key physiological processes, which are subject to multiple layers of control mediated by diverse mechanisms including phosphorylation and ubiquitination. Specifically, the MAPK-dependent phosphorylation of CREB (40) and the TRIM37-mediated ubiquitination described here represent two cooperating posttranslational controls. Among the three E2s that interact with TRIM37, only E2-3 is functionally relevant in the ovary due to its high tissue-specific expression (*SI Appendix,* Fig. S9), defining the E1-1/E2-3/TRIM37 cascade. Moreover, although not experimentally tested in this study, we cannot exclude the possibility of feedback regulation; for example, metabolic products of CYP6CM1 might influence TRIM37 expression or activity, which could be explored in future work. This complexity could contribute to the precise and dynamical control of gene expression.

Our research also reveals how key *trans*-regulators of insecticide resistance genes have pleiotropic effects, controlling diverse biological processes by regulating the expression of multiple different genes. In this regard, recent research has shown that field-evolved resistance to neonicotinoids in *B. tabaci* MED is accompanied by a reproductive cost regulated by the expression of the key oogenesis gene *Vasa* (44). Our results provide additional evidence that modulation of *Vasa* expression can lead to a reproductive cost by causing abnormal ovarian development. Here, we show that CREB directly binds to the *Vasa* promoter and represses its transcription. The increased ChIP-qPCR signal in resistant strains (Fig. 3*H*) may be partially attributable to the higher total CREB protein levels in these strains (Fig. 3*D*), though we cannot exclude the possibility of enhanced binding affinity or other mechanisms. Collectively, these findings reveal that the transcription factor CREB plays a less exploredrole as a molecular switch in resistant *B. tabaci* MED, resulting in enhanced resistance traits and negative impacts on reproduction. These results provide insights into the molecular processes underpinning evolutionary trade-offs, in this case resistance to insecticides versus reproductive fitness. The finding that a key regulator of CREB, TRIM37, is downregulated in resistant strains is intriguing given the role of members of the E1-E2-E3 ubiquitination cascade in numerous physiological processes (53).

An outstanding question raised by our study is what causes the downregulation of TRIM37 in neonicotinoid-resistant strains? While we do not yet have a mechanistic answer, several possibilities can be considered. First, cis-regulatory mutations (e.g., in the promoter or enhancer regions of the TRIM37 gene) could affect its transcription. Second, trans-acting factors-such as altered expression or activity of upstream transcription factors-might repress TRIM37 expression in resistant strains. Third, epigenetic modifications, including DNA/RNA methylation or histone modifications, could silence TRIM37 without changing the DNA sequence. Additionally, noncoding RNAs (e.g., microRNAs or long noncoding RNAs) might posttranscriptionally regulate TRIM37 levels. Dissecting these possibilities will be an important direction for future research, and understanding the regulatory logic of TRIM37 repression may reveal novel targets for resistance management.

Of note, downregulation of TRIM37 was previously observed in the TH-R strain (MEAM1 cryptic species) selected with thiamethoxam (48), and the three resistant strains (R^#1^, R^#2,^ R^#3^, MED cryptic species) in this study consistently show reduced TRIM37 expression (Fig. 1*E*). Whether TRIM37 is downregulated in the IMR strain (40) remains unknown, as its expression was not examined in that study. Nevertheless, the consistent downregulation across different cryptic species and selecting agents suggests that TRIM37 repression may be a conserved feature associated with neonicotinoid resistance in *B. tabaci*. It is thus perhaps unsurprising that the evolution of resistance involving these “master regulators” is associated with a fitness cost. Indeed, given that abnormal E1-E2-E3 cascade activity or ubiquitination has been linked to many human diseases (51), further investigation of the fitness of insecticide resistant *B. tabaci* is warranted to identify other potential fitness costs associated with modified ubiquitination. Especially, given the mechanistic complexity of the ubiquitination system, in which E3 ligases constitute the largest and most substrate-specific component, the investigation of a single E3 ligase only provides a limited perspective. Future studies should therefore characterize a broader array of ubiquitination-related genes (including additional E3 ligases) to elucidate their distinct contributions and the specific fitness costs associated with different ubiquitin-dependent pathways. Furthermore, the potential compensatory role of deubiquitination in modulating these fitness trade-offs warrants systematic examination.

The E1-1/E2-3/TRIM37 cascade offers a new conceptual target for pest control, but translational application faces three major challenges. First, the evolution of E1/E2/E3 enzymes is highly conserved, so inhibitors pose risk off-target toxicity to pollinators and humans. Second, specificity-TRIM37 belongs to a large family of TRIM ligases; selective agonism is difficult. Third, evolutionary pressure-targeting a central pathway may drive rapid resistance escape. These challenges are serious but not insurmountable. RNAi using whitefly-specific sequences could bypass conservation issues by targeting mRNA rather than protein function. Structure-guided design might improve selectivity, though this remains a major hurdle. Rotational use with existing insecticides within IPM could slow resistance evolution. Moreover, the resistance–fitness trade-off we uncovered means that pausing the intervention would favor susceptible genotypes, adding a natural brake. Thus, while no immediate product is at hand, the cascade provides a mechanistically grounded proof of principle for ubiquitin-based resistance management-a foundation for future cross-disciplinary efforts.

In conclusion, our results reveal the role of the posttranslation modification ubiquitination in regulating a key transcription factor in insects and its functional outcomes (Fig. 6). From an applied perspective, our findings also provide potential new targets for control interventions against a global crop pest.

## Materials and Methods

A detailed description of insects, insecticides, bioassays, life-table study, evaluation of fitness costs, female fecundity assessment, ovarian development and light microscopy, gene cloning, phylogenetic and bioinformatic analysis, qRT-PCR, spatiotemporal expression profiling, western blot, antibody availability, immunofluorescence, RNAi, Co-IP assays, cell culture and transfection, plasmid construction, dual-luciferase reporter assay, promoter analysis, TF analysis, binding site analysis, recombinant protein preparation, EMSA, ChIP-qPCR, in vivo total ubiquitination level assays, in vitro ubiquitination assays, Yeast two-hybrid (Y2H) assays, statistical analyses, and data visualization are described in *SI Appendix*, *Materials and Methods*.

## Supplementary Material

Appendix 01 (PDF)

Dataset S01 (TXT)

## Data Availability

The multiple sequence alignment used for phylogenetic analysis (*SI Appendix*, Fig. S4*C*) is provided as Dataset S1 (TRIM37_alignment.fasta). All other data are included in the manuscript and/or supporting information.
