# Supplementary material for: Ubiquitination plays a key role in an insecticide resistance fitness cost
Source: Proc Natl Acad Sci U S A. 2026 Jul 30;123(31):e2536664123. doi: 10.1073/pnas.2536664123 (PMC13439105; doi:10.1073/pnas.2536664123)
Supplement: Supplementary file 1 [file pnas.2536664123.sapp.pdf]

Supporting Information for

Ubiquitination plays a key role in an insecticide resistance fitness cost

Jinyu Hu[1,†], Buli Fu[1,2,†], Qi Su[3,†], Xindi Guo[4,†], Chengjia Zhang[1,†], Qimei Tan[1], Rong Zhang[1], Hantang Lu[1], Peipan Gong[1], Shaonan Liu[1], Xuguo Zhou[5], Ralf Nauen[6], Youjun Zhang[1], Chris Bass[7]*, Xin Yang[1,*]

*Corresponding authors: C.Bass@exeter.ac.uk (C.B.), yangxin@caas.cn (X.Y.).

This file includes:

Materials and Methods
Figures S1 to S12
Tables S1 to S15
SI References

SI Materials and Methods

Insect strains. During the period from 2019 to 2021, four distinct *B. tabaci* MED populations were collected from various commercial vegetable-growing regions. This study included one field-susceptible strain (S) and three field-resistant strains ($R^{\#1}$, $R^{\#2}$, and $R^{\#3}$), classified based on their responses to seven neonicotinoids in insecticide bioassays. To preserve the resistance levels, the resistant strains were regularly challenged with imidacloprid or thiamethoxam at a concentration of 500 mg/L through root-irrigation of cotton seedlings, with a monthly interval. Both the resistant and susceptible strains were cultivated on cotton seedlings within nylon enclosures in a greenhouse set at 25°C, under a 14-hour light to 10-hour dark cycle, and with 70% humidity.

Insecticide bioassays. For insecticide bioassays, seven neonicotinoid compounds (imidacloprid, thiamethoxam, clothianidin, acetamiprid, dinotefuran, nitenpyram and thiacloprid) were employed. These technical-grade substances (of 95% purity) were procured from Shanghaiyuanye BioTechnology (Co., Ltd., China). To assess the susceptibility of *B. tabaci* to these insecticides, all bioassays were performed on adult whiteflies (of mixed sexes) using a feeding technique. Specifically, each insecticide was initially dissolved in acetone to create a stock solution of 10000 mg/L. This stock solution was then diluted with a diet solution—comprising 5% yeast extract and 30% sucrose (by weight)—to achieve the desired test concentrations ranging from 1 to 400 mg/L. Approximately 60 µL of the prepared solution was applied to the exterior surface of Parafilm stretched across the opening of a glass vial (50 mm in length and 15 mm in diameter). An additional layer of Parafilm was applied over the initial membrane to create a feeding pouch. Subsequently, approximately 20 adult whiteflies (aged 5 to 10 days) were placed into the bioassay tube. The open end of the tube was then sealed with a third layer of Parafilm. A minimum of seven different concentrations for each insecticide were tested, with four replicates per concentration, each containing approximately 20 adults in a bioassay tube. For the control group, 60 µL of the diet solution alone was applied. All bioassay tubes were subsequently placed inside black plastic containers (55 mm in length and 20 mm in diameter) and kept in the greenhouse as previously described. After 48 hours, the number of surviving and total insects was recorded, and the mortality rate for each treatment was determined. Insects were considered dead if they failed to move when gently stimulated with a fine-hair brush

(1).

Assessment of resistance levels in field-evolved populations of *B. tabaci*. To determine if field populations of *B. tabaci* have developed resistance to insecticides, bioassays were conducted on all field-collected samples to assess their susceptibility to seven neonicotinoid compounds. Using the sensitivity baseline established for the laboratory susceptible strain, the resistance level (expressed as the resistance ratio, RR) was calculated by dividing the LC_{50} value of the field samples by that of the laboratory susceptible strain. The classification of insecticide resistance levels was adapted from Torres-Vila (2) with minor adjustments: RR < three-fold was considered susceptible, RR = three- to five-fold indicated a minor resistance level, RR = five- to ten-fold signified a low resistance level, RR = 10- to 30-fold represented a moderate resistance level, RR = 30- to 160-fold was categorized as a high resistance level, and RR > 160-fold was deemed an extremely high resistance level.

Life-table analysis. The ‘age stage-two-sex’ life table study developed by Chi (3) was used to investigate the population parameters for the *B. tabaci* MED strains. In summary, around 80 newly emerged adults (5 days old) from four experimental strains were introduced into a clip cage to oviposit on a cotton leaf. After a 6-hour period, all adults were removed from the leaf. Approximately 15 eggs were left on the leaf, and any additional eggs were eliminated using a stereomicroscope at 10X magnification. Each remaining egg was then monitored for larval development and survival rates. Upon hatching, the nymphal stages were identified based on their body size relative to the previous instar. After the adults emerged, they were collected daily, and a male-female pair was placed into a fresh 5 mL tube with a cotton leaf to encourage mating and oviposition. The cotton leaves were replaced daily, and the number of surviving adults and eggs laid were documented daily until the adults' death. A minimum of 120 eggs were examined per strain in each trial. All life-table study samples were kept in a climate-controlled chamber at 25°C, with a 14-hour light to 10-hour dark cycle and 70% humidity.

Life-table parameters and evaluation of fitness cost. The TWO-SEX-MS program, as formulated by Chi (4), was utilized for the analysis of the raw biological data. The program facilitated the computation of various life-table parameters, including the age-stage-specific survival rate (s_{xj})—with x representing age and j representing stage—,

the age-specific survival rate (l_x), the age-specific fecundity (m_x), and key life-table parameters such as r (the intrinsic rate of increase), λ (the finite rate of increase), R_0 (the net reproduction rate), and T (the mean generation time), using the following equations:

$$l_x = \Sigma s_{xj}$$

$$m_x = \Sigma s_{xj} f_{xj} / s_{xj}$$

$$\sum_{x=0}^{\infty} e^{-r(x+1)} l_x m_x = 1$$

$$R_0 = \sum_{x=0}^{\infty} l_x m_x$$

$$T = (\ln R_0) / r$$

$$\lambda = e^r$$

The standard errors (SEs) for all life table parameters—such as r, λ, R_0, T, adult longevity, and fecundity—were determined through a bootstrapping method, which involved 100,000 resamples. A paired bootstrap test was employed to identify significant differences among populations, focusing on the confidence intervals of these differences. For graphical representation, SigmaPlot v. 12.0 software (Systat Software, San Jose, CA, USA) was utilized. The relative fitness (*Rf*) values were derived using the subsequent equations:

Rf_r = r value of the resistant strain / r value of the susceptible strain

R_fR_0 = R_0 value of the resistant strain / R_0 value of the susceptible strain

where Rf_r and R_fR_0 were calculated by the intrinsic rate of increase (r) and the net reproduction rate (R_0), respectively.

Female fecundity assessment. For the experiments, both female and male whiteflies were housed together in a vial, providing an opportunity for mating over a 4 hour period. Following this, a female was randomly selected and transferred to a 5 mL centrifuge tube for ongoing cultivation with a section of fresh cotton leaf, which was replaced daily. The daily egg count was conducted using a microscope at 20X magnification. A minimum of 60 females were assessed per treatment to ensure the accuracy of the data.

Ovarian development and light microscopy. Adult female whiteflies were transferred to centrifuge tubes for further procedures. After being anesthetized on ice for 30 minutes, each female's ovaries were dissected in 0.1M phosphate-buffered saline (PBS, pH 7.2). Ovarian development was evaluated according to the method

described by Guo *et al*. (5), using the Leica historesin standard kit (Leica Biosystems, Germany) to count the number of ovarioles and oocytes in each female's ovary. The morphology of the ovaries, ovarioles, and oocytes at various stages or under different treatments was captured using a Leica microscope. Images were taken at 100X magnification. A minimum of 60 female adults were examined for each treatment group.

Gene cloning. RNA was extracted from 60 whole adult *B. tabaci* MED (with a female to male ratio of approximately 1:1, aged 3-10 days post-eclosion) using the standard TRIzol method (Invitrogen, Carlsbad, CA, USA), with its concentration measured using a NanoDrop 2000c spectrophotometer (Thermo Fisher Scientific Inc., Waltham, MA, USA). The RNA was then reverse-transcribed into cDNA with oligo (dT) primers and SuperScript II Reverse Transcriptase, which included a gDNA Eraser step (Takara Biotech, Tokyo, Japan). Initially, the putative gene sequences were computationally identified from *B. tabaci* MED transcriptome data (6) and subsequently cloned by RT-PCR using primers detailed in Table S8.

Quantitative real-time PCR (qRT-PCR). For qRT-PCR analysis, 150 adult whiteflies (in three biological replicates of 50 each) from various strains were employed. The 20 μL qRT-PCR reaction mix included 1 μL diluted cDNA, 10 μL SYBR Green Real-time PCR Master Mix (Tiangen), and 0.3 μL of each primer. The specific qPCR primer sequences are provided in the supplementary Table S8. The reactions were conducted on an ABI Q3 real-time system (Applied Biosystems, Thermo Fisher Scientific, Waltham, MA, USA) with the following thermal profile: a 15-minute denaturation at 95°C, followed by 40 cycles of 30 seconds at 95°C and 40 seconds at 60°C. A cDNA dilution series was utilized to create a relative standard curve, and PCR efficiency was calculated to convert Ct values into relative quantities. The amplification efficiency of the qPCR primers was assessed using a six-point 3-fold serial dilution (1:1, 1:3, 1:9, 1:27, 1:81, and 1:243) with four cDNA replicates. The amplification efficiency was calculated using the formula E = (10[-1/slope]-1). Primers with 95–105% efficiency and correlation coefficients above 0.95 were selected for use. The expression levels of the genes of interest were normalized to two housekeeping genes (*EF1α* and *RPL29*) and calculated using the $2^{-\triangle\triangle Ct}$ method based on three biological replicates. The qRT-PCR primers were designed to amplify fragments between 90 to 180 base pairs, with their amplification efficiencies detailed in the supporting information (Table S8).

Protein domain prediction, phylogenetic analysis and spatiotemporal expression profiling. Following gene cloning, the conserved domains of the target proteins were predicted using the National Center for Biotechnology Information (NCBI) Conserved Domain Database (https://www.ncbi.nlm.nih.gov/Structure/ cdd/wrpsb.cgi). The identification of transmembrane domains (TMDs) in the candidate genes was carried out with TMHMM (http://www.cbs.dtu.dk/services/TMHMM/). The theoretical isoelectric points (pIs) and molecular weights (Mw) were determined using ExPASy (https://web.expasy.org/protparam/). Homologous amino acid sequences of the target genes were retrieved from NCBI for phylogenetic analysis. For this analysis, the neighbor-joining method with 1,000 bootstraps was applied to construct a phylogenetic tree in MEGA7. A total of 2,000 eggs (E), 300 1st to 2nd instar nymphs (N1-2), 200 3rd-instar nymphs (N3), and 200 4th-instar nymphs (N4), along with 100 female adults (F) and 150 male adults (M) from strain S, were collected for RNA extraction and cDNA synthesis. Additionally, around 2,000 individuals of each whitefly tissue type-head, thorax, and abdomen-were dissected. These samples were employed to measure relative gene expression levels via qPCR, with a minimum of three biological replicates for each condition.

Western blot. For each sample, 200 adult whiteflies were processed to extract proteins using the ProteinExt Mammalian Total Protein Extraction Kit (Beyotime, China) according to the manufacturer's protocol. The protein extracts, after supplementation with SDS-PAGE loading buffer (CWBIO), were denatured by boiling for 8-10 minutes at 100°C following protein concentration determination with the BCA Protein Assay Kit (Beyotime, China). The proteins were then resolved on a 4-15% SDS-PAGE precast gel (Lablead Biotech., China) and transferred onto PVDF membranes (Merck Millipore, Germany). The membranes, containing the target proteins, were incubated with primary rabbit-polyclonal antibodies specific to the target genes in antibody diluent at 4°C overnight, followed by incubation with goat anti-rabbit IgG-HRP (CWBIO, 1:5000) secondary antibody. The total protein and phosphorylation levels of the target proteins were detected using the SuperSignal West Pico Chemiluminescent Substrate (Thermo Fisher, USA), and the images were captured using the Tanon-5200 Chemiluminescent Imaging System (Tanon). Rabbit polyclonal antibodies for TRIM37(1:5000), CREB (1:2500), CYP6CM1 (1:5000), Vasa (1:5000) proteins, and phosphorylated forms P-CREB (1:2000) were raised against synthetic peptides based on specific amino acid sequences or were commercially available. A β-actin (Abcam, 1:5000) antibody served

as the internal control primary antibody. Additional information on these antibodies is detailed in the supporting information (Table S12).

Immunofluorescence (IF) assays. Under a Leica microscope, whitefly ovaries and midguts were carefully dissected from female adults. After being permeabilized with 75% ethanol for 25 minutes, the tissues were fixed in 4% paraformaldehyde (PFA) for 2.5 hours at ambient temperature. Following three rinses with PBST, the samples were blocked with the Immunol Staining Blocking Buffer Kit (Beyotime, China) at 4°C for 3 hours and then incubated with primary antibodies specific to Vasa (1:600) at 4°C overnight. Subsequently, the samples were incubated with the secondary antibody Alexa Fluor® 555 goat anti-rabbit IgG (1:500) (Abcam) for 2 hours at room temperature within a light-protected box and washed five times with PBST. The ovary samples were mounted on adhesive microslides and counterstained with DAPI (Abcam) at room temperature. Fluorescent images were obtained using a LSM 980 confocal microscope (Leica, Germany) at 10X magnification, with high-resolution images captured at 20X magnification.

RNAi experiments. Double-stranded RNAs (dsRNAs) were produced utilizing the T7 RiboMAX™ Express RNAi System (Promega, WI, USA) in accordance with the manufacturer's guidelines. The primers employed for dsRNA synthesis are detailed in Table S8. The dsRNAs were administered to adult whiteflies that had recently emerged (within 1 day of eclosion). Post dsRNA treatment, female whiteflies were selected for RNA extraction and cDNA synthesis. The subsequent relative quantification of gene expression was conducted using qPCR as previously described. Once the efficacy of the RNAi was confirmed, these dsRNA-fed females were subjected to further analysis of their reproductive output and ovarian development employing the aforementioned methodologies.

Molecular docking and simulation analysis of protein-protein interactions. The protein sequences of E1-1, E2-3, E2-14, and E2-19 were obtained from the Whitefly Genome Database (http://www.whiteflygenomics.org/cgi-bin/bta/index.cgi); their corresponding gene IDs are listed in Table S8. The sequences of TRIM37 and CREB were cloned from *B. tabaci* as described above. Since no experimentally resolved structures suitable for molecular docking were available for these proteins, their three-dimensional structures were predicted using ProteinX, an open-source method that

reproduces the performance and approach of AlphaFold3, achieving comparable or even better results in multiple benchmarks while ensuring academic open-access compliance. The predicted structures were subsequently energy-minimized using Rosetta Relax. For docking preparation, the 3D structures were processed in PyMOL to remove water molecules, add hydrogen atoms, and export the models in PDB format. HADDOCK was first used for rigid-body docking to adjust the initial relative orientation of each protein pair. Based on the rigid-docking results, flexible docking was then performed using RosettaDock. The final docking poses were selected using Rosetta's built-in scoring module.

Protein–protein interactions (hydrogen bonds and salt bridges) were analyzed with Ligplot. The overall binding free energy of each complex was calculated using the Interface_analyzer module of FoldX. Final structural representations of the docked complexes were prepared with PyMOL.

Co-immunoprecipitations (Co-IP) assays. Immunoprecipitation (IP) experiments were performed to investigate the direct protein-protein interactions. Proteins from the susceptible strain S of whiteflies were isolated using the ProteinExt Mammalian Total Protein Extraction Kit by Beyotime, China, adhering to the protocol provided by the manufacturer. The IP process was employed to extract TRIM37 and Vasa proteins from the total lysate protein samples, as per the guidelines of the Immunoprecipitation Kit from BEAVER Biomedical Engineering, China. In summary, equivalent quantities of total protein from adult whiteflies were mixed with specific antibodies targeting the proteins of interest and incubated overnight at 4 °C. This was followed by a 3 hour incubation with protein A/G-magnetic beads, also from BEAVER Biomedical Engineering, China. Afterward, the samples were washed five times with 1x PBS and centrifuged at 2,500 rpm for 10 minutes at 4 °C to remove the supernatant. The agarose suspension containing the immune complexes was then rinsed with NP-40 lysis buffer to eliminate any unbound proteins. The precipitated proteins were eluted using glycine, with the samples being incubated for 10 minutes three times, ensuring frequent mixing, followed by gentle centrifugation. Neutralization of the eluate was achieved by adding an equal volume of Tris buffer at pH 8.0. The beads were further neutralized by two washes with the lysis buffer before being combined with the eluate. Western blot analysis was then conducted to identify the precipitated target proteins, with the IgG antibody serving as a negative control in the process.

S2 Cell culture and transfection. S2 cell lines, originating from the embryonic tissues of *Drosophila melanogaster*, were cultured in Hyclone SFX-insect medium (Thermo Fisher) at a temperature of 27°C. These cells were routinely passaged into new medium every 48 hours. Before transfection, S2 cells were plated at a concentration of approximately 6 x 10^5 cells per well in 300 μL of Hyclone SFX-insect medium within a 24-well plate. Transfections were carried out using Lipofectamine 2000 transfection reagent (Invitrogen) following the manufacturer's protocol. All plasmids were adjusted to a uniform concentration of 100 ng/μL and then introduced into the S2 cells with the transfection reagents at a ratio of 1:3 (2 μL Lipofectamine 2000 to 6 μL plasmid) in 100 μL of cell medium. Following this, the transfection lipid was added to a 24-well plate containing 300 μL of the aforementioned Hyclone SFX-insect medium. The transfection mixtures were incubated at 27°C for 48 hours before being utilized for dual-luciferase reporter assays.

Promoter analysis of *Vasa*. A total of 300 adult whiteflies were employed to obtain genomic DNA utilizing the Genomic DNA Extraction Kit from TIANGEN, following the manufacturer's guidelines. To pinpoint the core promoter region, the upstream promoter region of the *Vasa* gene was segmented into nine distinct segments, ranging in length from about 200 to 2000 base pairs. These promoter regions were then amplified via PCR using PrimeSTAR Max DNA Polymerase from Takara, starting from genomic DNA of *B. tabaci*. The PCR products of these promoter regions were subsequently subcloned and integrated into the firefly luciferase-based reporting vector pGL4.10-Basic from Promega, employing homologous recombination facilitated by the Infusion Kit - also from Takara. The plasmids containing the promoter regions were purified using the EndoFree Mini Plasmid Kit II by TIANGEN, and their concentration was standardized to 100 ng/μL for subsequent dual-luciferase reporter assays. The pGL4.73 vector from Promega, which harbors a Renilla luciferase gene, served as an internal reference. The design of all primers necessary for promoter analysis and vector construction was accomplished using the Primer 5.0 software by Premier Biosoft, with details provided in the supplementary material (Table S8).

Transcription factor screening and dual-luciferase reporter assays. A selection of 9 transcription factors (TFs) from the nuclear receptor (NR), basic leucine zipper (bZIP), and bHLH-PAS families was analyzed (Table S7). The coding sequences (CDS) of

these TFs were obtained through PCR amplification from the genomic DNA of *B. tabaci*, utilizing PrimeSTAR Max DNA Polymerase from Takara, Japan, in accordance with the manufacturer's guidelines. The resulting PCR products were cloned and inserted into the pAC5.1b/V5/His B expression vector provided by Invitrogen. The plasmids were then purified using the EndoFree Mini Plasmid Kit II by TIANGEN and diluted to a concentration of 100 ng/μL for application in dual-luciferase reporter assays. The pGL4.73 vector from Promega, which includes a Renilla luciferase gene, served as an internal reference. Primers for the TFs and vector construction were designed with the aid of the Primer 5.0 software by Premier Biosoft (Table S8). The dual-luciferase reporter assays were executed using the *D. melanogaster* S2 cell line. In essence, the promoter constructs (6 μL) and the pGL4.73 plasmid (1 μL) were co-transfected into S2 cells with the aforementioned transfection reagent to assess promoter activity, with the empty pGL4.10 vector serving as a control. For the analysis of transcription factors, the TF plasmids (6 μL), promoter constructs (6 μL), and the pGL4.73 plasmid (1 μL) were co-transfected into S2 cells to evaluate the influence of TFs on the promoter activity, with the empty pAC5.1b vector as a control. After a 48-hour transfection period, luciferase activity was quantified using a GloMax 96 Microplate Luminometer with the Dual-Luciferase Reporter Assay System from Promega, following the manufacturer's directions. The relative luciferase activity, calculated as the ratio of Firefly luciferase activity to Renilla luciferase activity for each construct, was standardized against the control group's values. Each reporter assay was conducted with a minimum of three separate replicates.

Analysis of Binding Sites. The JASPAR database, accessible online at https://jaspar.genereg.net/, was utilized to predict potential binding sequences for CREB within the core promoter region of the *Vasa* gene (as detailed in Table S10). Following this, mutated promoter fragments (listed in Table S11) were synthesized by General Biol., China, and then inserted into the firefly luciferase reporting vector pGL4.10 from Promega to confirm the binding site. In parallel, the mutant CDS sequence of the transcription factor CREB (Table S10), aimed at functionally probing the three conserved domains of this protein, was also synthesized by General Biol., China, and subsequently cloned into the pAC5.1b/V5/His B expression vector by Invitrogen. This was done to assess the regulatory impact of CREB on the core promoter of *Vasa*. Post-transfection into S2 cells, these mutant plasmid constructs were subjected to dual-luciferase reporter assays, following the

procedures previously outlined.

Recombinant protein preparation. Recombinant CREB was inserted into the HindIII sites of the pET-28a expression vector. Recombinant TRIM37 was cloned into the EcoR I restrictions sites of the pGEX-4T-1 vector. There proteins were expressed in *Escherichia coli* BL21 cells, sourced from Tsingke, China. His-CREB was subsequently purified using the His-tag Protein Purification Kit from Beyotime, China, following the manufacturer's guidelines. GST-TRIM37 was subsequently purified using BeaverBeads® GSH Kit from Beaverbio, China. The purified recombinant proteins were then utilized in Electrophoretic Mobility Shift Assay (EMSA) or *in vitro* ubiquitination assays.

Electrophoretic mobility shift assays (EMSA). Biotinylated oligonucleotide probes for both wild-type and mutant binding sites, with CY5 labeling at the 3'-terminus, were synthesized by General Biol., China (Table S11). The EMSA assay was performed using the Electrophoretic Mobility Shift Assay Kit from Invitrogen, adhering to the manufacturer's protocols. In brief, the oligonucleotide probes were combined with the purified CREB protein in EMSA/GEL-Shift binding buffer and incubated for 30 minutes at 25 °C. After incubation, the DNA-protein complexes were subjected to gel electrophoresis and detected using the LightShift Chemiluminescent EMSA Kit from Thermo Fisher, following the manufacturer's instructions. The nylon membrane containing the DNA-protein complexes was visualized with the Tanon-5200 Chemiluminescent Imaging System from Tanon, China. Additionally, the SDS-PAGE gel with the DNA-protein mixture was directly placed into the Tanon 5200 Multi Chemiluminescent Imaging System from Tanon, China, for imaging. The CY5 fluorescence was captured using a Fluorescence Imaging System with laser excitation.

ChIP-qPCR assays. To confirm the binding site of the transcription factor CREB in the promoters of *Vasa in vivo*, chromatin immunoprecipitation (ChIP) assays were performed using the EZ-Magna ChIP A/G Kit (Merck Millipore, Germany). Immunoprecipitation (IP) was performed using protein A/G-magnetic beads (Beyotime, China) conjugated with anti-CREB antibody (5 μg). Chromatin immunoprecipitated with anti-IgG antibody (5 μg) was used as a negative control. Following purification of the precipitated DNA, its relative expression was quantified by qPCR using the specific primers (Table S8) encompassing the binding sequence in core promoter region of

Vasa to confirm the direct binding of CREB to these promoter regions.

***In vivo* total ubiquitination level assays.** To test the ubiquitination of total proteins in four experimental strains of *B. tabaci*, ubiquitination assays were performed. For each sample, 200 adult whiteflies were processed to extract proteins using the ProteinExt Mammalian Total Protein Extraction Kit (Beyotime, China) according to the manufacturer's protocol. Specific methods are performed as described for western blot. The total ubiquitination level was detected with anti-Ubiquitin antibody (Table S12).

***In vitro* ubiquitination assays.** *In vitro* ubiquitination assays were followed by E3 Ligase AutoUbiquitylation Assay Kit (Abcam, ab139469), which could be used to determine the ubiquitinylation of substrate proteins specific to a particular ubiquitin E3 ligase. Reactions comprised, 5 μl 10X Ub E3 ligase buffer, 2.5 μl 20X Ubiquitin Activating Enzyme Solution (E1), 2.5 μl 20X UbcH5a (Human, Recombinant), 5 μl 10X Ubiquitin, 1 μl 50mM DTT, 2.5 μl 20X Mg-ATP, 5 μl of 6 μM Ub His-CREB protein solution, 5 μl of 6 μM Ub GST-TRIM37 protein solution, 21.5 μl dH_2O (recommended total reaction volume = 50 μL) in 0.5 mL Eppendorf tube(s) and tube contents mixed gently and incubated at 37° C for 1 hour. Assays were quenched by the addition of 50 μL 2x SDS-PAGE gel loading buffer followed by heating to 95 ºC for 5 minutes. Ubiquitinated proteins were detected by western blot using the anti-His and anti-Ubiquitin (Table S12).

Yeast two-hybrid (Y2H) assays. The yeast two-hybrid (Y2H) assays were conducted to investigate protein-protein interactions outside of a living cell environment. In summary, the target coding sequences for *TRIM37* and *CREB* were inserted into the pGADT (AD) vector to create AD-TRIM37 and into the pGBKT7 (BD) vector to generate BD-CREB. These various constructs, serving as bait and prey, were then co-transformed into the yeast strain Y2HGold. Following this, the yeast clones that had been co-transformed were diluted in a serial manner (at ratios of 1:10, 1:100, and 1:1,000) and applied onto selective media lacking Ade, His, Leu, and Trp to assess their growth. Photographs of the growth were taken between days 3 and 5 after incubation at 30 °C. The constructs pGBKT7-53 and pGADT7-T served as positive controls, while pGBKT7-lam and pGADT7-T acted as negative controls.

Statistical Analysis. The statistical significance of differences between samples were

analyzed using Student's *t*-test or one-way analysis of variance (ANOVA) with Tukey's HSD test with GraphPad Prism 8.4. All quantitative data are reported as means ± SEM from at least three independent replicates. All graphs were constructed through GraphPad Prism 8.4, SigmaPlot 12.5, Adobe Illustrator 2022 and Microsoft Office 2019.

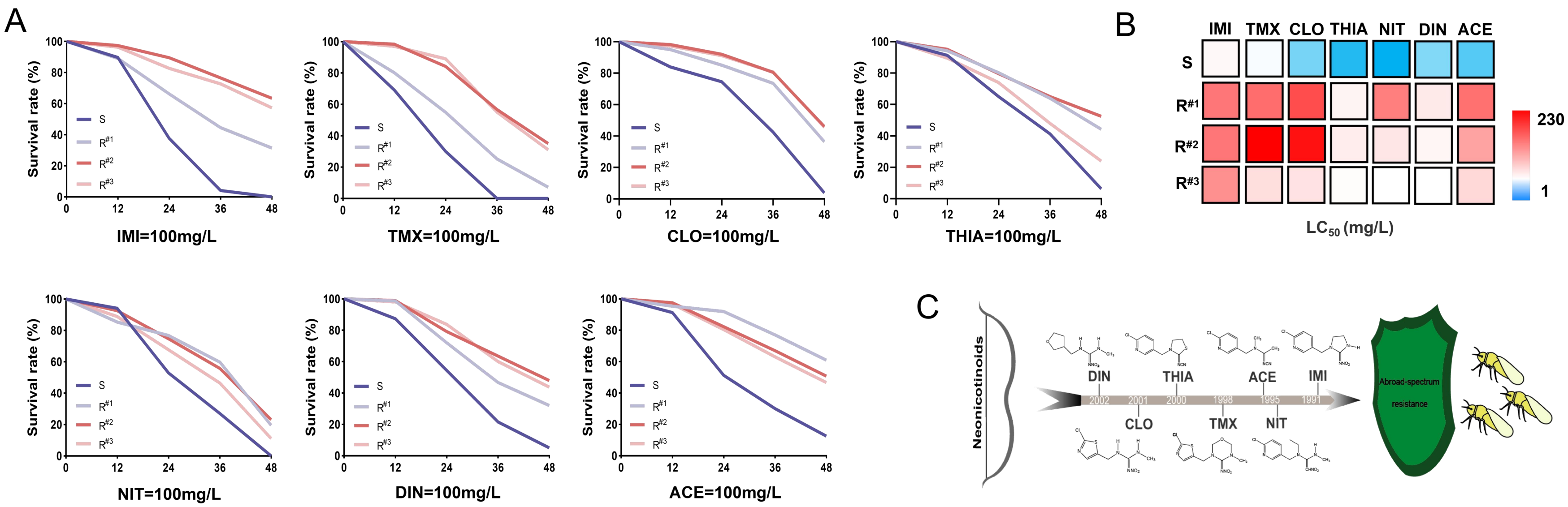

Fig. S1. Field populations of *B. tabaci* exhibit resistance to neonicotinoids. (A) The sensitivity of experimental strains (S, $R^{\#1}$, $R^{\#2}$ and $R^{\#3}$) of *B. tabaci* MED to seven neonicotinoid insecticides. Insecticides tested include imidacloprid (IMI), thiamethoxam (TMX), clothianidin (CLO), thiacloprid (THIA), nitenpyram (NIT), dinotefuran (DIN) and acetamiprid (ACE). **(B)** The comparison of values of median lethal concentration (LC_{50}) values of neonicotinoid sensitive (S) and resistant whitefly strains ($R^{\#1}$, $R^{\#2}$ and $R^{\#3}$) to seven commonly used neonicotinoids. LC_{50} values shown are relative to the reference S strain. **(C)** A schematic of the broad-spectrum resistance to neonicotinoids in *B. tabaci* MED.

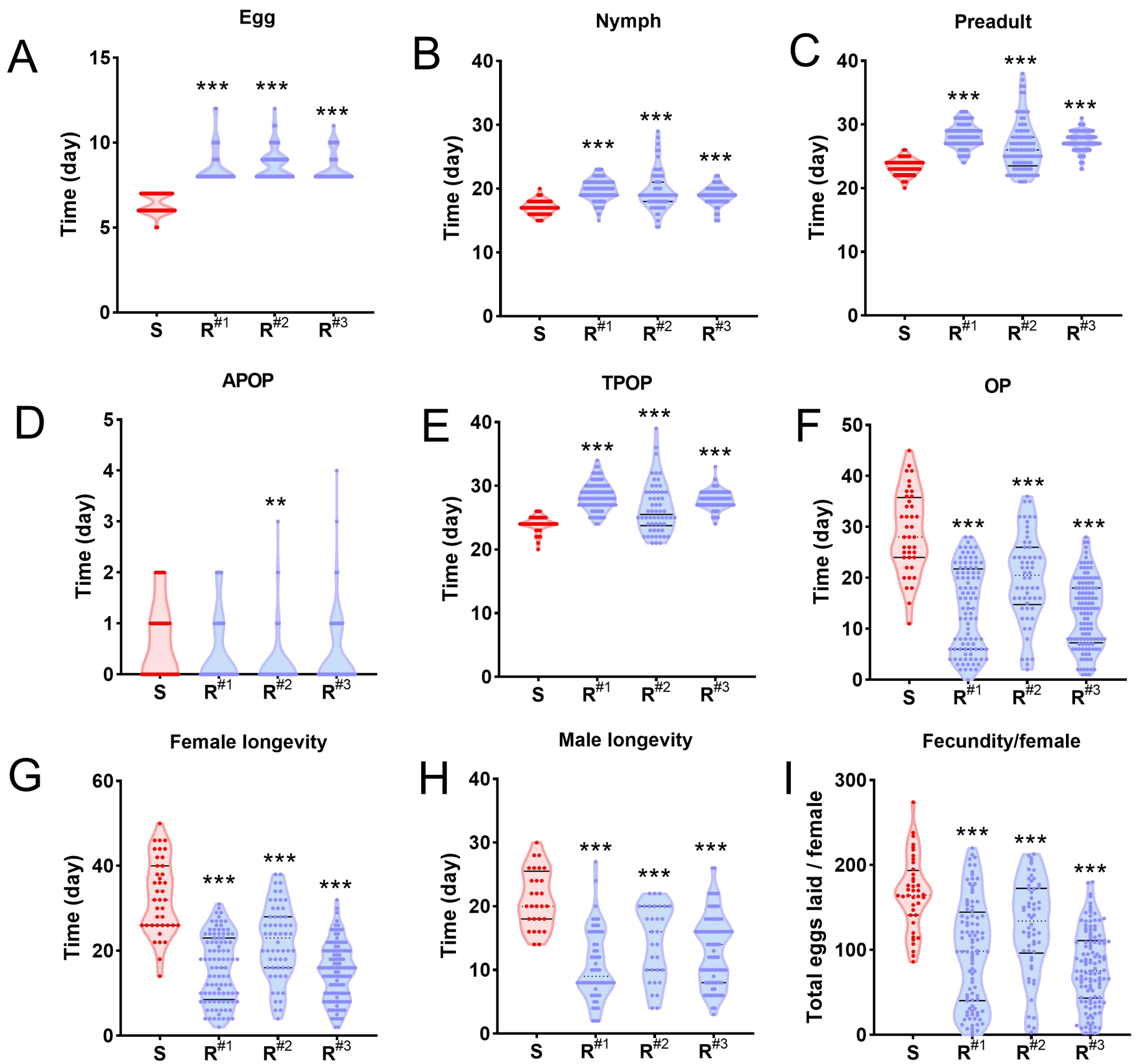

A
Egg
B
Nymph
C
Preadult
D
APOP
E
TPOP
F
OP
G
Female longevity
H
Male longevity
I
Fecundity/female
Time (day)
Total eggs laid / female
S
R#1
R#2
R#3
***
**

Fig. S2. Life-table study of the life-history traits of the field collected strains of *B. tabaci*. (A-C) Developmental duration of egg, nymph and total developmental duration of preadult (from egg to adult) stage. **(D-F)** APOP, adult pre-oviposition period; TPOP, total oviposition period; OP, oviposition period. **(G-H)** Adult longevity of female and male. **(I)** Fecundity (total eggs laid per female in its whole life). All the biological raw data were analyzed by using the TWO-SEX-MS program. Asterisks indicate significant differences as determined by Student's *t* test (**P* < 0.05, ***P* < 0.01, ****P* < 0.001), and absence of asterisks indicates no statistical significance (*P* > 0.05).

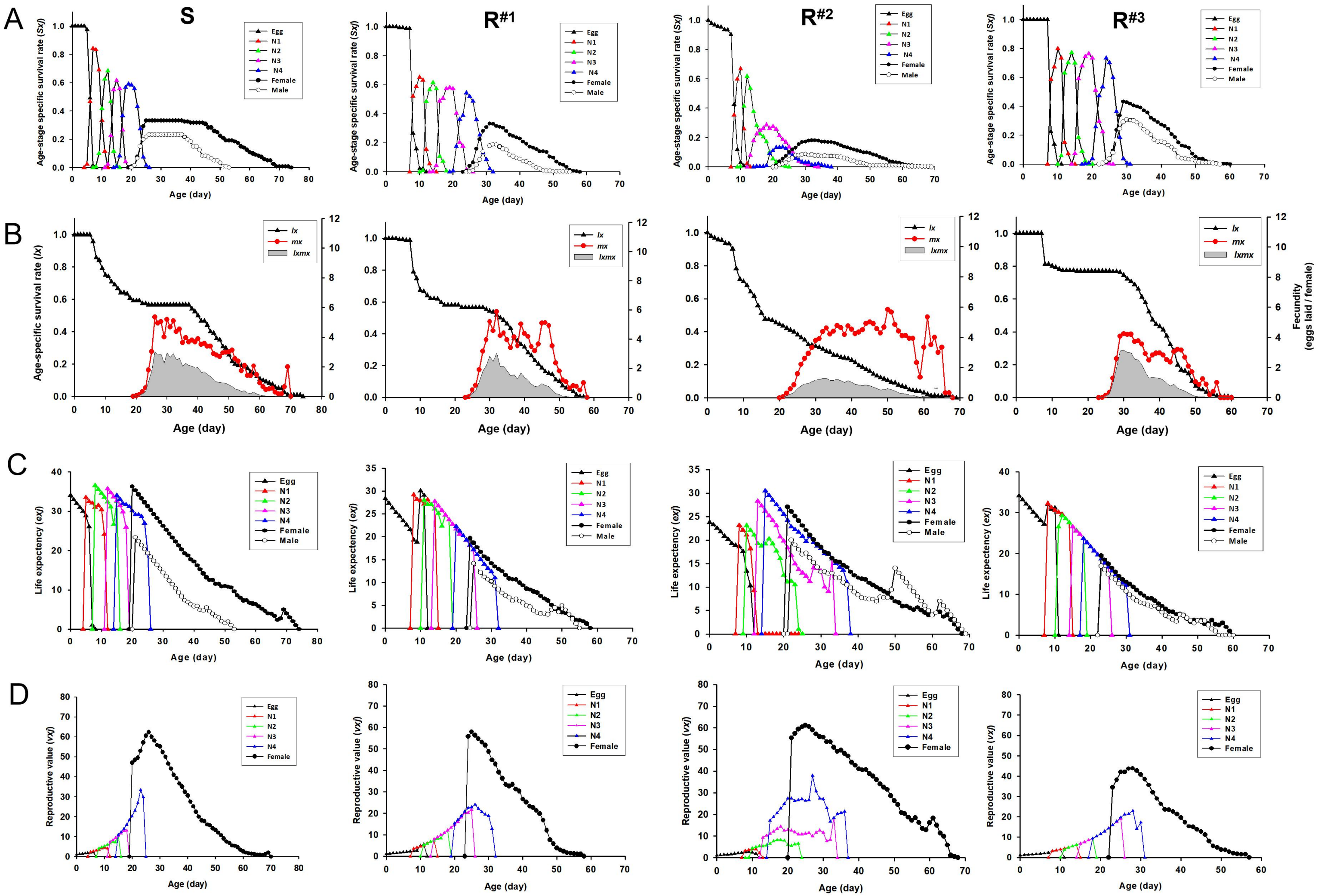

A
S
R#1
R#2
R#3
Age-stage specific survival rate (Sxj)
Egg
N1
N2
N3
N4
Female
Male
Age (day)
B
Age-specific survival rate (lx)
lx
mx
lxmx
Fecundity
(eggs laid / female)
C
Life expectancy (exj)
D
Reproductive value (vxj)

Fig. S3. Life-table analysis of survival rate, fecundity, life expectancy and reproduction value of experimental strains of *B. tabaci* MED. **(A)** Age-stage survival rate (s_{xj}). **(B)** Age-specific survival rate (l_x), fecundity (m_x) and net maternity ($l_x m_x$). **(C)** Life expectancy (E_{xj}). **(D)** Reproduction value (V_{xj}). All biological raw data were analyzed using the TWO-SEX-MS program. E: egg, N1: 1st-instar nymph, N2: 2nd-instar nymph, N3: 3rd-instar nymph, N4: 4th-instar nymph, F: female adult, M: male adult. The four experimental strains of *B. tabaci* MED comprise the experimental strains S, $R^{\#1}$, $R^{\#2}$ and $R^{\#3}$**.**

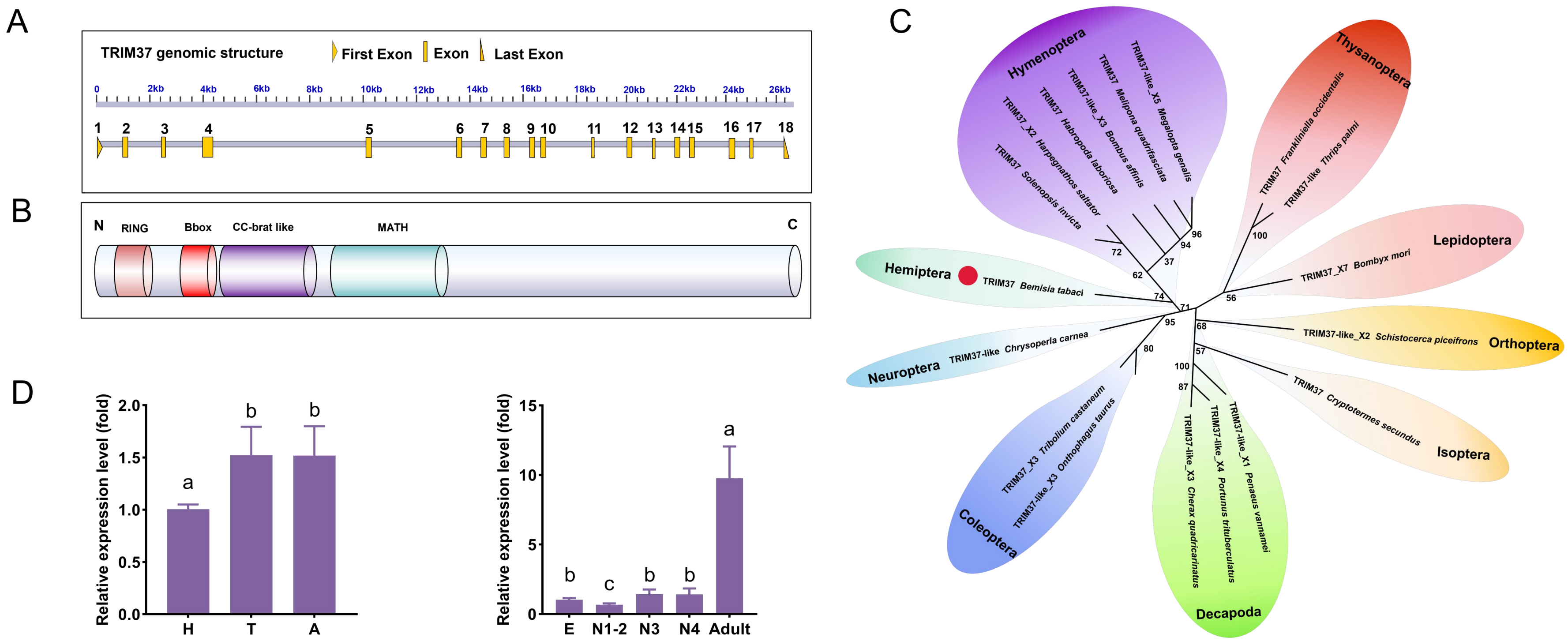

Fig. S4. Bioinformatic analysis of the E3 ubiquitin ligase *TRIM37* in *B. tabac*i MED. (A) Schematic of gene structure and the domain composition of *TRIM37*. The full-length *TRIM37* gene in *B. tabaci* MED comprises 18 exons and 17 introns, containing a 2625-bp ORF which encodes 874 amino acid residues (98.89 kDa). **(B)** Protein domain predictions show that the TRIM37 protein contains a RING domain, a Bbox domain, a CC-brat like domain and a MATH domain. **(C)** Phylogenetic analysis of *TRIM37* from various insect species. The phylogenetic tree

was constructed using the neighbor-joining algorithm (MEGA7) from full sequence alignments computed in ClustalX. Bootstrap analysis (1,000 replicates) was applied to evaluate the internal support for the tree topology. The red circle indicates *TRIM37* of the whitefly *B. tabaci* MED. **(D)** Spatiotemporal expression profiling of *TRIM37* in the susceptible strain S. H, head; T, thorax; A, abdomen; E, egg; N1-2, 1st and 2nd-instar nymph; N3, 3rd-instar nymph; N4, 4th-instar nymph; A, adult (mixed sex). All qPCR data are presented as the mean ± SEM of at least three independent experiments. Different letters in the same chart indicate significant difference at the $P < 0.05$ level determined by ANOVA and Tukey's multiple comparisons test.

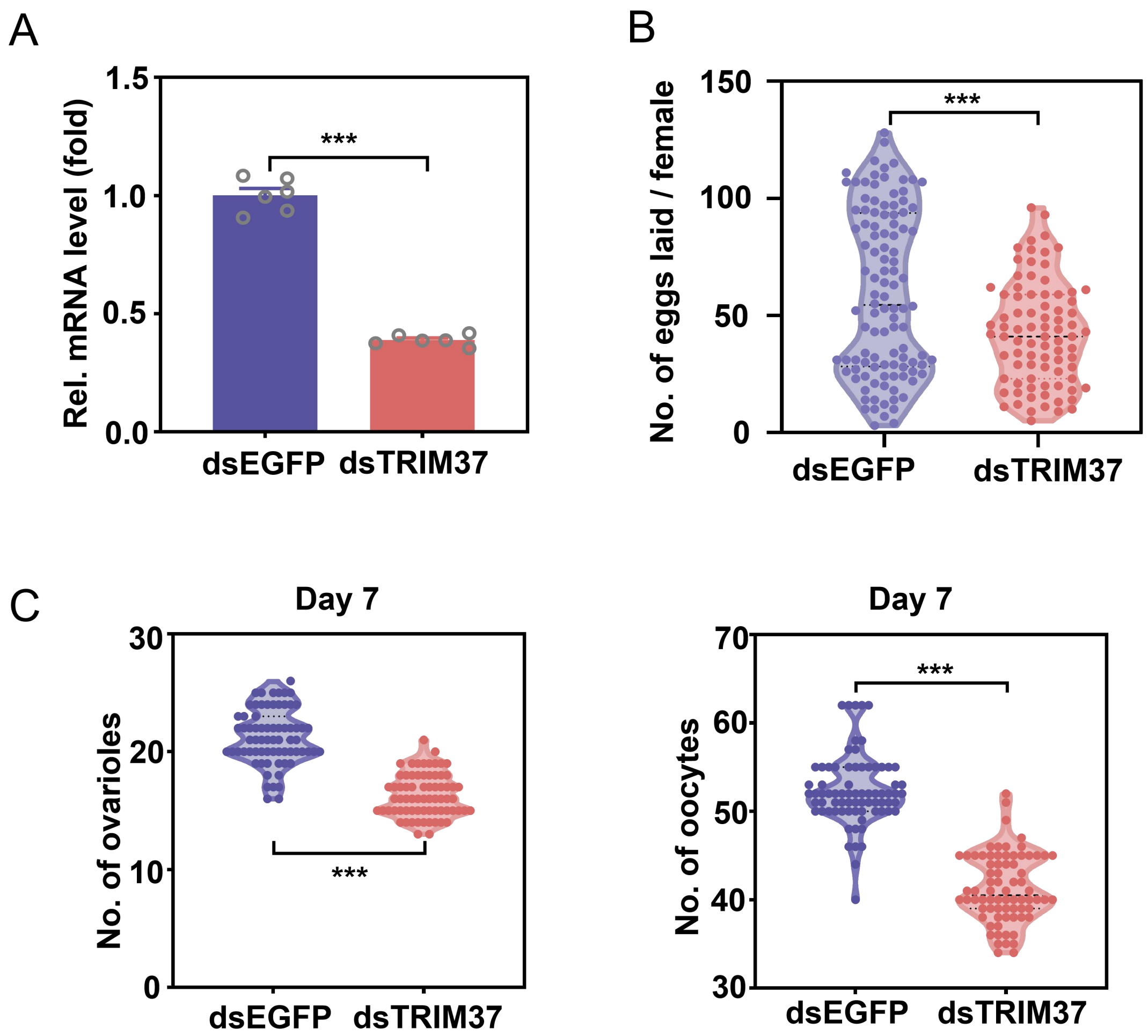

A
Rel. mRNA level (fold)
1.5
1.0
0.5
0.0
***
dsEGFP
dsTRIM37
B
No. of eggs laid / female
150
100
50
0
***
dsEGFP
dsTRIM37
C
Day 7
No. of ovarioles
30
20
10
0
***
dsEGFP
dsTRIM37
Day 7
No. of oocytes
70
60
50
40
30
***
dsEGFP
dsTRIM37

Fig. S5. The impact of RNAi knockdown of *TRIM37* on the reproductive fitness of *B. tabaci* MED. (A) Relative expression of *TRIM37* after 24 h of feeding on dsTRIM37. **(B)** The effect after RNAi knockdown of *TRIM37* on female fecundity. **(C)** Average number of ovarioles and oocytes per female sampled on day 7 after RNAi knockdown of *TRIM37*. All bioassay data (mean ± SEM) represent at least three biological replicates with approximately 60 adults in each replicate. The susceptible strain S was used in the RNAi experiments. Whiteflies fed on dsEGFP served as a negative control in RNAi experiments. All data (mean ± SEM) on ovariole number and oocyte number represent four biological replicates with at least 15 female individuals in each replication. Asterisks indicate significant differences as determined by Student's *t* test (*$P < 0.05$, **$P < 0.01$, ***$P < 0.001$), and absence of asterisks indicates no statistical significance ($P > 0.05$).

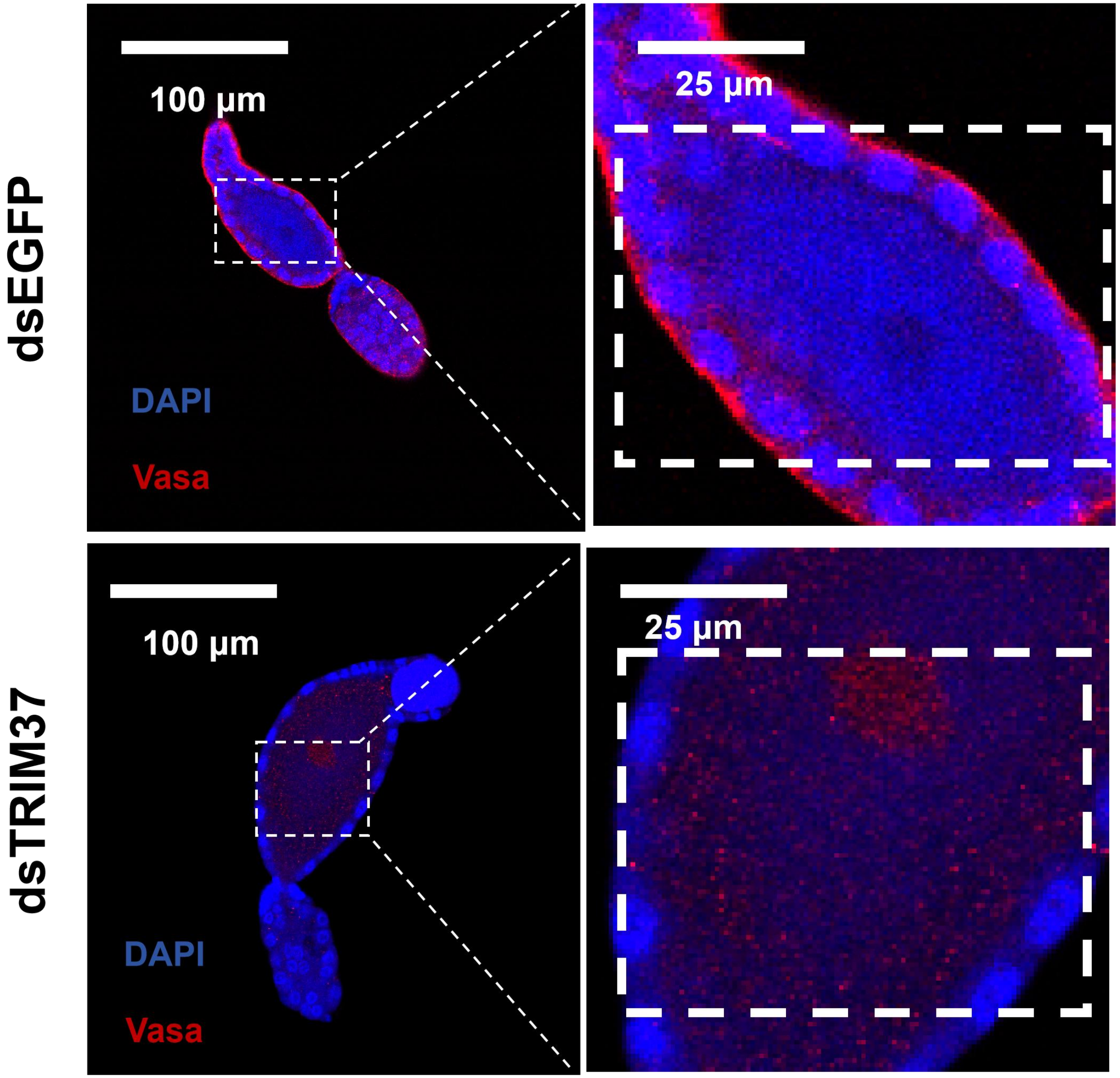
dsEGFP
100 µm
DAPI
Vasa
25 µm
dsTRIM37
100 µm
DAPI
Vasa
25 µm

Fig. S6. Immunofluorescence staining assays of Vasa protein in oocytes of female *B. tabaci* ovaries after RNAi knockdown of *TRIM37* in the S strain. Cell nuclei were stained with DAPI (blue). Target protein Vasa was stained with its primary rabbit antibody followed by the secondary antibody Alexa Fluor 555 goat anti-rabbit IgG (red). Whiteflies fed on dsEGFP served as a negative control in the RNAi experiments. Scale bars, 100 μm for original images and 25 μm for zoomed-in images.

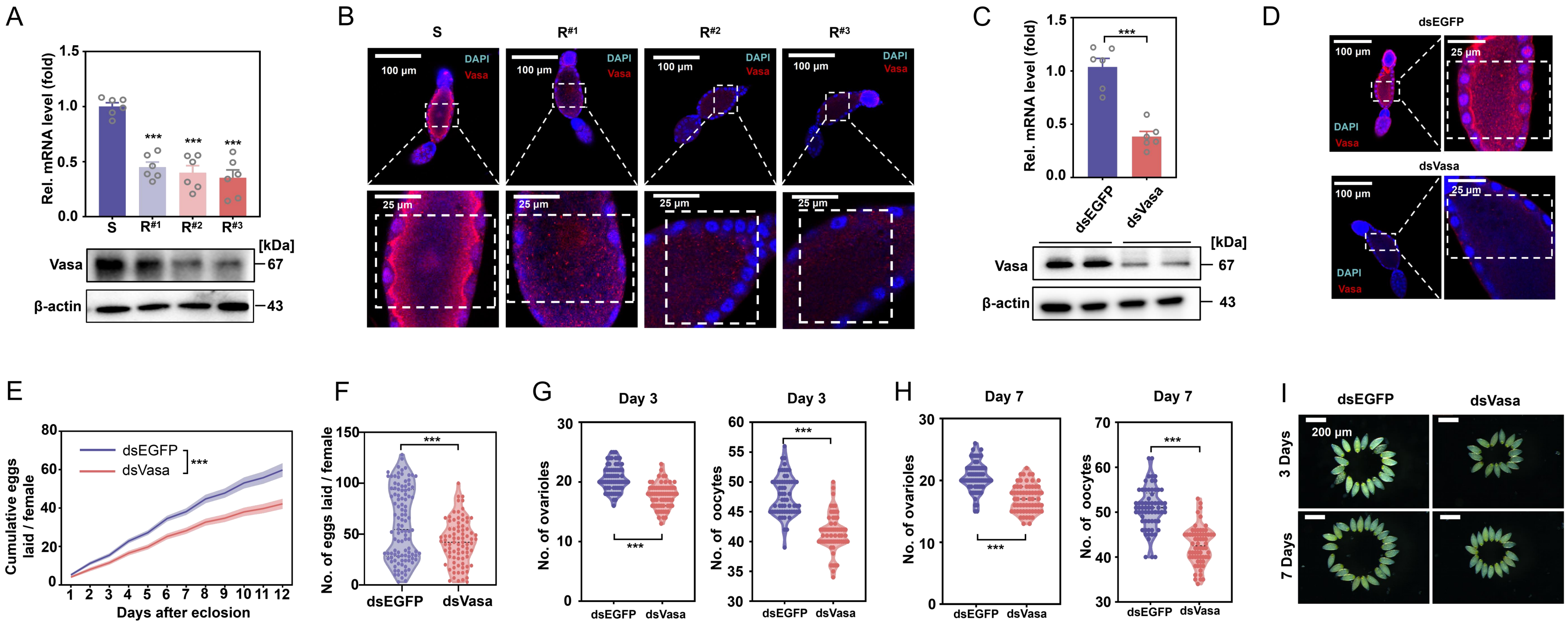

Fig. S7. Downregulation of the oogenesis genes *Vasa* confers abnormal ovarian development, leading to a reproductive cost associated with neonicotinoid resistance in *B. tabaci* MED. (A) Quantification of the expression of *Vasa* and western blot analysis of protein` level of Vasa among the experimental strains (S, $R^{\#1}$, $R^{\#2}$ and $R^{\#3}$). **(B)** Immunofluorescence staining of Vasa protein in the oocyte among the experimental strains (S, $R^{\#1}$, $R^{\#2}$ and $R^{\#3}$). Cell nuclei were stained with DAPI (blue). Target protein Vasa was stained with its primary rabbit antibody followed by the secondary antibody Alexa Fluor goat anti-rabbit IgG (red). White boxes represent the zoomed-in location of the enriched target proteins. Scale bars, 100 μm for original images and 25 μm for zoom-in images. **(C)** Relative expression of *Vasa* and western blot analysis of Vasa after

24 h of feeding on dsVasa. **(D)** Immunofluorescence staining of Vasa protein in the oocyte after RNAi knockdown of Vasa. **(E-F)** The effect after RNAi knockdown of *Vasa* on female fecundity in the S strain. **(G-H)** Average number of ovarioles and oocytes per female sampled on day 3 **(G)** and day 7 **(H)** after RNAi knockdown of *Vasa*. **(I)** Representative images displaying ovary morphology of female adults sampled on day 3 and 7 after RNAi knockdown of *Vasa* in the S strain. Scale bars, 200 μm. Images were acquired at 100X magnification. All qPCR data are presented as the mean ± SEM of at least three independent experiments. All bioassay data (mean ± SEM) represent at least three biological replicates with approximately 60 adults in each replication. The S strain was used in the RNAi experiments. Whiteflies fed on dsEGFP served as a negative control in RNAi experiments. *β*-actin was used as a loading control in western blot analysis. All data (mean ± SEM) on ovariole number and oocyte number represent four biological replicates with at least 15 female individuals in each replication. Asterisks indicate significant differences as determined by Student's *t* test (*$P < 0.05$, **$P < 0.01$, ***$P < 0.001$), and absence of asterisks indicates no statistical significance ($P > 0.05$).

A

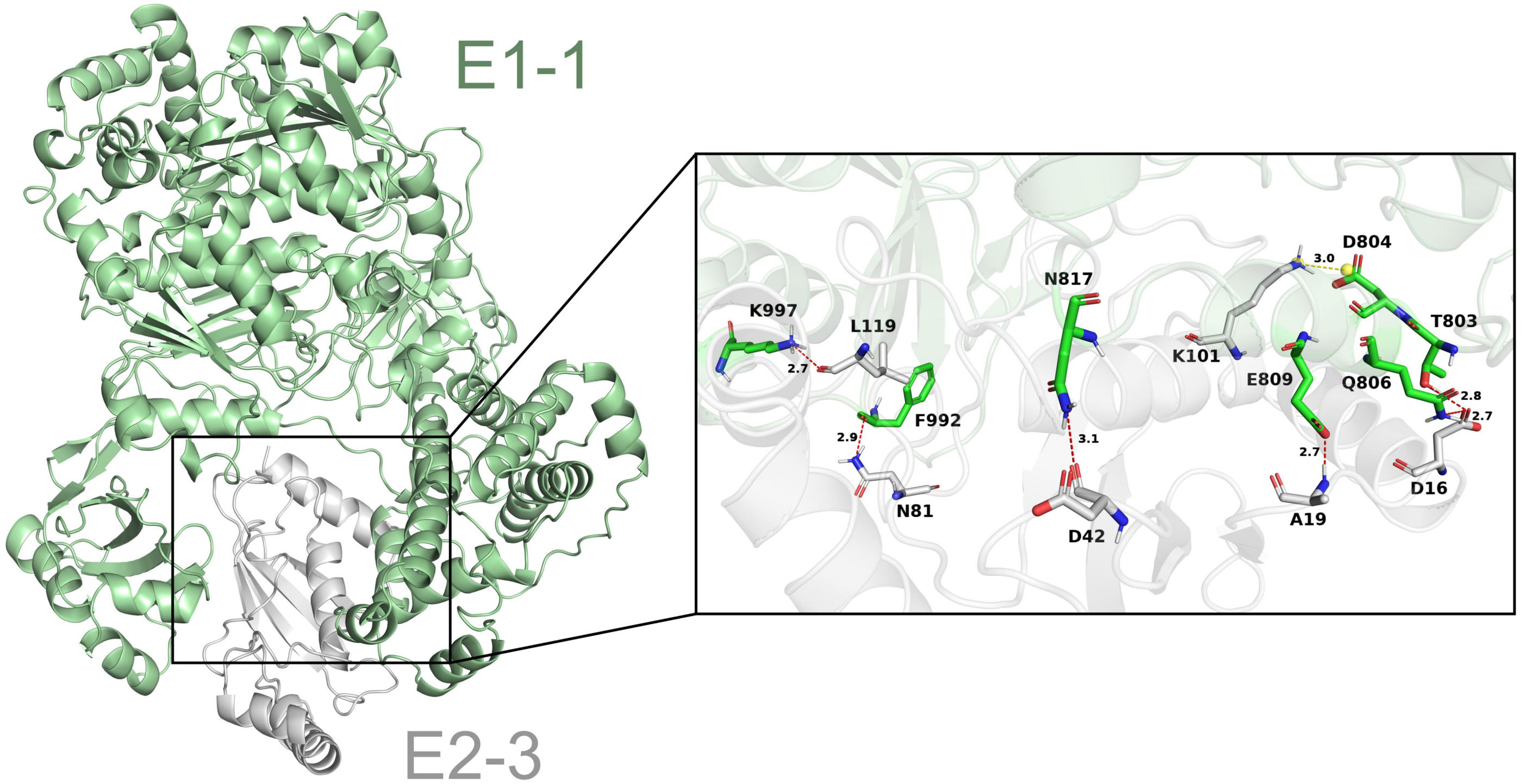
E1-1
E2-3
D804
3.0
N817
K997
L119
T803
K101
2.7
E809
Q806
2.8
2.7
2.9
F992
3.1
2.7
D16
N81
A19
D42

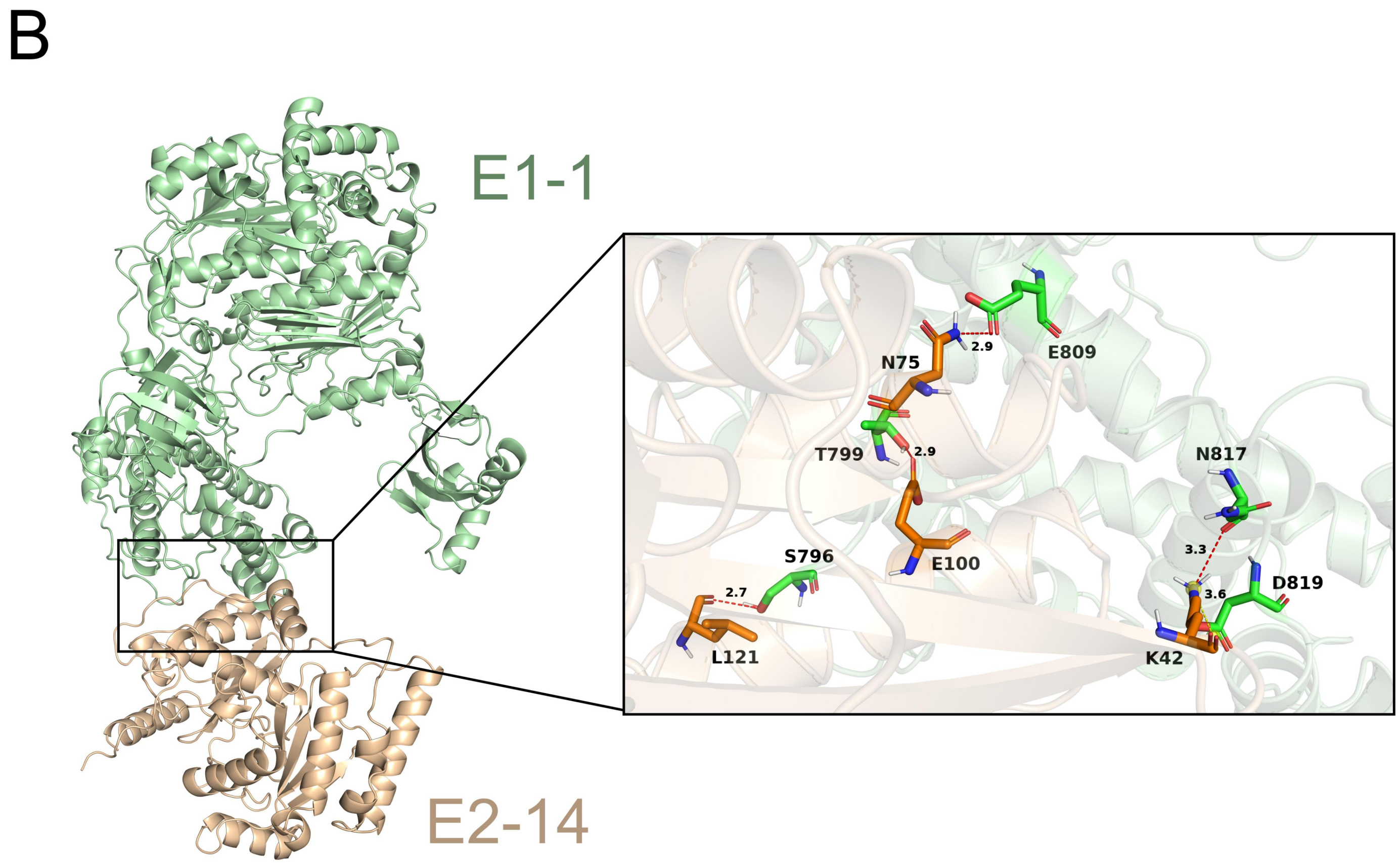
B
E1-1
E2-14
N75
E809
2.9
T799
2.9
N817
S796
E100
3.3
D819
2.7
3.6
L121
K42

C

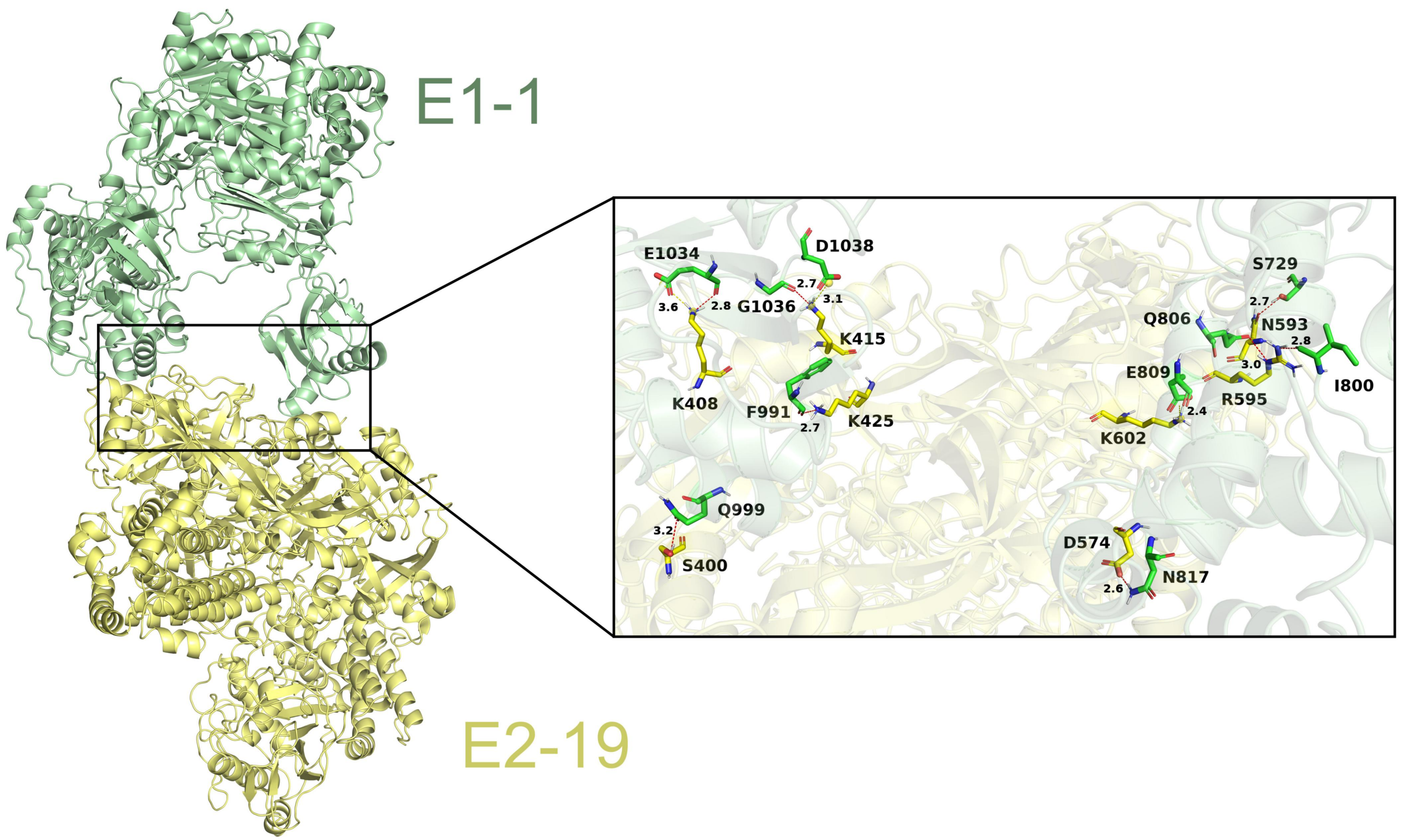

E1-1
E2-19
E1034
D1038
S729
3.6
2.8
G1036
2.7
3.1
Q806
2.7
N593
2.8
K415
E809
3.0
I800
K408
R595
F991
K425
2.7
2.4
K602
Q999
3.2
D574
S400
N817
2.6

D

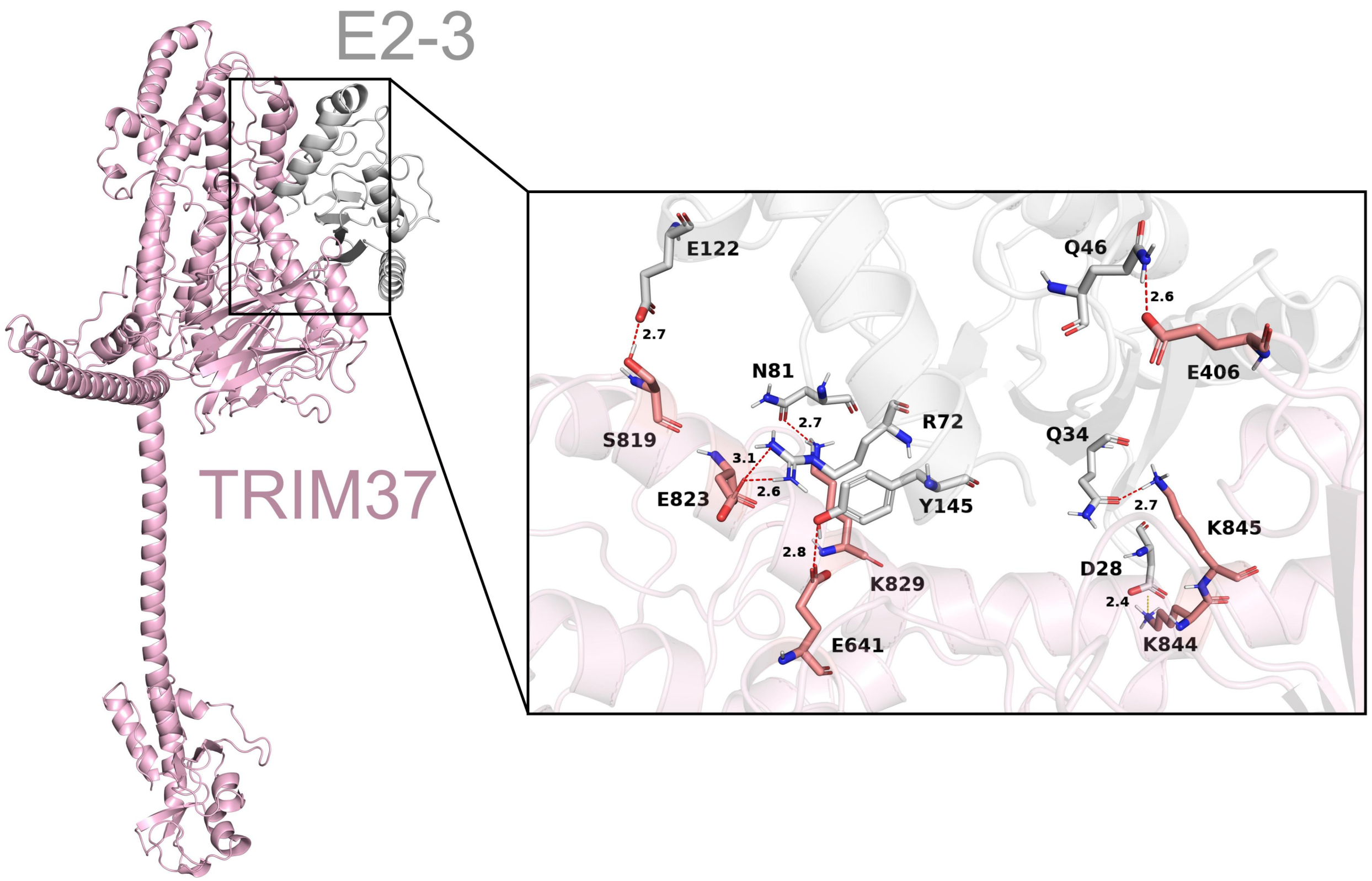

E2-3
TRIM37
E122
2.7
S819
N81
2.7
R72
3.1
E823
2.6
Y145
2.8
K829
E641
Q46
2.6
E406
Q34
2.7
K845
D28
2.4
K844

E

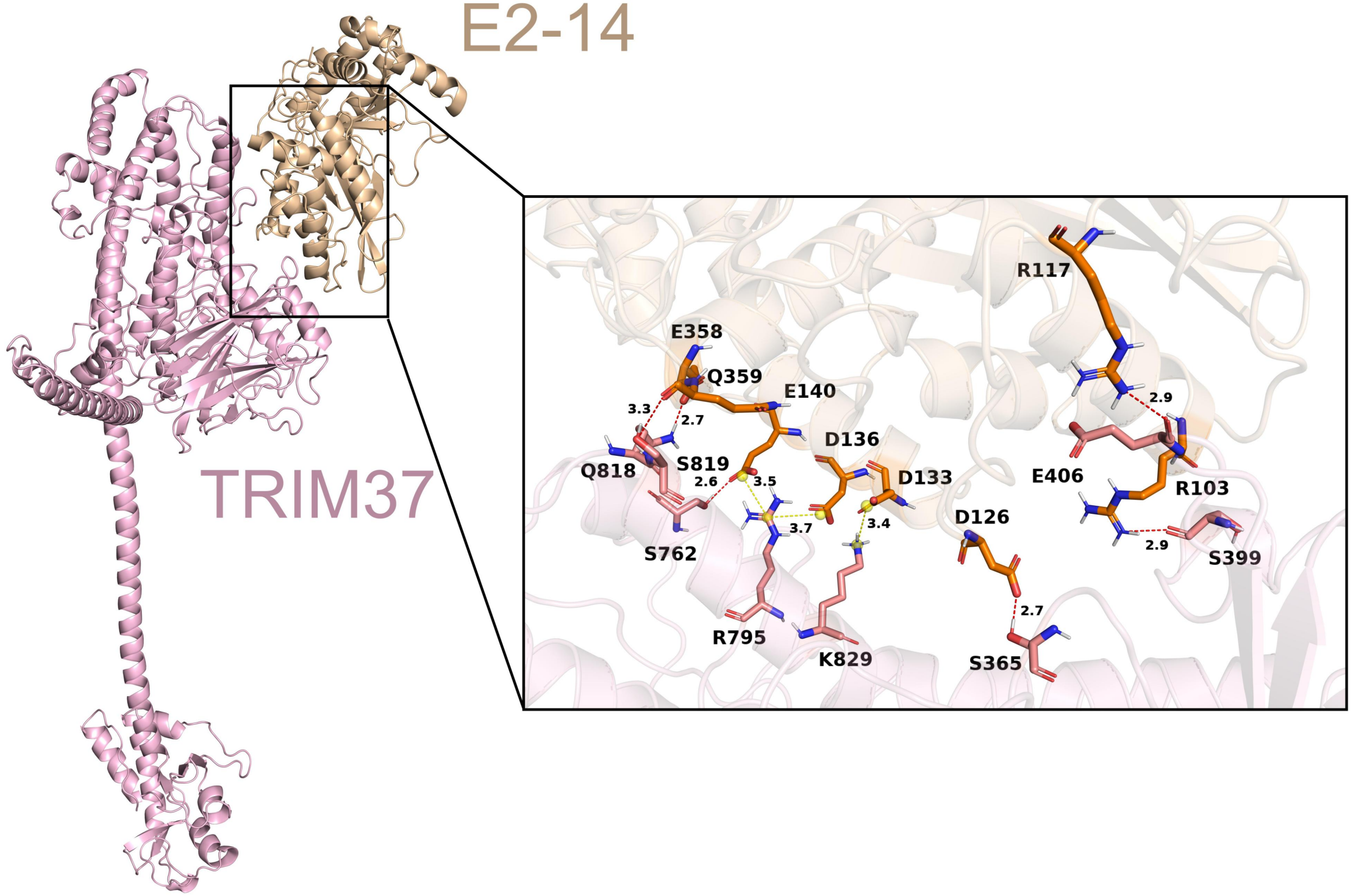
E2-14
TRIM37
R117
E358
Q359
E140
3.3
2.7
2.9
S819
D136
Q818
2.6
3.5
D133
E406
R103
3.7
3.4
D126
S762
2.9
S399
2.7
R795
K829
S365

F

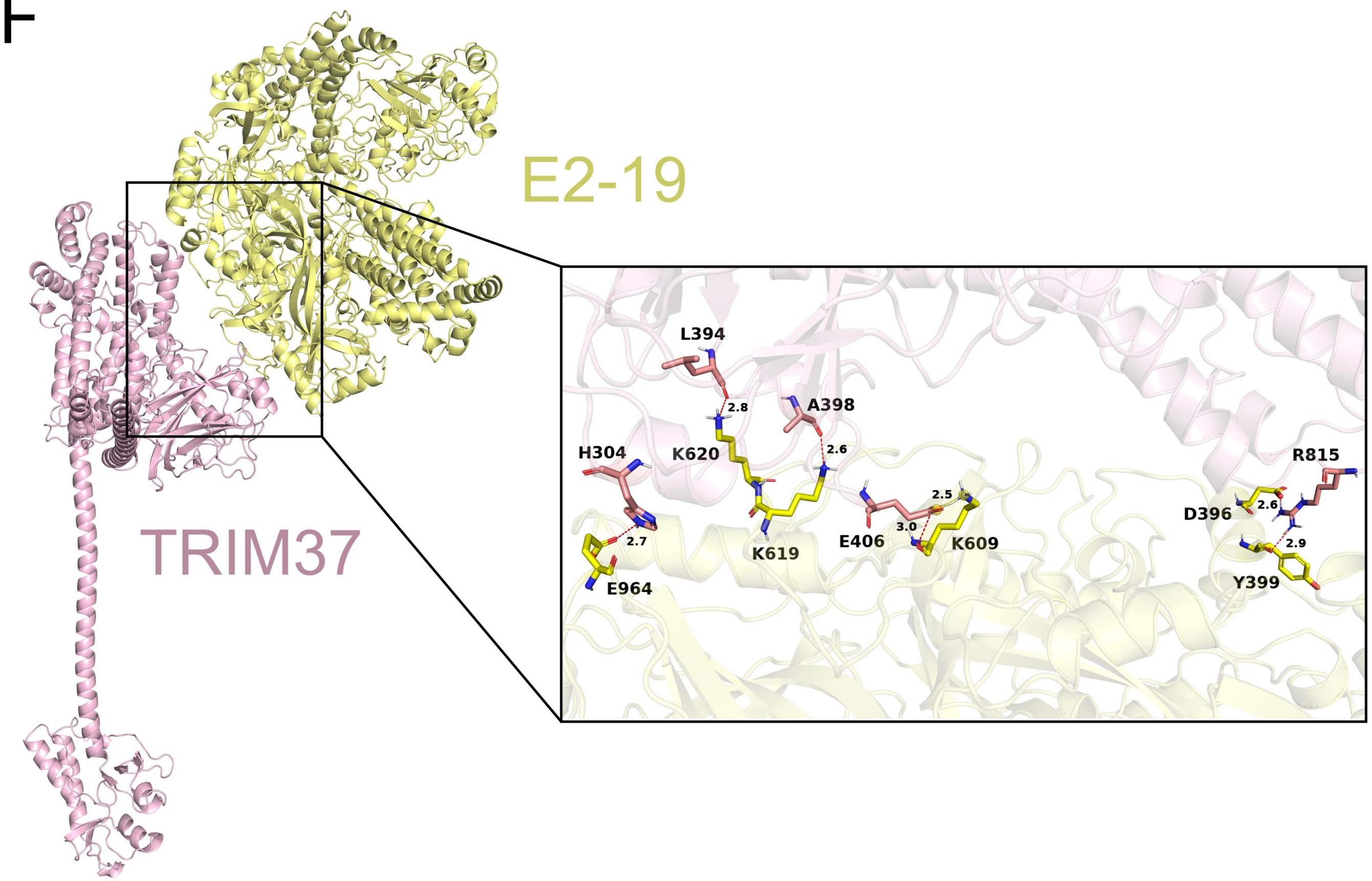

E2-19
TRIM37
L394
2.8
A398
2.6
H304
K620
R815
2.5
D396
2.6
3.0
2.7
E406
K609
2.9
K619
E964
Y399

G

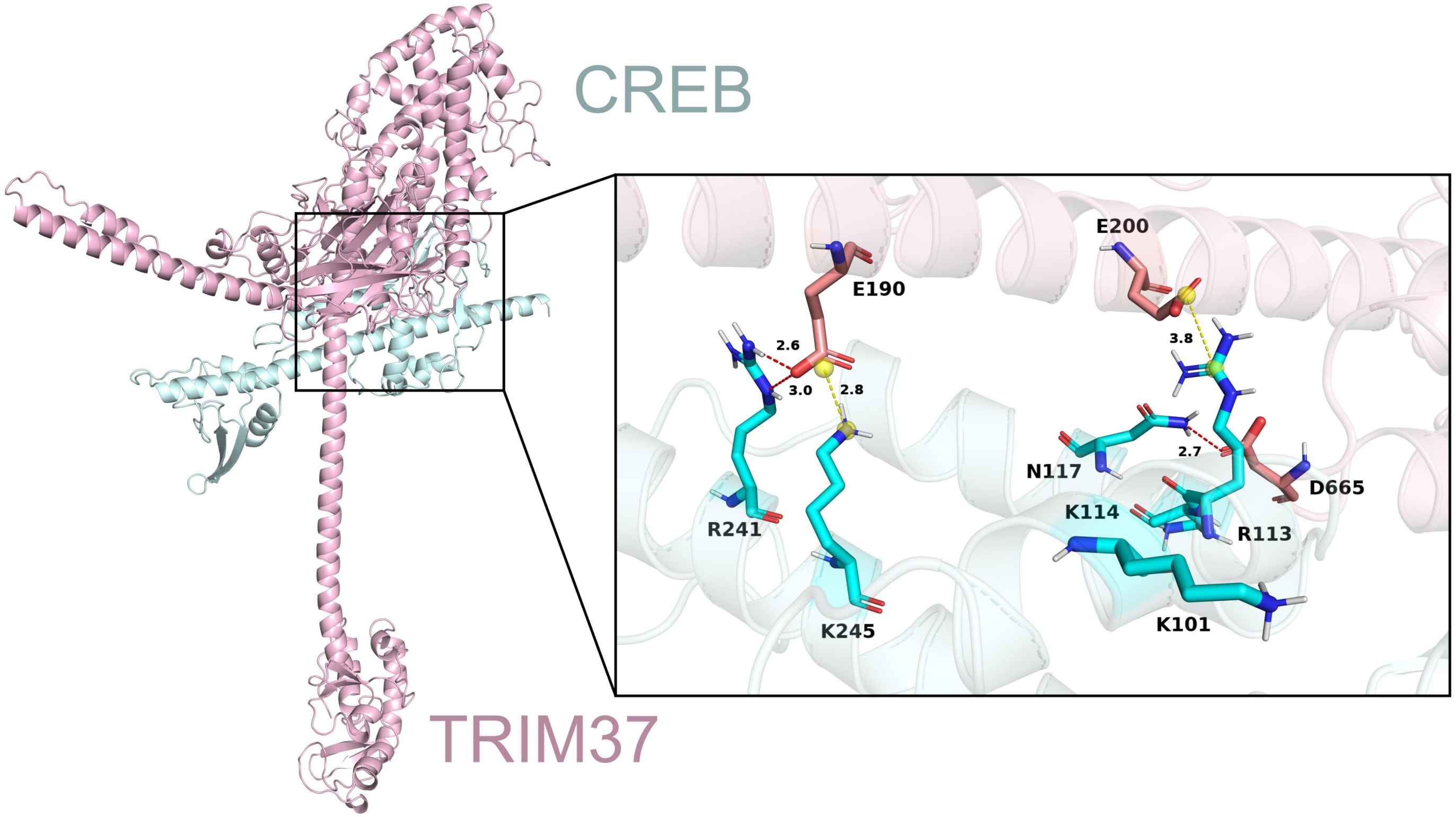

CREB
TRIM37
E200
E190
2.6
3.0
2.8
3.8
2.7
N117
K114
R113
D665
R241
K245
K101

Fig. S8. Predicted binding mode and molecular interactions between E1-1, E2-3/E2-14/E2-19, TRIM37 and CREB. (A) The 3D positions of E1-1 and E2-3. The green protein is E1-1, and the white protein is E2-3. **(B)** The 3D positions of E1-1 and E2-14. The green protein is E1-1, and the orange protein is E2-14. **(C)** The 3D positions of E1-1 and E2-19. The green protein is E1-1, and the yellow protein is E2-19. **(D)** The 3D positions of E2-3 and TRIM37. The white protein is E2-3, and the pink protein is TRIM37. **(E)** The 3D positions of E2-14 and TRIM37. The orange protein is E2-14, and the pink protein is TRIM37. **(F)** The 3D positions of E2-19 and TRIM37. The yellow protein is E2-19, and the pink protein is TRIM37.**(G)** The 3D positions of TRIM37 and CREB. The pink protein is TRIM37, and the blue protein is CREB. The interactions between residues are indicated in the figure, where red dashed lines represent hydrogen bonds, yellow dashed lines represent salt bridges, and the numbers near the dashed lines indicate the bond lengths in angstroms (Å). Detailed residue-level interactions, including hydrogen bonds, salt bridges, distances, and binding energies, are listed in Table S13.

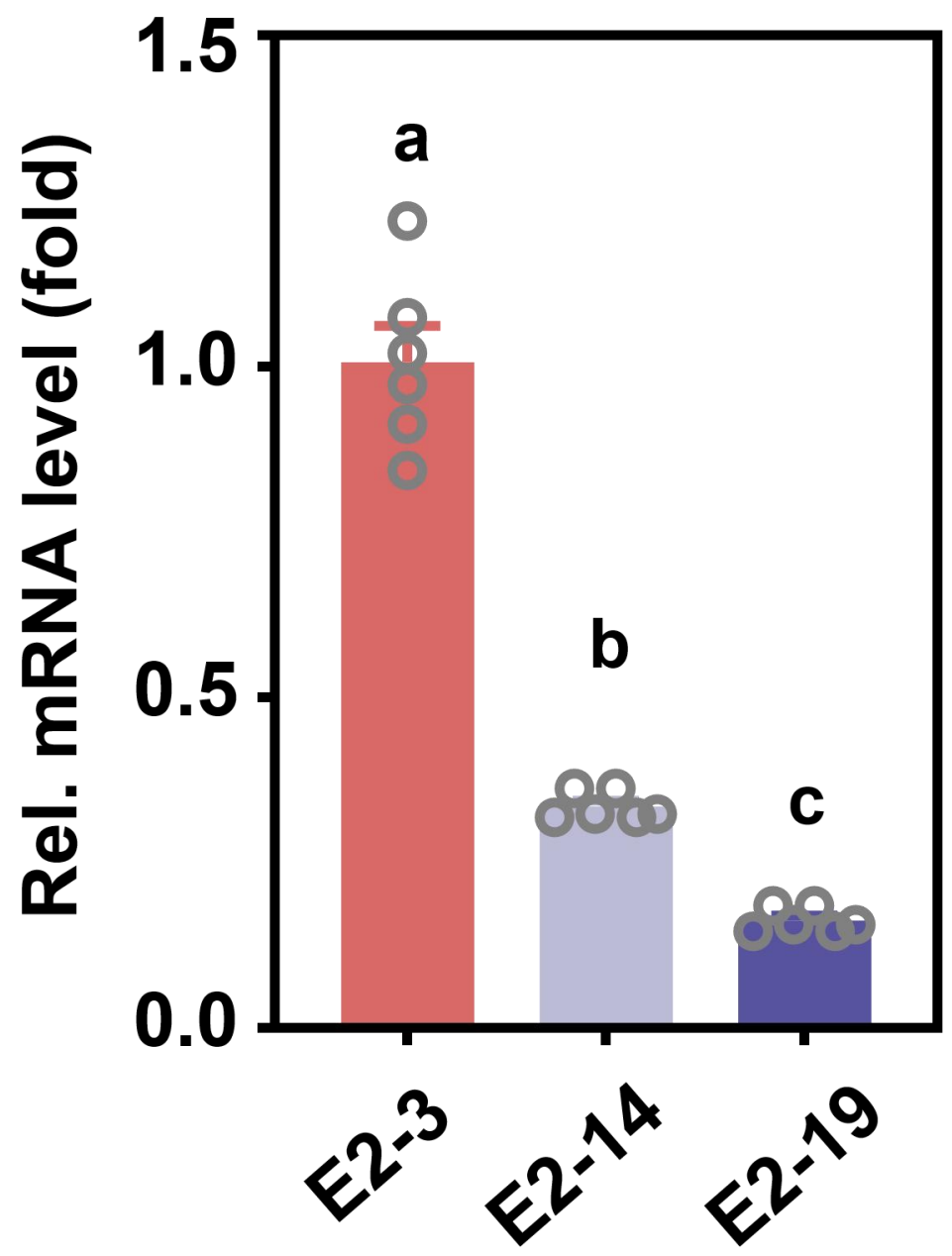

Fig. S9. Relative mRNA expression levels of E2-3, E2-14, and E2-19 in dissected ovaries of adult female *B. tabaci* (susceptible strain S). Total RNA was extracted from ovaries, and qRT-PCR was performed with three biological replicates. Data are presented as mean ± SEM. Different letters (a, b, c) above the bars indicate significant differences (one-way ANOVA with Tukey's HSD test, $P < 0.05$).

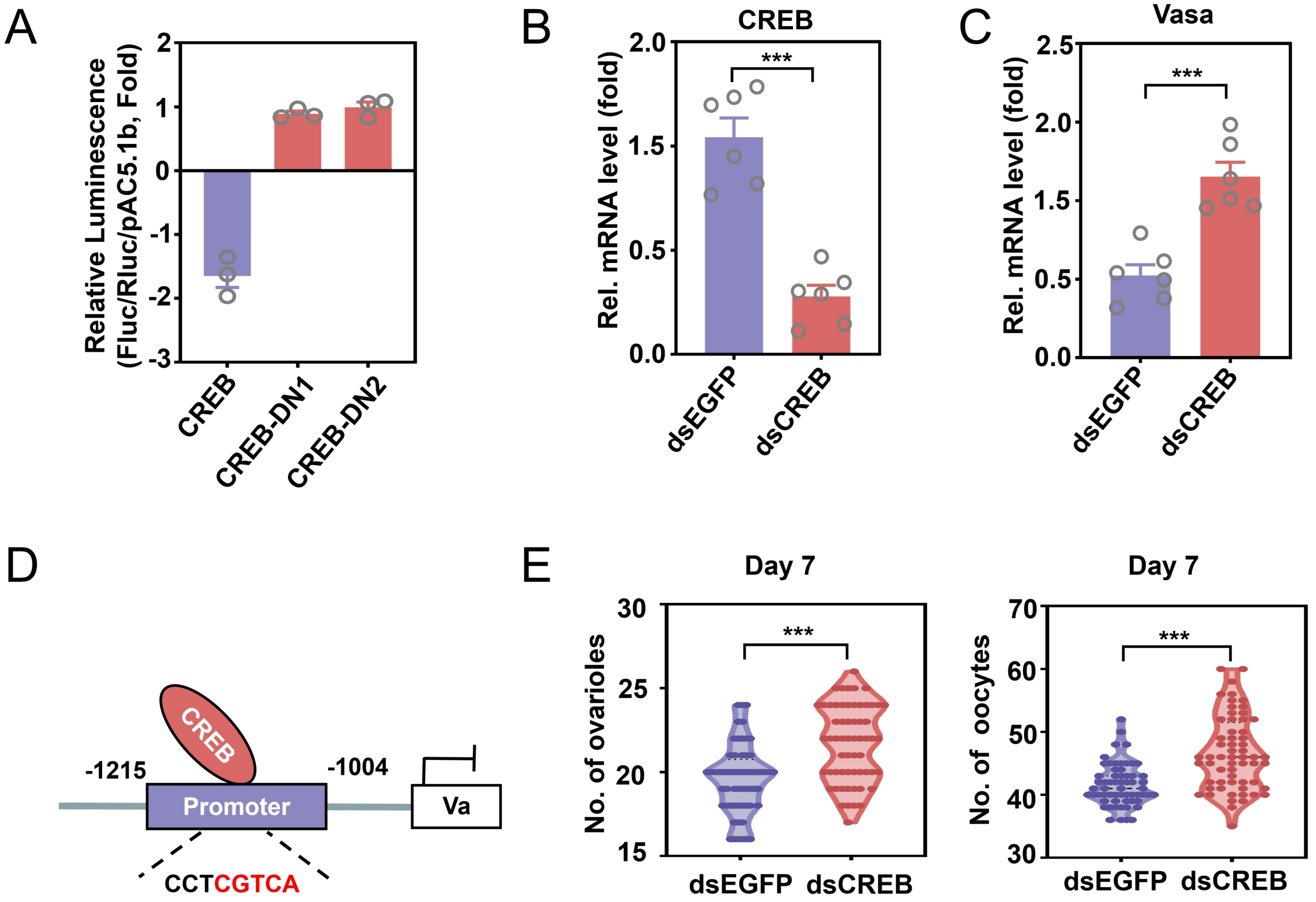

A
Relative Luminescence (Fluc/Rluc/pAC5.1b, Fold)
CREB
CREB-DN1
CREB-DN2
B
CREB
Rel. mRNA level (fold)
***
dsEGFP
dsCREB
C
Vasa
Rel. mRNA level (fold)
***
dsEGFP
dsCREB
D
CREB
-1215
-1004
Promoter
Va
CCTCGTCA
E
Day 7
No. of ovarioles
***
dsEGFP
dsCREB
Day 7
No. of oocytes
***
dsEGFP
dsCREB

Fig. S10. The binding of transcription factor CREB to the core promoter of *Vasa* repress its expression and leads to a reproductive cost. **(A)** Identification of important functional domains in CREB using dual luciferase reporter assays. pGL4.10-Va $^{-1215\ \mathrm{to}\ +1}$ and pGL4.73 were co-transfected into S2 cells with pAC5.1b-CREB (CREB), with modified CREB in which the KID domain (CREB-DN1) or the bZIP domain (CERB-DN2) were deleted, (n = 3, mean ± SE). **(B)** Relative expression analysis of *CREB* after 24 h of feeding on dsCREB. **(C)** Effects after RNAi knockdown of CREB on the expression of *Vasa*. **(D)** Screening of the putative binding sequence of CREB in the core promoter of *Vasa* by dual luciferase reporter gene assays. The predicted binding sequences of CREB were identified using the JASPAR database (https://jaspar.genereg.net/). **(E)** Average number of ovarioles and oocytes per female sampled on day 7 after RNAi knockdown of *CREB*. All qPCR data are presented as the mean ± SEM of at least three independent experiments. All bioassay data (mean ± SEM) represent at least three biological replicates with approximately 60 adults in each replication. The resistant strain $R^{\#1}$ was used in the RNAi experiments. Whiteflies fed on dsEGFP served as a negative control in RNAi experiments. All data (mean ± SEM) on ovariole number and oocyte number represent four biological replicates with at least 15 female individuals in each replication. Asterisks indicate significant differences as determined by Student's *t* test (**P* < 0.05, ***P* < 0.01, ****P* < 0.001), and absence of asterisks indicates no statistical significance (*P* > 0.05).

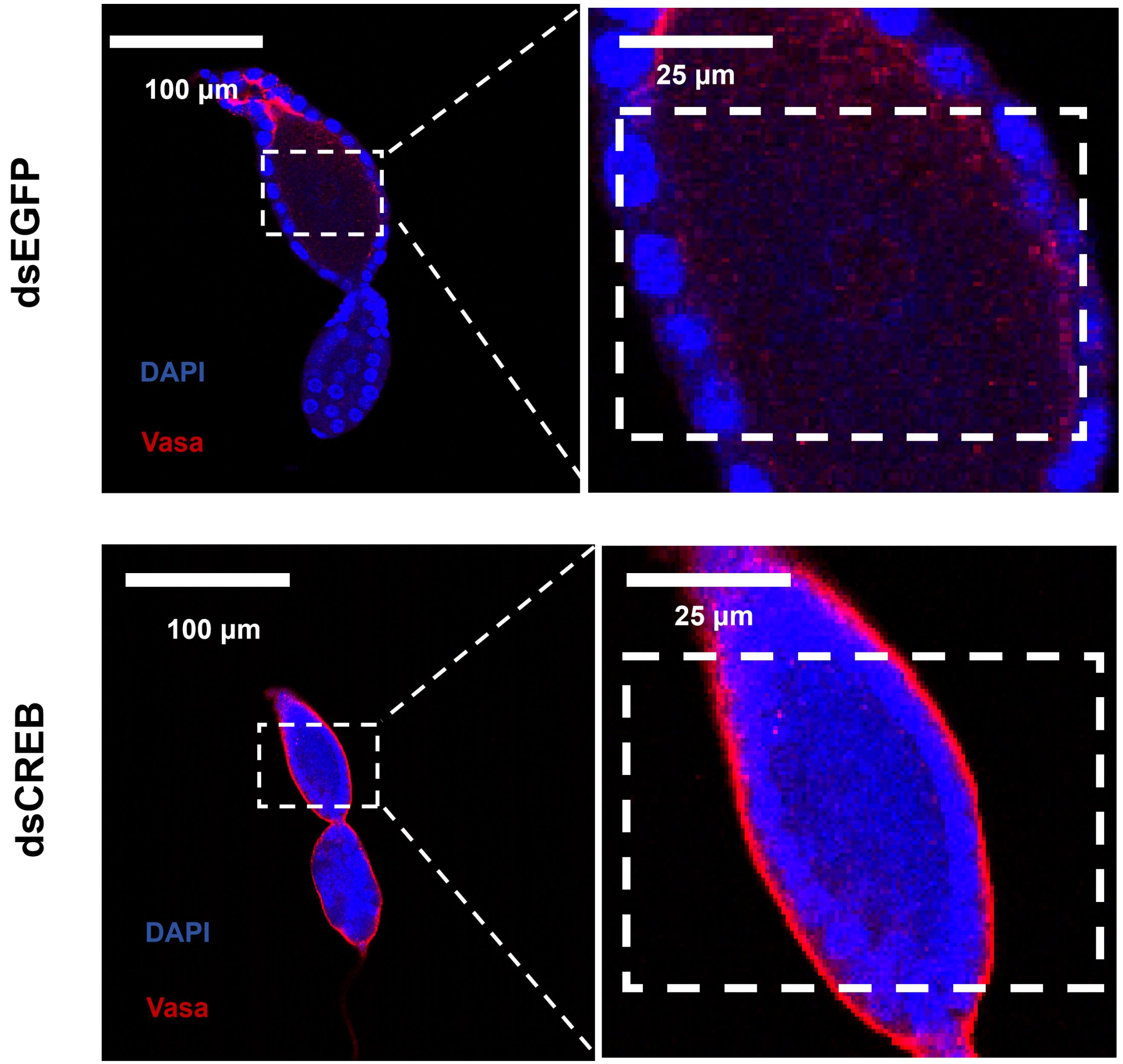

dsEGFP
100 µm
DAPI
Vasa
25 µm
dsCREB
100 µm
DAPI
Vasa
25 µm

Fig. S11. Immunofluorescence staining assays of Vasa protein in oocytes of female *B. tabaci* ovaries after RNAi knockdown of *CREB* in the $R^{\#1}$ strain. Cell nuclei were stained with DAPI (blue). Target protein Vasa was stained with its primary rabbit antibody followed by the secondary antibody Alexa Fluor 555 goat anti-rabbit IgG (red). Whiteflies fed on dsEGFP served as a negative control in the RNAi experiments. Scale bars, 100 μm for original images and 25 μm for zoomed-in images.

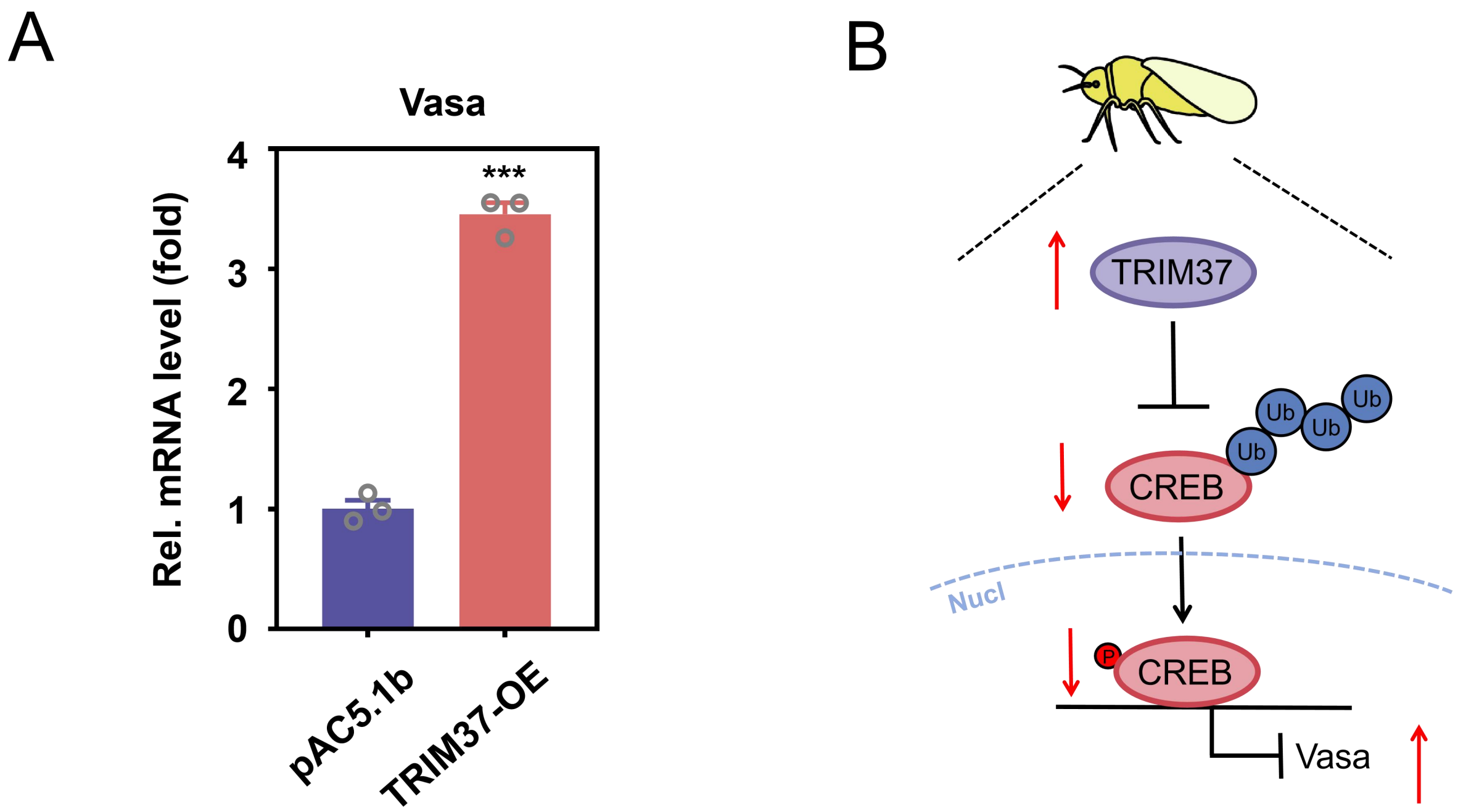

Fig. S12. Model of the regulation of *Vasa* in *B. tabaci*. (A) Effect of TRIM37 overexpression on *Vasa* mRNA levels in S2 cells. *Drosophila* S2 cells were transfected with either empty pAC5.1b vector (control) or pAC5.1b-TRIM37 overexpression construct (TRIM37-OE). After 48 h, the relative mRNA level of *Vasa* was measured by qRT-PCR. All qPCR data are presented as the mean ± SEM of at least three independent experiments. Asterisks indicate significant differences as determined by Student's *t* test (**P* < 0.05, ***P* < 0.01, ****P* < 0.001). **(B)** A model of the regulation of Vasa in *B. tabaci*. In this model, the high-level expression of TRIM37 protein mediates ubiquitination-mediated inhibition of CREB protein, thereby enhancing Vasa transcription.

Table S1 Origin of the experimental strains of the whitefly *Bemisia tabaci* used in this study

Strain code	Sampling location	Host plant	Collection date	Whitefly composition	Strain type
S	Changping, Beijing	Cucumber	2019.07	MED/Q	Field-collected sample
R[#1]	Wuming, Guangxi	Tomato	2021.03	MED/Q	Field-collected sample
R[#2]	Sanya, Hainan	Eggplant	2021.03	MED/Q	Field-collected sample
R[#3]	Sanya, Hainan	Eggplant	2021.03	MED/Q	Field-collected sample

Table S2. Details of technical grade insecticides used in the study

Active agent	Abbr.	Formulation [a]	Group [b]	Manufacturer
Imidacloprid	IMI	95% TC	Neonicotinoids	Shanghai yuanye Bio-Tech. Co., Ltd, China
Thiamethoxam	TMX	95% TC	Neonicotinoids	Shanghai yuanye Bio-Tech. Co., Ltd, China
Clothianidin	CLO	98% TC	Neonicotinoids	Shanghai yuanye Bio-Tech. Co., Ltd, China
Thiacloprid	THIA	97.5% TC	Neonicotinoids	Shanghai yuanye Bio-Tech. Co., Ltd, China
Nitenpyram	NIT	99% TC	Neonicotinoids	Shanghai yuanye Bio-Tech. Co., Ltd, China
Dinotefuran	DIN	95% TC	Neonicotinoids	Shanghai yuanye Bio-Tech. Co., Ltd, China
Acetamiprid	ACE	95.8% TC	Neonicotinoids	Shanghai yuanye Bio-Tech. Co., Ltd, China

a TC indicates technical compounds.

b Group descriptors for all of the active ingredients listed in the table are the insecticide resistance action committee (IRAC) Mode of Action group names.

Table S3 Sensitivity and calculated resistance ratios (RR_{50}) of the experimental strains of *B. tabaci* to seven neonicotinoids and avermectin

Insecticides	Strains	N [a]	Slop±SE	LC_{50} (mg L^{-1}) (95% FL [b])	x^2 [c]	RR_{50} [d]
Imidacloprid (IMI)	S	555	2.32(± 0.23)	10.14(7.37-13.27)	11.08	1
	R[#1]	932	0.78(± 0.08)	125.52(59.21-259.94)	2.736	12.37
	R[#2]	799	1.37(± 0.09)	125.52(185.78-387.08)	14.74	25.57
	R[#3]	386	1.23(± 0.21)	102.15(80.00-141.74)	0.60	10.07
Thiamethoxam (TMX)	S	424	1.43 (± 0.21)	3.31(1.635-5.11)	5.96	1
	R[#1]	663	1.81 (± 0.19)	133.75(116.10-158.00)	0.64	40.44
	R[#2]	895	1.44 (± 0.15)	230.09(141.94-689.66)	6.91	69.58
	R[#3]	491	1.59 (± 0.18)	32.81(15.71-77.26)	10.46	9.92
Clothianidin (CLO)	S	560	1.23 (± 0.19)	1.72(0.62-3.09)	4.01	1
	R[#1]	467	1.34(± 0.14)	163.06(106.68-317.87)	3.33	94.86
	R[#2]	555	1.40(± 0.17)	215.31(160.61-332.05)	1.72	125.25
	R[#3]	547	1.69(± 0.14)	29.88(19.96-40.62)	5.98	17.37
Thiacloprid (THIA)	S	528	1.50 (± 0.11)	0.70(0.13-1.58)	7.17	1
	R[#1]	412	1.18 (±0.14)	13.33(8.18-25.30)	6.40	19.04
	R[#2]	412	1.26 (± 0.14)	19.71(14.80-25.16)	2.03	28.16
	R[#3]	501	1.35 (±0.15)	6.22(3.36-9.23)	4.63	8.89
Nitenpyram (NIT)	S	807	1.77(± 0.11)	0.27(0.14-0.43)	3.43	1
	R[#1]	559	1.70(± 0.18)	118.80(98.01-147.19)	0.44	439.99
	R[#2]	481	1.13(± 0.13)	26.42(20.37-35.32)	1.85	97.87
	R[#3]	743	1.28(± 0.12)	3.659(2.97-4.60)	3.66	13.55
Dinotefuran (DIN)	S	616	2.34(± 0.17)	1.87(1.64-2.11)	0.07	1
	R[#1]	660	1.40 (± 0.11)	22.49(17.59-28.58)	5.29	12.03
	R[#2]	517	2.15(± 0.17)	11.87(10.25-13.71)	1.52	6.35
	R[#3]	416	1.73 (± 0.17)	3.51(2.75-4.35)	1.80	1.88

Insecticides	Strains	N [a]	Slop±SE	LC_{50} (mg L^{-1}) (95% FL [b])	x^2 [c]	RR_{50} [d]
Acetamiprid (ACE)	S	535	1.74(± 0.18)	1.32(0.65-1.95)	1.31	1
	$R^{\#1}$	443	1.56 (± 0.19)	130.24(91.88-227.50)	1.46	98.67
	$R^{\#2}$	728	1.25 (± 0.18)	86.69 (31.20-129.16)	5.97	65.67
	$R^{\#3}$	354	0.91 (± 0.15)	38.38(26.97-59.78)	0.15	29.08

a N, number of adult whiteflies used in each bioassay.

b FL, Fiducial limit.

c x^2 indicates Chi-square.

d RR_{50} , resistance ratio (RR_{50}) = LC_{50} of the other strain / LC_{50} of the most susceptible strain.

Table S4 Developmental durations of the experimental strains of *B. tabaci*

Strains	E (days)	N1 (days)	N2 (days)	N3 (days)	N4 (days)	Preadult (days)
S	6.42 ± 0.05	4.00 ± 0.06	3.49 ± 0.06	3.58 ± 0.06	6.06 ± 0.09	23.31 ± 0.15
(n)	(102)	(85)	(77)	(73)	(68)	(68)
$R^{\#1}$	8.45 ± 0.07	3.86 ± 0.04	3.9 ± 0.04	6.16 ± 0.11	5.89 ± 0.16	28.27 ± 0.14
(n)	(186)	(173)	(157)	(153)	(152)	(152)
$R^{\#2}$	8.68 ± 0.06	2.81 ± 0.05	5.05 ± 0.26	6.91 ± 0.29	3.71 ± 0.11	26.44 ± 0.41
(n)	(199)	(179)	(124)	(92)	(89)	(89)
$R^{\#3}$	8.45 ± 0.053	3.75 ± 0.04	4.06 ± 0.04	6 ± 0.10	5.12 ± 0.11	27.41 ± 0.10
(n)	(215)	(210)	(205)	(205)	(205)	(205)

All biological raw data were analyzed by using the TWO-SEX-MS program. n represents the number of individuals used in life-table study. Data are presented as means ± SEM. Note that the developmental stages of whitefly include egg (E), 1st-instar nymph (N1), 2nd-instar nymph (N2), 3rd-instar nymph (N3) and 4th-instar nymph (N4). Preadult stage contains from egg to adult, also refer to the total immature stage.

Table S5 Reproductive parameters of the experimental strains of *B. tabaci*

Strains	APOP (days)	TPOP (days)	OP (days)	Male longevity (days)	Female longevity (days)	Fecundity (No. of eggs laid/female)	Sex ratio (female%)
S	0.60 ± 0.11	23.98 ± 0.20	28.95 ± 1.27	21.14 ± 0.85 a	32.88 ± 1.39	166.90 ± 6.75	58.82%
$R^{\#1}$	0.4 ± 0.07	28.33 ± 0.22	14.08 ± 0.84	39.66 ± 0.73	43.9 ± 0.79	98.3 ± 6.3	63.16%
$R^{\#2}$	0.19 ± 0.08	26.45 ± 0.51	20 ± 1.12	43.16 ± 1.71	48.29 ± 1.26	137.07 ± 9.34	65.17%
$R^{\#3}$	0.36 ± 0.06	27.82 ± 0.12	13.02 ± 0.62	40.45 ± 0.64	42.5 ± 0.63	79.33 ± 4.03	58.54%

All biological raw data were analyzed by using the TWO-SEX-MS program. Data are presented as means ± SEM. APOP, adult preoviposition period; TPOP, total preoviposition period; OP, oviposition period.

Table S6 Two-sex life table parameters and the relative fitness (*Rf*) of the experimental strains of *B. tabaci*

Strains	r	R_0	λ	T	Rf_r	Rf_{R0}
S	0.1189 ± 0.0044	55.6482 ± 7.5196	1.1263 ± 0.0049	33.7150 ± 0.3370	1	1
$R^{\#1}$	0.1022 ± 0.0031	34.9518 ± 3.6230	1.1076 ± 0.0034	34.7120 ± 0.2640	0.86	0.63
$R^{\#2}$	0.0924 ± 0.0041	27.9062 ± 3.7619	1.0969 ± 0.0045	35.91 ± 0.6980	0.78	0.50
$R^{\#3}$	0.1043 ± 0.0025	35.7933 ± 3.0194	1.1099 ± 0.0027	34.2810 ± 0.2150	0.88	0.64

All biological raw data were analyzed by using the TWO-SEX-MS program. Data were presented as means ± SEM. r, the intrinsic rate of increase; R_0, the net reproductive rate; λ, the finite rate; T, the mean generation time . Rf_r, the relative fitness (*Rf*) calculated with r. Rf_{R0}, the relative fitness (*Rf*) calculated with R_0.

Table S7 Details of nine transcription factors (TFs) analyzed in this study

TF name	Family group[a]	Whitefly Genome or GenBank ID	pI/MW [b] (kDa)	Functional roles
CREB	bZIP	Bta05712	5.40/30	insecticide resistance, reproduction
ERR	NR	MK642614	7.44/52	development, lifespan,reproduction
AhR	bHLH-PAS	Bta13125	6.45/72	xenobiotic responses, resistance
PGRMC1	NR	Bta15630	10.34/24	development, lifespan, reproduction
HR39	NR	MK642605	6.85/77	development, reproduction
HR4	NR	MK642607	9.22/52	development, reproduction
HR78	NR	MK642613	6.37/55	development, reproduction
E63	NR	Bta13636	4.09/16	development, reproduction
KLF11	bZIP	Bta10336	9.18/46	development, lifespan, reproduction

[a] TFs are grouped into three families including nuclear receptor (NR), basic-helix-loop-helix/per- ARNT-SIM (bHLH-PAS) and basic leucine zipper (bZIP).

[b] pI indicates theoretical isoelectric point. MW indicates molecular weight of amino acid.

More detailed information can be found in our published studies by Yang *et al*. (7) and He *et al*. (8).

Table S8 Details of primers used in this study

Usage	Gene	Primer name	Primer sequence (5′-3′)	Product size (bp)	Tm (℃)	Amplification efficiency (%)
qRT-PCR	TRIM37	qTRIM37-F	TGAGGAGTTTGCTGGGTTCT	187	60	97
		qTRIM37-R	TTTGTCCGGATTCCTTTTTG		60	
	Vasa	qVasa-F	ATGGACGACTGGGACTTTAGCG	117	61	104
		qVsaa-R	GCCGTCTTCTTCCTCGTATTGA		60	
	CREB	qCREB-F	ACTCAAGGCAGTCTCCAAACCC	154	60	99
		qCREB-R	TTTCTGCTCCGCCTAAATCGTT		62	
	CYP6CM1	qCM1-F	CACTCTTTTGGATTTACTGCACCC	110	58	99
		qCM1-R	GTGAAGCTGCCTCTTTAATGGC		60	
	EF-1a	EF-F	TAGCCTTGTGCCAATTTCCG	110	60	103
		EF-R	CCTTCAGCATTACCGTCC		60	
	RPL29	RP-F	TCGGAAAATTACCGTGAG	144	60	101
		RP-R	GAACTTGTGATCTACTCCTCTCGTG		60	
	Dm-RPL32	Dm-RPL32-F	GACAGTATCTGATGCCCAACATC	170	59	99
		Dm-RPL32-R	CTTCTTGGAGGAGACGCCGT		61	
**Usage**	**Gene / Whitefly genome ID**	**Primer name**	**Primer sequence (5′-3′)**	**Product size (bp)**	**Tm (℃)**	**Amplification efficiency (%)**
qRT-PCR (E1s and E2s)	E1-1 / Bta05537	qE1-1-F	CGGGGAATTGAACAGCTACG	174	60	97
		qE1-1-R	CCCCTGGTATCTGCGATGAT		60	
	E1-2 / Bta02467	qE1-2-F	TCCAGGAGTGTTTGAGAGCA	201	59	99
		qE1-2-R	TTTCCGACATGTTGCTTTTG		60	
	E2-1 / Bta07316	qE2-1-F	GGTGGCTCGTACAAAATCCG	92	60	101
		qE2-1-R	TTAGGATGGAAGAGCGGAGG		60	
	E2-2 / Bta11662	qE2-2-F	ACTACTGGCGGTGTTGTGAT	139	60	99
		qE2-2-R	TGCCAGTCCAGTGAGTAAGG		60	
	E2-3 / Bta08308	qE2-3-F	TTCTTCGATCGCAGTGGTCT	99	60	100
		qE2-3-R	GCACTAGTGGATCGTCTGGA		60	

E2-4 / Bta06597	qE2-4-F	AACAGAGGAAGCGTTGCGT	94	59	102
	qE2-4-R	GTCTTGTGCTTCATCCTCGC		60	
E2-5 / Bta15228	qE2-5-F	AGGTGCCTTCAGAATCGAGA	148	60	99
	qE2-5-R	TGGTTTCCAATGCTCGGGA		60	
E2-6 / Bta08131	qE2-6-F	ACCTCCAGACACTCCTTACG	170	61	101
	qE2-6-R	CTGCCCATTGGTCTTTGAGG		60	
E2-7 / Bta11302	qE2-7-F	CCGCACTGACCATTTCCAAA	129	60	101
	qE2-7-R	CAATCCTGTCGTGCTCTTCG		60	
E2-8 / Bta01489	qE2-8-F	ACTTTGTATGAGGGCGGGTT	155	60	103
	qE2-8-R	TCACCGGGCTCATGAAGAAT		61	
E2-9 / Bta10621	qE2-9-F	TGCACTTCAAATACGCACCG	149	60	99
	qE2-9-R	ACTTGCGGGTCCATTCTTTC		60	
E2-10 / Bta08538	qE2-10-F	ACTATCCTCTGAGCCCTCCA	144	59	102
	qE2-10-R	TCCACCTTTCAGCACTCGAT		60	
E2-11 / Bta04861	qE2-11-F	GATCGATCGCAGTACCAACC	134	59	97
	qE2-11-R	GCTTGAATTCGTGCAGATGG		60	
E2-12 / Bta08866	qE2-12-F	TCGCCGTATGAGAATGGAGT	155	60	100
	qE2-12-R	TTTGGTGGCATCTTGAGCAG		60	
E2-13 / Bta10908	qE2-13-F	GCGGTTATCCTTATTCGGCC	166	61	103
	qE2-13-R	GGTTCTCCCAGCAGTGACT		60	
E2-14 / Bta11005	qE2-14-F	TTGGTTGGAGGCGCTATTTG	168	60	98
	qE2-14-R	AGGGTGCGTACAGTTTAGCT		60	
E2-15 / Bta02883	qE2-15-F	ATAGGGCCGAACATTCCAGT	121	60	98
	qE2-15-R	AGATGATGGACAGGCAGACG		59	
E2-16 / Bta02881	qE2-16-F	ATCCAACATCCTCGAGTGGT	90	60	100
	qE2-16-R	GGAAACACTAATCGGCCGAG		60	
E2-17 / Bta03029	qE2-17-F	ACGAGATTGGAAACCTGACCT	104	60	100
	qE2-17-R	TGCCTGCCTCCTCATTTAGT		60	
E2-18 / Bta14417	qE2-18-F	GGCTCAACAACCAACAGGAA	101	60	98
	qE2-18-R	CATCAGCTAACTTGACGCGA		61	

	E2-19 / Bta13923	qE2-19-F	AGTTTGCGTCAGTCTGCTTG	172	59	98
		qE2-19-R	TGAATGGCCTTGCTGTGTTC		60	
	E2-20 / Bta07389	qE2-20-F	CGTCTGATGCGTGAATCCTC	178	60	100
		qE2-20-R	GAGGCTGCATTGGGTATTCG		60	
	E2-21 /	qE2-21-F	CAACCACAAATGCGGGATGA	92	60	104
		qE2-21-R	ATTCCTTCTGGCGGTTCTGA		59	

**Usage**	**Genes**	**Primer name**	**Primer sequence (5′-3′)**	**Product size (bp)**	**Tm (℃)**	**Whitefly genome ID**
Gene cloning	TRIM37	TRIM37-F	TTTGATAGCGGGCTCTCGTC	2625	60	Bta11294
		TRIM37-R	AGTGCTGCGTCATTGAAGAGG		60	
	Vasa	Va-F	ATGGACGACTGGGACTTTAGC	1860	60	Bta03359
		Va-R	TTACCACGCTTCTTCTTCTTCTTGT		61	
	CREB	CREB-F	ATGGACGGGATGGTGGAGG	849	62	Bta05712
		CREB-R	TCAATCTGTTTTTTGCTGGCAATAGAGC		63	
	AhR	AhR-F	ATGTCTGCATTAGCTCATTCTGTCCTT	1092	64	Bta13125
		AhR-R	CTCAAAGGGCCCATTGAACATAT		62	
	E63	E63-F	ATGGCTGATCAGCTGACGG	1965	59	Bta13636
		E63-R	CTTTGATGTCATCATTGTCACAAAC		59	
	ERR	ERR-F	ATGAGGAAAAAAACAAAGACAGCAA	1380	60	Bta03309
		ERR-R	TCAATGTAAACAAGCTTCTAACATTTCA		60	
	HR38	HR38-F	ATGATTCTCGCGTGGCGGGG	2271	62	Bta11834
		HR38-R	AAATGGTAAACTAGCAACAAACATGT		59	
	HR39	HR39-F	ATGGTTTGGGGGAAGTACGC	2079	58	Bta09497
		HR39-R	TTAGTGATCACCGCGAAGTAATTC		61	
	HR4	HR4-F	ATGGAGGATTTACATCTCATCAAAT	2268	60	Bta02331
		HR4-R	ATTATAAAATCGGTGCGGAGTG		59	
	KLF11	KLF11-F	ATGGTGCTCACTATTGATACTAGCG	1248	60	Bta10336
		KLF11-R	AATCATTTGAGTGAGGAAGTTCTTC		58	

**Usage**	**Gene**	**Primer name**	**Primer sequence (5′-3′)**	**Product size (bp)**	**Position**	**Vector**

Promoter analysis	Va	P1-F	TACCTTTTTTTTACCCCTGCG	2060	-2060/-1	pGL4.10 recombinant
		P2-F	AGCTTTGTCTTCGCTACCTGTG	1821	-1821/-1	
		P3-F	GGACCAAGCCCTCTGACTGTT	1412	-1412/-1	
		P4-F	GAAATTCCGTCAGAAGGTCTGG	1215	-1215/-1	
		P5-F	AGAATAATTCCTTGTCGCTCGG	1004	-1004/-1	
		P6-F	TAGTAGTACCGTTCGGATTCATTTG	803	-803/-1	
		P7-F	CGAGACCTTCCGACTCATCAC	658	-658/-1	
		P8-F	AAACCTGGTCTTGCCAACATC	409	-409/-1	
		P9-F	GCACCATTTCTCCACCTTGTTA	223	-223/-1	
		Va-R	AGTGTCACCGCTAAAGTCCCAG			

**Usage**	**Genes**	**Primer name**	**Primer sequence (5′-3′)**	**Position**	**Product size (bp)**	**Binding site (5′-3′)**
ChIP-qPCR	Va core promoter	qChIP-Va-F	GTGCGGAAATTCCGTCAG	-1219/-1028	191	AAGTGCTGAATTTTT
		qChIP-Va-R	GGCTGGCCAAAAATCAGT			

**Usage**	**Genes**	**Primer name**	**Primer sequence (5′-3′)**	**Product size (bp)**
RNAi	TRIM37	dsTRIM37-F	TAATACGACTCACTATAGGAATGCTGTAAAAGGTAGCTCTTCA	336
		dsTRIM37-R	TAATACGACTCACTATAGGACTATCACTAAATGTTGGACTGGC	
	Va	dsVa-F	TAATACGACTCACTATAGGGAGAGGCGAAGTGGACCCCAATAA	425
		dsVa-R	TAATACGACTCACTATAGGGAGATGGATGACTGGCAGAAGAAAGG	
	CYP6CM1	dsCM1-F	TAATACGACTCACTATAGGGACTTTTTCAGGGAGGCCATT	378
		dsCM1-R	TAATACGACTCACTATAGGGGTCGCAGCGTCTCATCAATA	
	CREB	dsCREB-F	TAATACGACTCACTATAGGGAAGCGGTGACCCTTTATCCT	365
		dsCREB-R	TAATACGACTCACTATAGGGTTGACGACTCCACTTTGCAG	

F: forward primer; R: reverse primer.

Whitefly Genome Database: http://www.whiteflygenomics.org/cgi-bin/bta/index.cgi. A 22-bp sequence (TAGCAAAATAGGCTGTCCCCAG) was added to 5'-ends of the forward primers of pGL4.10 recombinants; A 20-bp sequence (CGTCGGTAAAGGCGATGGTG) was added to 5'-ends of the reverse primers of pGL4.10 recombinants. A 21-bp sequence (GACACAAAGCCGCTCCATCAG) was added to 5'-ends of forward primers of pAc5.1b-TF recombinant plasmids; A 22-bp sequence (CCTTAGAAGGCACAGTCGAGGC) was added to 5'-ends of reverse primers of pAc5.1b-TF recombinant plasmids.

Table S9 The predicted binding sequences of CREB in the core promoter region of *Vasa*

Genes	Core promoter region (5′-3′)	Predicted binding sequences	Positions (5′-3′)
Vasa	GAAATTC**CGTCA**GAAGGTCTGGAATTTTTATCATTTTTGTCGCAATTTGAGCGAGAAATTCAAAATTTTGAAATATT TTGAATTTCTTTAAACGAAGGTACTAAAAAAGTGCTGAATTTTTCTGTTGAGGAAGTACTGCATTTATTGGGAATGT ACCTACTGAAAAATTACTGATTTTTGGCCAGCCTATTTTAGTAGACACCCTGAATTG	TTCCGTCA	-1118/ -1114

The underlined region is the predicted binding sequence in the core promoter region of *Vasa*. Note that JASPAR Database was used to predict the binding sequences of the transcription factor in the core promoter of a gene. JASPAR Database: https://jaspar.genereg.net/

Table S10 Details of mutant sequences used in this study

Vector	Genes	Name	Original sequences (5′-3′)	Mutant sequences (5′-3′)	Positions
pGL4.10 recombinant	Vasa promoter	Vasa-M0 (WT)	—	—	-1215/ -1004
		Vasa-M1	GAAAT	AGGGC	-1215/ -1211
		Vasa-M2	AATTC	GGCCT	-1113/ -1109
		Vasa-M3	CGTCA	TACTG	-1108/ -1104
		Vasa-M4	GAAGG	AGGAA	-1103/ -1099
		Vasa-M5	TCTGG	CTCAA	-1098/ -1094
pAc5.1b recombinant	CREB	CREB-M1 (bZIP basic region)	CGACTCAACAAGGAAACGAAGAGATATCCT<br>CACAAGGCGGCCTTCCTATAGAAAAATTTT<br>AAACGATTTAGGCGGAGCAGAAATAGCCG<br>GCTGCAAAGTGGAG	GACGACTCAACAAGGAAACGAAGAGATATCCTCACAAGGCGGCC<br>TTCCTATAGAAAAATTTTAAACGATTTAGGCGGAGCAGAAATAGCC<br>GGCTGCAAAGTGGAG	286-390
		CREB-M2 (pKID domain)	AAGCGTGAACTTAGGTTATTAAAAAATAGG<br>GAAGCAGCACGAGAGTGCAGACGAAAAAA<br>GAAGGAATATATTAAATGTCTAGAAAATAGA<br>GTTGCTGTACTTGAAAATCAAAATAAGGCC<br>TTAATAGATGAGTTGAAATCGCTGAAAGAG<br>CTCTATTGCCAG	GGATACAGGTCCGAACCGCCGGGGGGCGAAAGGATGATGTAGA<br>GACATGAGTAGGGGGGAGGAAGGCGCGCCGGGCACTCGAGGG<br>GCGAGACCATCACGTCCAGGGGCTGGGGCGGAATTCCGGCGA<br>GCAGACCAGGGCTATCAGGGAGATCTCGCCATTGA	673-834
		CREB^{304}	AAA	AGA	301-303
		CREB^{341}	AAA	AAA	340-342
	TRIM37	TRIM37^{MU}	TGTTTCATCTGC	TCTTTCATCTCC	73-84
pGEX-4T-1	TRIM37	TRIM37^{MU}	TGTTTCATCTGC	TCTTTCATCTCC	73-84

Note that mutant sequences of the *Vasa* promoter were used to examine binding sites of CREB in dual-luciferase report assays. The mutant sequences of CREB containing its conserved

motifs were used to determine the importance of CREB in regulating transcripts of *Vasa* in dual-luciferase report assays. A 15-bp sequence (GCTAGCCTCGAGGAT) was added to 5'-ends of the forward primers of pGL4.10 recombinants; A 15-bp sequence (AGGCCAGATCTTGAT) was added to 5'-ends of the reverse primers of pGL4.10 recombinants. A 15-bp sequence (TGGAATTCTGCAGAT) was added to 5'-ends of forward primers of pAc5.1b-TF recombinant plasmids; A 15-bp sequence (GCCACTGTGCTGGAT) was added to 5'-ends of reverse primers of pAc5.1b-TF recombinant plasmids. All point mutants were synthesized by a commercial provider (TIANYI HUIYUAN, China) and were confirmed by DNA sequencing before use.

Table S11 Oligonucleotide probes used in EMSA assays

Genes	Sequences (5′-3′)	Length (bp)	5'-labelled	3'-labelled	Position	Probe type
Vasa	GAAATTC**CGTCA**GAAGGTCTGGAAT	25	/	/	-1215/-1191	Unlabelled probe (cold probe)
	GAAATTC**CGTCA**GAAGGTCTGGAAT	25	Biotin	CY5		Labelled probe
	GAAATTC**TACTG**GAAGGTCTGGAAT	25	Biotin	CY5		Mutated probe

Note that all the oligonucleotide probes covering the putative binding site of CREB in the core promoter region were prepared by gene synthesis.

Table S12 Details of antibodies and used in this study

Antibody	Availability	Peptides/Phosphorylation site/	Source
Ubiquitin	commercial product	(#3936S, Cell Sig. Tech.)	mouse
TRIM37	commercial product	(#PA5-30094, Invitrogen)	rabbit
linkage-specific K48	commercial product	(#PTM-7205, PTM BIO)	rabbit
linkage-specific K63	commercial product	(#PTM-7228, PTM BIO)	rabbit
Vasa	synthesis compound	RNRDDDDDNDGGDSYNRDRDDGGRG (Arg81 to Arg106)	rabbit
T-CREB	synthesis compound	SGDPLSSSPSANTT (Ser11 to Thr25)	rabbit
P-CREB	synthesis compound	DILTRRPSYRKILN (Asp103 to Asn117, P-Ser111)	rabbit
CYP6CM1	synthesis compound	TTPKTPKKITFDTN (Tyr486 to Asp500)	rabbit
β-actin	commercial product	(#ab8227, Abcam)	rabbit
IgR-HRP	commercial product	(#CW0103S, CWBIO)	goat anti-rabbit

Table S13 Details of predicted binding mode and molecular interactions of proteins of interest

Protein A	/ Binding site	Protein B	/ Binding site	Distance(Å)	Interaction type	△ⁱG（kcal / mol）
E1-1	GLN 806	E2-3	ASP 16	2.75	Hydrogen Bond	-24.15
	THR 803		ASP 16	2.79	Hydrogen Bond	
	THR 803		ASP 16	2.79	Hydrogen Bond	
	GLU 809		ALA 19	2.68	Hydrogen Bond	
	ASN 817		ASP 42	3.13	Hydrogen Bond	
	PHE 992		ASN 81	2.86	Hydrogen Bond	
	LYS 997		LEU 119	2.71	Hydrogen Bond	
	ASP 804		LYS 101	2.98	Salt Bridge	
E1-1	ASN 817	E2-14	LYS 42	3.33	Hydrogen Bond	-21.79
	GLU 809		ASN 75	2.94	Hydrogen Bond	
	THR 799		GLU 100	2.92	Hydrogen Bond	
	SER 796		LEU 121	2.66	Hydrogen Bond	
	ASP 819		LYS 42	3.61	Salt Bridge	
E1-1	GLN 999	E2-19	SER 400	3.22	Hydrogen Bond	-26.27
	GLU 1034		LYS 408	2.79	Hydrogen Bond	
	GLY 1036		LYS 415	2.75	Hydrogen Bond	
	PHE 991		LYS 425	2.73	Hydrogen Bond	
	ASN 817		ASP 574	2.64	Hydrogen Bond	

	SER 729		ASN 593	2.71	Hydrogen Bond	
	ILE 800		ARG 595	2.75	Hydrogen Bond	
	GLN 806		ARG 595	3.03	Hydrogen Bond	
	GLU 1034		LYS 408	3.57	Salt Bridge	
	ASP 1038		LYS 415	3.09	Salt Bridge	
	GLU 809		LYS 602	2.42	Salt Bridge	
E2-3	GLN 34	TRIM37	LYS 845	2.66	Hydrogen Bond	-24.83
	GLN 46		GLU 406	2.63	Hydrogen Bond	
	ARG 72		GLU 823	2.62	Hydrogen Bond	
	ARG 72		GLU 823	3.14	Hydrogen Bond	
	ASN 81		LYS 829	2.72	Hydrogen Bond	
	GLU 122		SER 819	2.66	Hydrogen Bond	
	TYR 145		GLU 641	2.77	Hydrogen Bond	
	ASP 28		LYS 844	2.43	Salt Bridge	
E2-14	ARG 103	TRIM37	SER 399	2.92	Hydrogen Bond	-25.41
	ARG 117		GLU 406	2.86	Hydrogen Bond	
	ASP 126		SER 365	2.73	Hydrogen Bond	
	ASP 126		SER 365	2.73	Hydrogen Bond	
	GLU 140		SER 762	2.61	Hydrogen Bond	
	GLU 358		GLN 818	2.66	Hydrogen Bond	

	GLN 359		SER 819	3.32	Hydrogen Bond	
	ASP 133		LYS 829	3.42	Salt Bridge	
	ASP 136		ARG 795	3.74	Salt Bridge	
	GLU 140		ARG 795	3.47	Salt Bridge	
	ASP 396		ARG 815	2.63	Hydrogen Bond	
	TYR 399		ARG 815	2.93	Hydrogen Bond	
	LYS 609		GLU 406	3.02	Hydrogen Bond	
E2-19	LYS 619	TRIM37	ALA 398	2.62	Hydrogen Bond	-26.56
	LYS 620		LEU 394	2.77	Hydrogen Bond	
	GLU 964		HIS 304	2.74	Hydrogen Bond	
	LYS 609		GLU 406	2.50	Salt Bridge	
	GLU 190		ARG 241	2.64	Hydrogen Bond	
	GLU 190		ARG 241	3.03	Hydrogen Bond	
TRIM37	ASP 665	CREB	ASN 117	2.72	Hydrogen Bond	-25.37
	GLU 190		LYS 245	2.81	Salt Bridge	
	GLU 200		ARG 113	3.77	Salt Bridge	

Table S14 Information of species sequences used in constructing phylogenetic tree

Protein	Species	Source	Gene ID
TRIM37	*Bemisia tabaci*	NCBI	XP_018900292.1
TRIM37	*Solenopsis invicta*	NCBI	XP_011158206.1
TRIM37_X2	*Harpegnathos saltator*	NCBI	XP_025157581.1
TRIM37	*Habropoda laboriosa*	NCBI	KOC67222.1
TRIM37-like_X3	*Bombus affinis*	NCBI	XP_050577478.1
TRIM37	*Melipona quadrifasciata*	NCBI	KOX68523.1
TRIM37-like X5	*Megalopta genalis*	NCBI	XP_033326855.1
TRIM37	*Frankliniella occidentalis*	NCBI	XP_026271456.1
TRIM37-like	*Thrips palmi*	NCBI	XP_034251412.1
TRIM37_X7	*Bombyx mori*	NCBI	XP_021209126.1
TRIM37-like_X2	*Schistocerca piceifrons*	NCBI	XP_047099049.1
TRIM37	*Cryptotermes secundus*	NCBI	XP_023706712.1
TRIM37-like_X1	*Penaeus vannamei*	NCBI	XP_027215667.1
TRIM37-like_X4	*Portunus trituberculatus*	NCBI	XP_045119066.1
TRIM37-like_X3	*Cherax quadricarinatus*	NCBI	XP_053651703.1
TRIM37_X3	*Tribolium castaneum*	NCBI	XP_972942.1
TRIM37-like_X3	*Onthophagus taurus*	NCBI	XP_022914376.1
TRIM37-like	*Chrysoperla carnea*	NCBI	XP_044734423.1

The multiple sequence alignment used for phylogenetic analysis (Fig. S4C) is provided as Supplementary Data S1.

Table S15 Details of other reagent used in this study

Reagent	Catalog number	Source company
MG132	Cat# 474790	Millipore
DMSO	D8371	Solarbio

Supplemental References

1. B. Fu *et al*., Spinetoram resistance drives interspecific competition between Megalurothrips usitatus and Frankliniella intonsa. *Pest. Manag. Sci.* **78**, 2129-2140 (2022).
2. L. M. Torres-Vila *et al*., Pyrethroid resistance of Helicoverpa armigera in Spain: current status and agroecological perspective. *Agr. Ecosyst. Environ.* **93**, 55-66 (2002).
3. H. Chi, Life-Table analysis incorporating both sexes and variable development rates among individuals. *Environ. Entomol.* **17**, 26-34 (1988).
4. H. Chi, H. Y. Su, Age-stage, two-sex life tables of *Aphidius gifuensis* (Ashmead) (Hymenoptera: Braconidae) and its host *Myzus persicae* (Sulzer) (Homoptera: Aphididae) with mathematical proof of the relationship between female fecundity and the net reproductive rate. *Environ. Entomol*. **35**, 10-21 (2006).
5. J. Guo *et al*., An invasive whitefly feeding on a virus-infected plant increased its egg production and realized fecundity. *PLoS One* **5**, e11713 (2010) .
6. W. Xie *et al*., The invasive MED/Q Bemisia tabaci genome: a tale of gene loss and gene gain. *BMC Genomics* **19**, 68 (2018).
7. X. Yang *et al.*, MAPK-directed activation of the whitefly transcription factor CREB leads to P450-mediated imidacloprid resistance. *Proc. Natl. Acad. Sci. U. S. A.* **117**, 10246-10253 (2020).
8. C. He *et al*., Genome-wide identification and analysis of nuclear receptors genes for lethal screening against Bemisia tabaci Q. *Pest Manag. Sci.* **76**, 2040-2048 (2019).
